# Supplementary material for: The manifold costs of being a non-native English speaker in science
Source: PLoS Biol. 2023 Jul 18;21(7):e3002184. doi: 10.1371/journal.pbio.3002184 (PMC10353817; doi:10.1371/journal.pbio.3002184)
Supplement: S1 Table — The gender composition of the participants was 339 female, 556 male, and 13 participants in other categories, with the median age of 39 (range: 18–77) years old and median 13 (range: 1–55) years of experience in research. (DOCX) [file pbio.3002184.s001.docx]

**S1 Table**. Survey participants by nationality and first language. The gender composition of the participants was 339 female, 556 male and 13 participants in other categories, with the median age of 39 (range: 18 - 77) years old and median 13 (range: 1 - 55) years of experience in research.

| Nationality\First language | Bangla | English | Japanese | Nepali | Spanish | Ukrainian |
| --- | --- | --- | --- | --- | --- | --- |
| Bangladeshi | 106 |  |  |  |  |  |
| Bolivian |  |  |  |  | 100 |  |
| British |  | 112 |  |  |  |  |
| Japanese | 1 | 1 | 292 |  |  |  |
| Nepali |  | 1 |  | 80 | 1 |  |
| Nigerian |  | 40 |  |  |  |  |
| Spanish |  |  |  |  | 107 | 1 |
| Ukrainian |  |  |  |  |  | 66 |
| Total | 107 | 154 | 292 | 80 | 208 | 67 |
