# Supplementary material for: The manifold costs of being a non-native English speaker in science
Source: PLoS Biol. 2023 Jul 18;21(7):e3002184. doi: 10.1371/journal.pbio.3002184 (PMC10353817; doi:10.1371/journal.pbio.3002184)
Supplement: S2 Table — The reference category for English proficiency and Income level was English native and High income, respectively. (DOCX) [file pbio.3002184.s002.docx]

**S2 Table**. Results of a generalised linear model (with a negative binomial distribution) of factors explaining variations in the number of minutes taken to read and understand the entire content of the last English-language original article each participant read in their field. The reference category for English proficiency and Income level was English native and High income, respectively.

| **Variables in the final model** | **Coefficients** | **Standard errors** | **z** | **p** |
| --- | --- | --- | --- | --- |
| Intercept | 3.81 | 0.078 |  |  |
| Low English proficiency | 0.64 | 0.090 | 7.14 | 9.29 × 10^-13^ |
| Moderate English proficiency | 0.39 | 0.099 | 3.95 | 7.79 × 10^-5^ |
| Number of English papers published | -0.00025 | 0.0017 | -0.15 | 0.88 |
| Low English proficiency ×  Number of English papers published | -0.0019 | 0.0032 | -0.60 | 0.55 |
| Moderate English proficiency ×  Number of English papers published | -0.0093 | 0.0029 | -3.25 | 0.0012 |
| Lower-middle income | -0.38 | 0.061 | -6.23 | 4.81 × 10^-10^ |
| **Variables removed based on the likelihood ratio test** | **χ^2^** | **P** |  |  |
| Income level ×  Number of English papers published | 0.062 | 0.80 |  |  |
