# Supplementary material for: The manifold costs of being a non-native English speaker in science
Source: PLoS Biol. 2023 Jul 18;21(7):e3002184. doi: 10.1371/journal.pbio.3002184 (PMC10353817; doi:10.1371/journal.pbio.3002184)
Supplement: S4 Table — The reference category for English proficiency and Income level was English native and High income, respectively. (DOCX) [file pbio.3002184.s004.docx]

**S4 Table**. Result of a generalised linear model (with a negative binomial distribution) of factors explaining variations in the number of days taken to write the first draft of each participant’s latest first-authored paper in English. The reference category for English proficiency and Income level was English native and High income, respectively.

| **Variables in the final model** | **Coefficients** | **Standard errors** | **z** | **p** |
| --- | --- | --- | --- | --- |
| Intercept | 3.29 | 0.085 |  |  |
| Low English proficiency | 0.27 | 0.10 | 2.72 | 0.0066 |
| Moderate English proficiency | 0.41 | 0.11 | 3.85 | 0.00012 |
| Number of English papers published | 0.00016 | 0.0019 | 0.087 | 0.93 |
| Low English proficiency ×  Number of English papers published | -0.0098 | 0.0036 | -2.72 | 0.0066 |
| Moderate English proficiency ×  Number of English papers published | -0.0080 | 0.0032 | -2.50 | 0.012 |
| **Variables removed based on the likelihood ratio test** | **χ^2^** | **P** |  |  |
| Income level | 1.52 | 0.22 |  |  |
| Income level ×  Number of English papers published | 0.28 | 0.60 |  |  |
