# Supplementary material for: The manifold costs of being a non-native English speaker in science
Source: PLoS Biol. 2023 Jul 18;21(7):e3002184. doi: 10.1371/journal.pbio.3002184 (PMC10353817; doi:10.1371/journal.pbio.3002184)
Supplement: S5 Table — The reference category for English proficiency and Income level was English native and High income, respectively. (DOCX) [file pbio.3002184.s005.docx]

**S5 Table**. Result of a generalised linear model (with a negative binomial distribution) of factors explaining variations in the number of days it would take to write the first draft of each participant’s latest first-authored paper in their first language. The reference category for English proficiency and Income level was English native and High income, respectively.

| **Variables in the final model** | **Coefficients** | **Standard errors** | **z** | **p** |
| --- | --- | --- | --- | --- |
| Intercept | 3.25 | 0.089 |  |  |
| Low English proficiency | -0.47 | 0.10 | -4.57 | 4.84 × 10^-6^ |
| Moderate English proficiency | -0.47 | 0.11 | -4.12 | 3.86 × 10^-5^ |
| Number of English papers published | 0.00034 | 0.0019 | 0.18 | 0.86 |
| Low English proficiency ×  Number of English papers published | -0.011 | 0.0039 | -2.93 | 0.0034 |
| Moderate English proficiency ×  Number of English papers published | -0.00063 | 0.0033 | -0.19 | 0.85 |
| Lower-middle income | 0.16 | 0.070 | 2.30 | 0.021 |
| **Variables removed based on the likelihood ratio test** | **χ^2^** | **P** |  |  |
| Income level ×  Number of English papers published | 0.55 | 0.46 |  |  |
