# Supplementary material for: The manifold costs of being a non-native English speaker in science
Source: PLoS Biol. 2023 Jul 18;21(7):e3002184. doi: 10.1371/journal.pbio.3002184 (PMC10353817; doi:10.1371/journal.pbio.3002184)
Supplement: S6 Table — The reference category for English proficiency and Income level was English native and High income, respectively. (DOCX) [file pbio.3002184.s006.docx]

**S6 Table**. Result of a generalised linear model (with a binomial distribution) of factors explaining variations in the percentage of papers where English writing was checked either by someone as a favour or by a paid service. The reference category for English proficiency and Income level was English native and High income, respectively.

| **Variables in the final model** | **Coefficients** | **Standard errors** | **z** | **p** |
| --- | --- | --- | --- | --- |
| Intercept | 0.18 | 0.19 |  |  |
| Low English proficiency | 1.82 | 0.28 | 6.48 | 9.03 × 10^-11^ |
| Moderate English proficiency | 2.60 | 0.31 | 8.38 | < 0.1 × 10^-15^ |
| Number of English papers published | -0.0084 | 0.0054 | -1.54 | 0.12 |
| Low English proficiency ×  Number of English papers published | 0.052 | 0.023 | 2.25 | 0.024 |
| Moderate English proficiency ×  Number of English papers published | -0.0071 | 0.0084 | -0.84 | 0.40 |
| Lower-middle income | -0.66 | 0.21 | -3.12 | 0.0018 |
| **Variables removed based on the likelihood ratio test** | **χ^2^** | **P** |  |  |
| Income level ×  Number of English papers published | 0.16 | 0.69 |  |  |
