# Supplementary material for: The manifold costs of being a non-native English speaker in science
Source: PLoS Biol. 2023 Jul 18;21(7):e3002184. doi: 10.1371/journal.pbio.3002184 (PMC10353817; doi:10.1371/journal.pbio.3002184)
Supplement: S7 Table — The reference category for English proficiency and Income level was English native and High income, respectively. (DOCX) [file pbio.3002184.s007.docx]

**S7 Table**. Result of a generalised linear model (with a binomial distribution) of factors explaining variations in the percentage of papers where English writing was checked by someone as a favour. The reference category for English proficiency and Income level was English native and High income, respectively.

| **Variables in the final model** | **Coefficients** | **Standard errors** | **z** | **p** |
| --- | --- | --- | --- | --- |
| Intercept | 0.45 | 0.031 |  |  |
| Low English proficiency | -0.70 | 0.033 | -21.51 | < 0.1 × 10^-15^ |
| Moderate English proficiency | 0.93 | 0.035 | 26.71 | < 0.1 × 10^-15^ |
| Number of English papers published | -0.019 | 0.0015 | -12.61 | < 0.1 × 10^-15^ |
| Low English proficiency ×  Number of English papers published | -0.0034 | 0.0018 | -1.94 | 0.052 |
| Moderate English proficiency ×  Number of English papers published | 0.0081 | 0.0016 | 4.99 | 5.93 × 10^-7^ |
| Lower-middle income | 0.049 | 0.020 | 2.41 | 0.016 |
| Income level ×  Number of English papers published | -0.0079 | 0.0014 | -5.78 | 7.44 × 10^-9^ |
