# Supplementary material for: The manifold costs of being a non-native English speaker in science
Source: PLoS Biol. 2023 Jul 18;21(7):e3002184. doi: 10.1371/journal.pbio.3002184 (PMC10353817; doi:10.1371/journal.pbio.3002184)
Supplement: S8 Table — The reference category for English proficiency and Income level was English native and High income, respectively. (DOCX) [file pbio.3002184.s008.docx]

**S8 Table**. Result of a generalised linear model (with a binomial distribution) of factors explaining variations in the percentage of papers where English writing was checked by a paid service. The reference category for English proficiency and Income level was English native and High income, respectively.

| **Variables in the final model** | **Coefficients** | **Standard errors** | **z** | **p** |
| --- | --- | --- | --- | --- |
| Intercept | -1.82 | 0.052 |  |  |
| Low English proficiency | 2.67 | 0.054 | 49.29 | < 0.1 × 10^-15^ |
| Moderate English proficiency | 1.76 | 0.056 | 31.29 | < 0.1 × 10^-15^ |
| Number of English papers published | -0.0011 | 0.0018 | -0.61 | 0.54 |
| Low English proficiency ×  Number of English papers published | -6.19 × 10^-6^ | 0.0020 | -0.0030 | > 0.99 |
| Moderate English proficiency ×  Number of English papers published | -0.0034 | 0.0020 | -1.72 | 0.085 |
| Lower-middle income | -2.21 | 0.024 | -90.95 | < 0.1 × 10^-15^ |
| Income level ×  Number of English papers published | 0.0046 | 0.0018 | 2.51 | 0.012 |
