# Supplementary material for: The manifold costs of being a non-native English speaker in science
Source: PLoS Biol. 2023 Jul 18;21(7):e3002184. doi: 10.1371/journal.pbio.3002184 (PMC10353817; doi:10.1371/journal.pbio.3002184)
Supplement: S9 Table — The reference category for English proficiency and Income level was English native and High income, respectively. (DOCX) [file pbio.3002184.s009.docx]

**S9 Table**. Result of a generalised linear model (with a binomial distribution) of factors explaining the experience of having a first-authored English-language paper rejected due to English writing. The reference category for English proficiency and Income level was English native and High income, respectively.

| **Variables in the final model** | **Coefficients** | **Standard errors** | **z** | **p** |
| --- | --- | --- | --- | --- |
| Intercept | -1.78 | 0.25 |  |  |
| Low English proficiency | 1.18 | 0.28 | 4.25 | 2.18 × 10^-5^ |
| Moderate English proficiency | 1.30 | 0.29 | 4.53 | 5.88 × 10^-6^ |
| Number of English papers published | 0.0061 | 0.0043 | 1.43 | 0.15 |
| Low English proficiency ×  Number of English papers published | 0.019 | 0.0094 | 2.00 | 0.046 |
| Moderate English proficiency ×  Number of English papers published | -0.0053 | 0.0070 | -0.76 | 0.45 |
| **Variables removed based on the likelihood ratio test** | **χ^2^** | **P** |  |  |
| Income level | 0.013 | 0.91 |  |  |
| Income level ×  Number of English papers published | 2.00 | 0.16 |  |  |
