# Supplementary material for: The manifold costs of being a non-native English speaker in science
Source: PLoS Biol. 2023 Jul 18;21(7):e3002184. doi: 10.1371/journal.pbio.3002184 (PMC10353817; doi:10.1371/journal.pbio.3002184)
Supplement: S10 Table — The reference category for English proficiency and Income level was English native and High income, respectively. (DOCX) [file pbio.3002184.s010.docx]

**S10 Table**. Result of a cumulative link model of factors explaining the frequency of being requested to improve English writing in the revision of first-authored English-language papers. The reference category for English proficiency and Income level was English native and High income, respectively.

| **Variables in the final model** | **Coefficients** | **Standard errors** | **z** | **p** |
| --- | --- | --- | --- | --- |
| Low English proficiency | 2.36 | 0.20 | 11.87 | < 0.1 × 10^-15^ |
| Moderate English proficiency | 2.08 | 0.21 | 9.97 | < 0.1 × 10^-15^ |
| **Variables removed based on the likelihood ratio test** | **χ^2^** | **P** |  |  |
| Number of English papers published | 0.11 | 0.74 |  |  |
| Income level | 2.31 | 0.13 |  |  |
| English proficiency ×  Number of English papers published | 0.32 | 0.85 |  |  |
| Income level ×  Number of English papers published | 0.68 | 0.41 |  |  |
