# Supplementary material for: The manifold costs of being a non-native English speaker in science
Source: PLoS Biol. 2023 Jul 18;21(7):e3002184. doi: 10.1371/journal.pbio.3002184 (PMC10353817; doi:10.1371/journal.pbio.3002184)
Supplement: S11 Table — The reference category for English proficiency and Income level was English native and High income, respectively. (DOCX) [file pbio.3002184.s011.docx]

**S11 Table**. Result of a generalised linear model (with a binomial distribution) of factors explaining the experience of providing a non-English-language abstract of English-language papers. The reference category for English proficiency and Income level was English native and High income, respectively.

| **Variables in the final model** | **Coefficients** | **Standard errors** | **z** | **p** |
| --- | --- | --- | --- | --- |
| Intercept | -1.33 | 0.22 |  |  |
| Low English proficiency | 0.48 | 0.23 | 2.11 | 0.035 |
| Moderate English proficiency | 1.71 | 0.25 | 6.87 | 6.33 × 10^-12^ |
| Number of English papers published | 0.014 | 0.0039 | 3.62 | 0.00030 |
| Lower-middle income | -0.61 | 0.16 | -3.74 | 0.00019 |
| **Variables removed based on the likelihood ratio test** | **χ^2^** | **P** |  |  |
| English proficiency ×  Number of English papers published | 4.07 | 0.13 |  |  |
| Income level ×  Number of English papers published | 0.40 | 0.53 |  |  |
