# Supplementary material for: The manifold costs of being a non-native English speaker in science
Source: PLoS Biol. 2023 Jul 18;21(7):e3002184. doi: 10.1371/journal.pbio.3002184 (PMC10353817; doi:10.1371/journal.pbio.3002184)
Supplement: S12 Table — The reference category for English proficiency and Income level was English native and High income, respectively. (DOCX) [file pbio.3002184.s012.docx]

**S12 Table**. Result of a generalised linear model (with a binomial distribution) of factors explaining the experience of disseminating English-language papers in other language(s) in addition to English. The reference category for English proficiency and Income level was English native and High income, respectively.

| **Variables in the final model** | **Coefficients** | **Standard errors** | **z** | **p** |
| --- | --- | --- | --- | --- |
| Intercept | -0.72 | 0.19 |  |  |
| Low English proficiency | 0.33 | 0.22 | 1.48 | 0.14 |
| Moderate English proficiency | 0.67 | 0.24 | 2.78 | 0.0055 |
| Number of English papers published | -0.0014 | 0.0045 | -0.30 | 0.76 |
| Low English proficiency ×  Number of English papers published | 0.011 | 0.0082 | 1.33 | 0.18 |
| Moderate English proficiency ×  Number of English papers published | 0.027 | 0.010 | 2.60 | 0.0092 |
| **Variables removed based on the likelihood ratio test** | **χ^2^** | **P** |  |  |
| Income level | 1.87 | 0.17 |  |  |
| Income level ×  Number of English papers published | 1.24 | 0.26 |  |  |
