# Supplementary material for: The manifold costs of being a non-native English speaker in science
Source: PLoS Biol. 2023 Jul 18;21(7):e3002184. doi: 10.1371/journal.pbio.3002184 (PMC10353817; doi:10.1371/journal.pbio.3002184)
Supplement: S13 Table — The reference category for English proficiency and Income level was Low English proficiency and High income, respectively. (DOCX) [file pbio.3002184.s013.docx]

**S13 Table**. Result of a cumulative link model of factors explaining the frequency of not attending an English-language conference due to a lack of confidence in English communication. The reference category for English proficiency and Income level was Low English proficiency and High income, respectively.

| **Variables in the final model** | **Coefficients** | **Standard errors** | **z** | **p** |
| --- | --- | --- | --- | --- |
| Number of English papers published | -0.014 | 0.0049 | -2.82 | 0.0047 |
| Lower-middle income | -0.99 | 0.16 | -6.36 | 2.00 × 10^-10^ |
| **Variables removed based on the likelihood ratio test** | **χ^2^** | **P** |  |  |
| English proficiency | 0.072 | 0.79 |  |  |
| English proficiency ×  Number of English papers published | 1.27 | 0.26 |  |  |
| Income level ×  Number of English papers published | 1.27 | 0.26 |  |  |
