# Supplementary material for: The manifold costs of being a non-native English speaker in science
Source: PLoS Biol. 2023 Jul 18;21(7):e3002184. doi: 10.1371/journal.pbio.3002184 (PMC10353817; doi:10.1371/journal.pbio.3002184)
Supplement: S14 Table — The reference category for English proficiency and Income level was Low English proficiency and High income, respectively. (DOCX) [file pbio.3002184.s014.docx]

**S14 Table**. Result of a cumulative link model of factors explaining the frequency of avoiding oral presentations at an English-language conference due to a lack of confidence in English communication. The reference category for English proficiency and Income level was Low English proficiency and High income, respectively.

| **Variables in the final model** | **Coefficients** | **Standard errors** | **z** | **p** |
| --- | --- | --- | --- | --- |
| Moderate English proficiency | 0.32 | 0.16 | 2.00 | 0.046 |
| Number of English papers published | -0.020 | 0.0050 | -3.99 | 6.67 × 10^-5^ |
| Lower-middle income | -1.37 | 0.17 | -8.00 | 1.22 × 10^-15^ |
| **Variables removed based on the likelihood ratio test** | **χ^2^** | **P** |  |  |
| English proficiency ×  Number of English papers published | 0.049 | 0.83 |  |  |
| Income level ×  Number of English papers published | 0.52 | 0.47 |  |  |
