# Supplementary material for: The manifold costs of being a non-native English speaker in science
Source: PLoS Biol. 2023 Jul 18;21(7):e3002184. doi: 10.1371/journal.pbio.3002184 (PMC10353817; doi:10.1371/journal.pbio.3002184)
Supplement: S16 Table — The reference category for English proficiency and Income level was English native and High income, respectively. The number of English papers published was not significant in the likelihood ratio test but was retained in the final model for a comparison with other results. (DOCX) [file pbio.3002184.s016.docx]

**S16 Table**. Results of a generalised linear model (with a negative binomial distribution) of factors explaining variations in the number of hours that would be taken to prepare and practice the same oral presentation in the first language. The reference category for English proficiency and Income level was English native and High income, respectively. The number of English papers published was not significant in the likelihood ratio test, but was retained in the final model for a comparison with other results.

| **Variables in the final model** | **Coefficients** | **Standard errors** | **z** | **P** |
| --- | --- | --- | --- | --- |
| Intercept | 2.88 | 0.11 |  |  |
| Low English proficiency | -0.41 | 0.12 | -3.58 | 0.00034 |
| Moderate English proficiency | 0.046 | 0.12 | 0.37 | 0.71 |
| Number of English papers published | 0.00031 | 0.0017 | 0.18 | 0.86 |
| Lower-middle income | -0.34 | 0.087 | -3.87 | 0.00011 |
| **Variables removed based on the likelihood ratio test** | **χ^2^** | **P** |  |  |
| English proficiency ×  Number of English papers published | 4.54 | 0.10 |  |  |
| Income level ×  Number of English papers published | 2.80 | 0.095 |  |  |
