# Supplementary material for: The manifold costs of being a non-native English speaker in science
Source: PLoS Biol. 2023 Jul 18;21(7):e3002184. doi: 10.1371/journal.pbio.3002184 (PMC10353817; doi:10.1371/journal.pbio.3002184)
Supplement: S17 Table — The reference category for English proficiency and Income level was Low English proficiency and High income, respectively. (DOCX) [file pbio.3002184.s017.docx]

**S17 Table**. Result of a cumulative link model of factors explaining the frequency of not being able to explain research confidently during a presentation due to English barriers. The reference category for English proficiency and Income level was Low English proficiency and High income, respectively.

| **Variables in the final model** | **Coefficients** | **Standard errors** | **z** | **p** |
| --- | --- | --- | --- | --- |
| Moderate English proficiency | -0.52 | 0.15 | -3.41 | 0.00065 |
| Number of English papers published | -0.017 | 0.0039 | -4.29 | 1.79 × 10^-5^ |
| Lower-middle income | -0.98 | 0.15 | -6.37 | 1.94 × 10^-10^ |
| **Variables removed based on the likelihood ratio test** | **χ^2^** | **P** |  |  |
| English proficiency ×  Number of English papers published | 0.38 | 0.54 |  |  |
| Income level ×  Number of English papers published | 0.82 | 0.36 |  |  |
