# Supplementary figures and images for: The manifold costs of being a non-native English speaker in science
Source: PLoS Biol. 2023 Jul 18;21(7):e3002184. doi: 10.1371/journal.pbio.3002184 (PMC10353817; doi:10.1371/journal.pbio.3002184)

Extra minutes needed to read an English paper  
compared to native English speakers

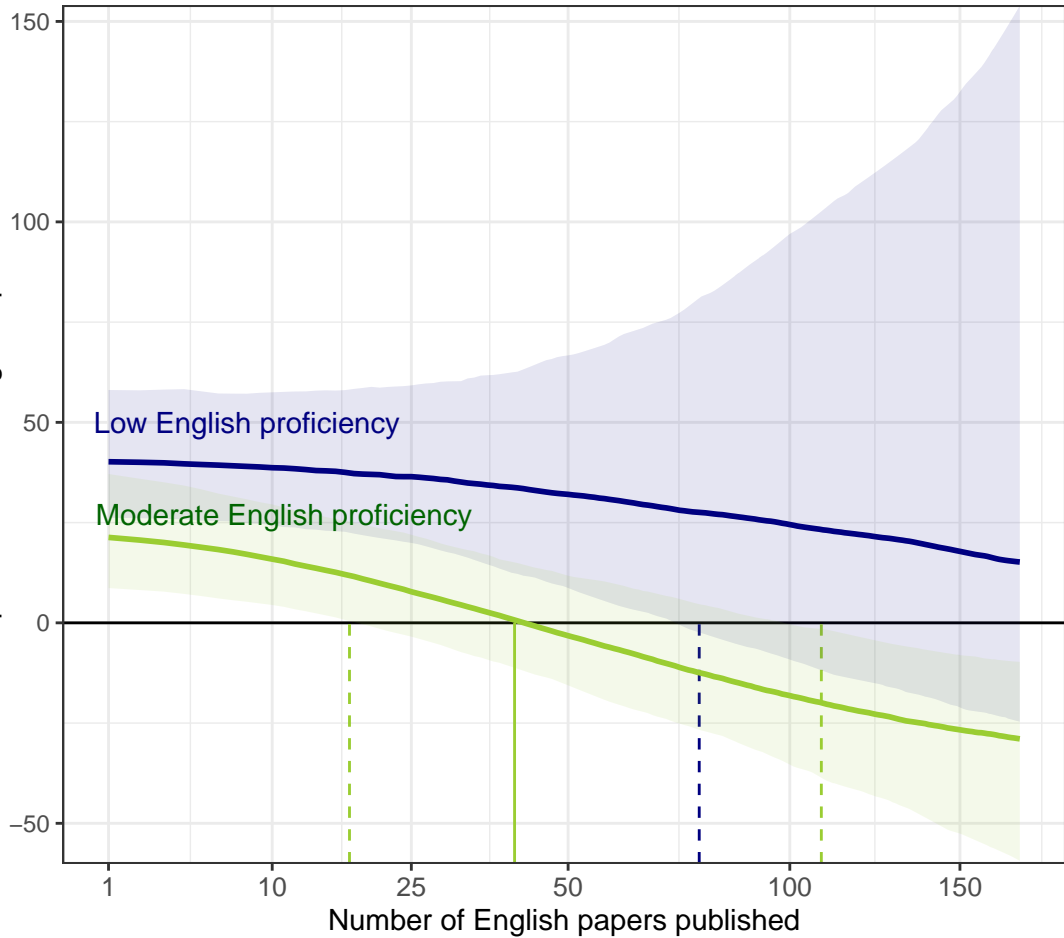

Supplement: S1 Fig — The estimations are based on the results of the regression shown in S2 Table. The solid vertical lines (and 95% confidence intervals as broken vertical lines) indicate the number of English-language papers published, as a measure of career level, where non-native English speakers do not take longer to read an English-language paper than native English speakers. Non-native English speakers who have published only one English-language paper were estimated to require, on average, 40.18 (low English proficiency nationalities) and 21.31 (moderate English proficiency nationalities) more minutes to read an English-language article, compared to their native English-speaking counterparts. If they were to read 200 articles per year (average number of article readings per year for US faculty [51]), this equates to 19.1 (low English proficiency nationalities) and 10.1 (moderate English proficiency nationalities) more working days per year, assuming a 7-hour working day. The data underlying this figure can be found in S1 Data. (PDF) [file pbio.3002184.s018.pdf]

Extra days needed to write an English paper  
compared to native English speakers

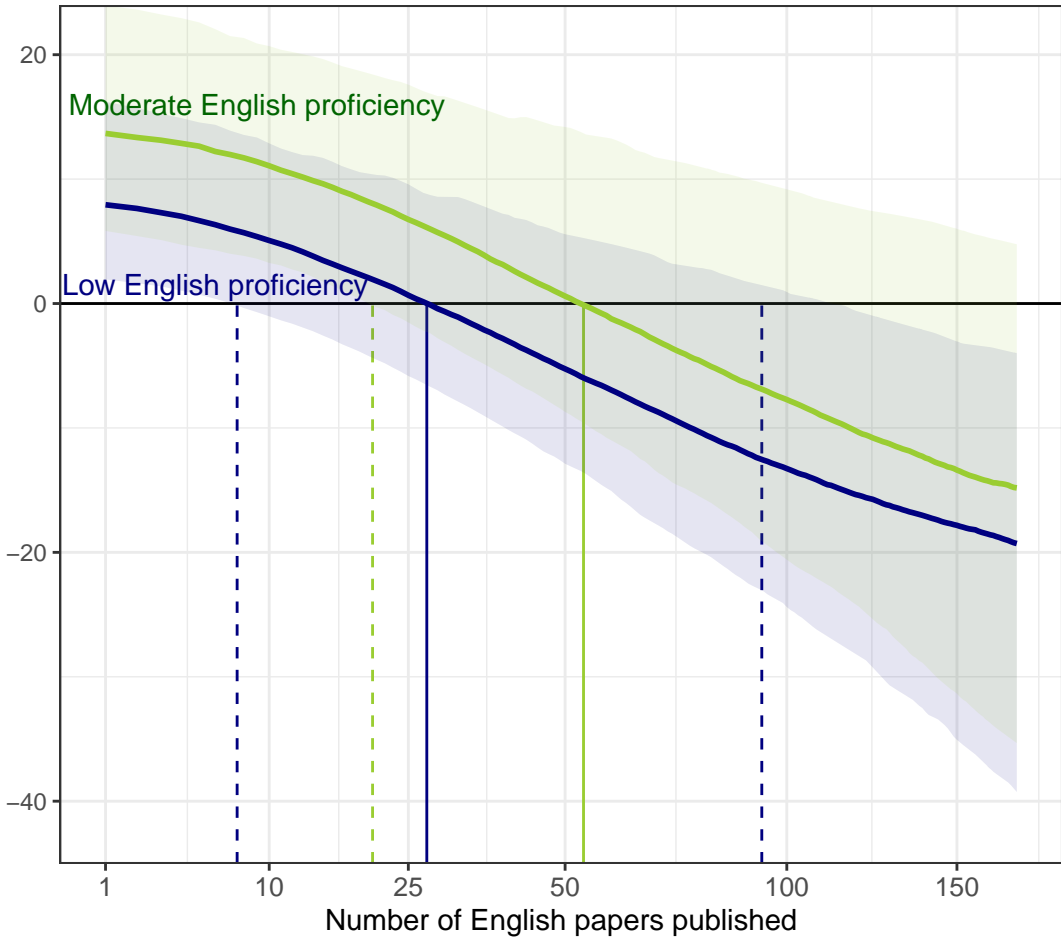

Supplement: S2 Fig — The estimations are based on the results of the regression shown in S4 Table. The solid vertical lines (and 95% confidence intervals as broken vertical lines) indicate the number of English-language papers published, as a measure of career level, where non-native English speakers do not take longer to write an English-language paper than native English speakers. The data underlying this figure can be found in S1 Data. (PDF) [file pbio.3002184.s019.pdf]

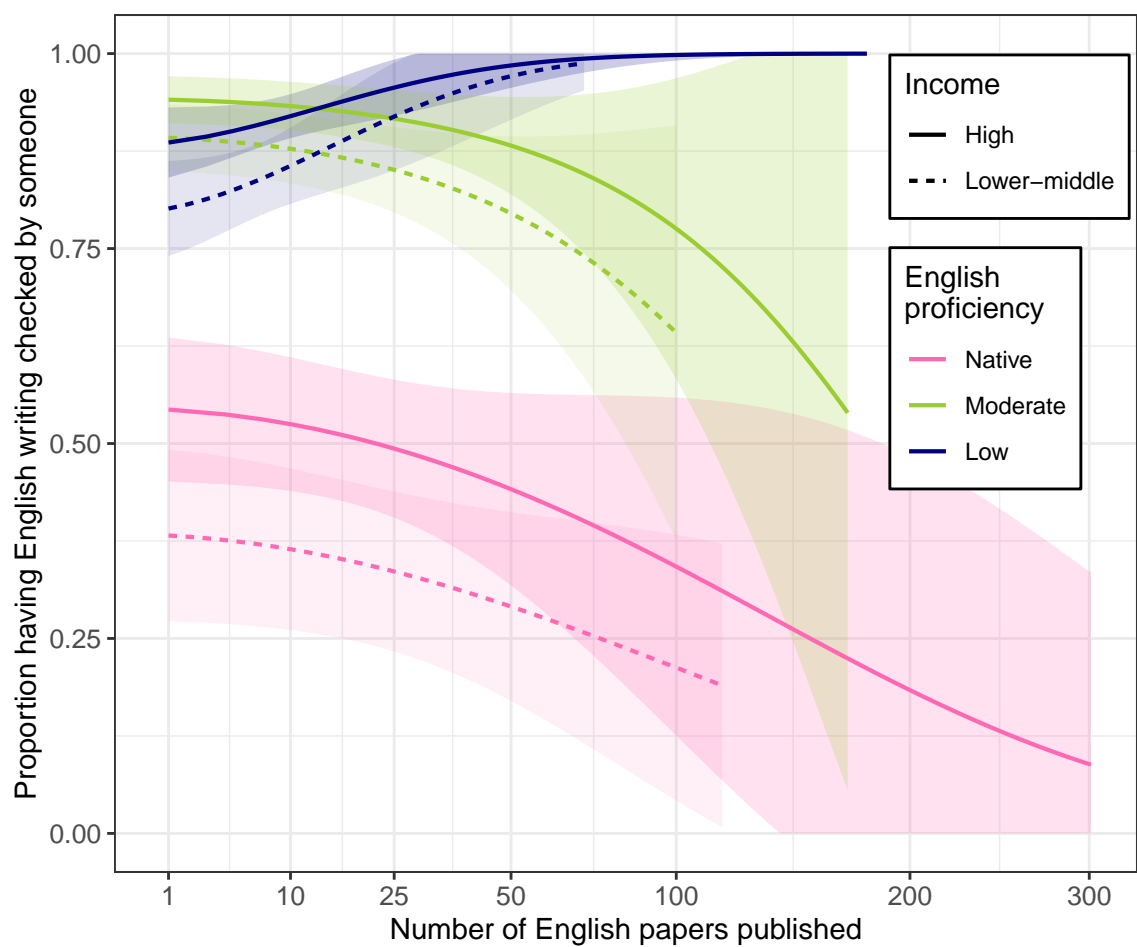

Supplement: S3 Fig — The regression lines (with 95% confidence intervals as shaded areas) represent the estimated relationship with the number of English-language papers published, based on the results shown in S6 Table. The data underlying this figure are raw data directly from the survey questions, which our ethics approval prevents us from sharing to secure confidentiality of the respondents. (PDF) [file pbio.3002184.s020.pdf]

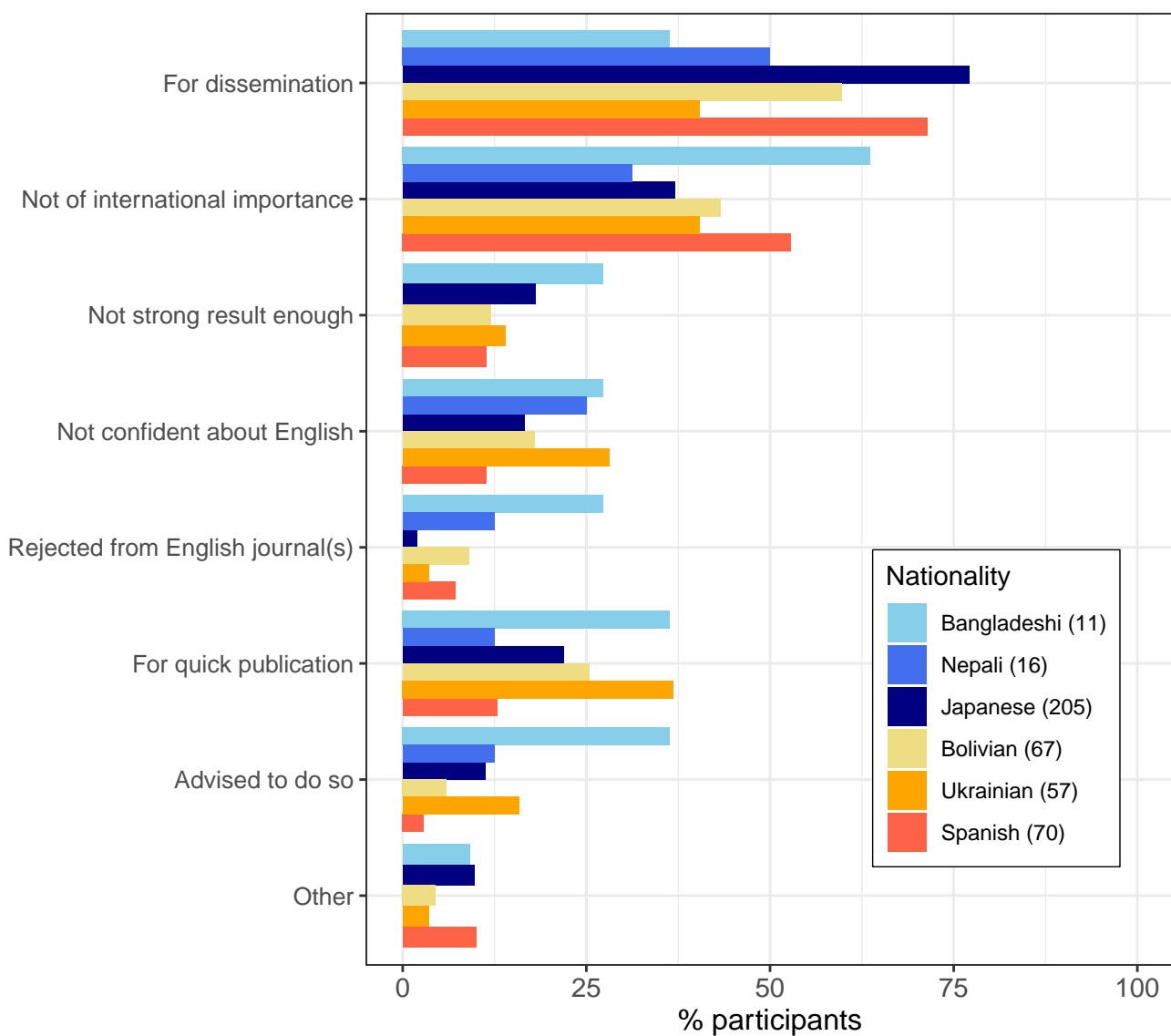

Supplement: S4 Fig — Participants were allowed to choose multiple reasons, and the x-axis indicates the percentage of participants who selected each reason. The data underlying this figure can be found in S1 Data. (PDF) [file pbio.3002184.s021.pdf]

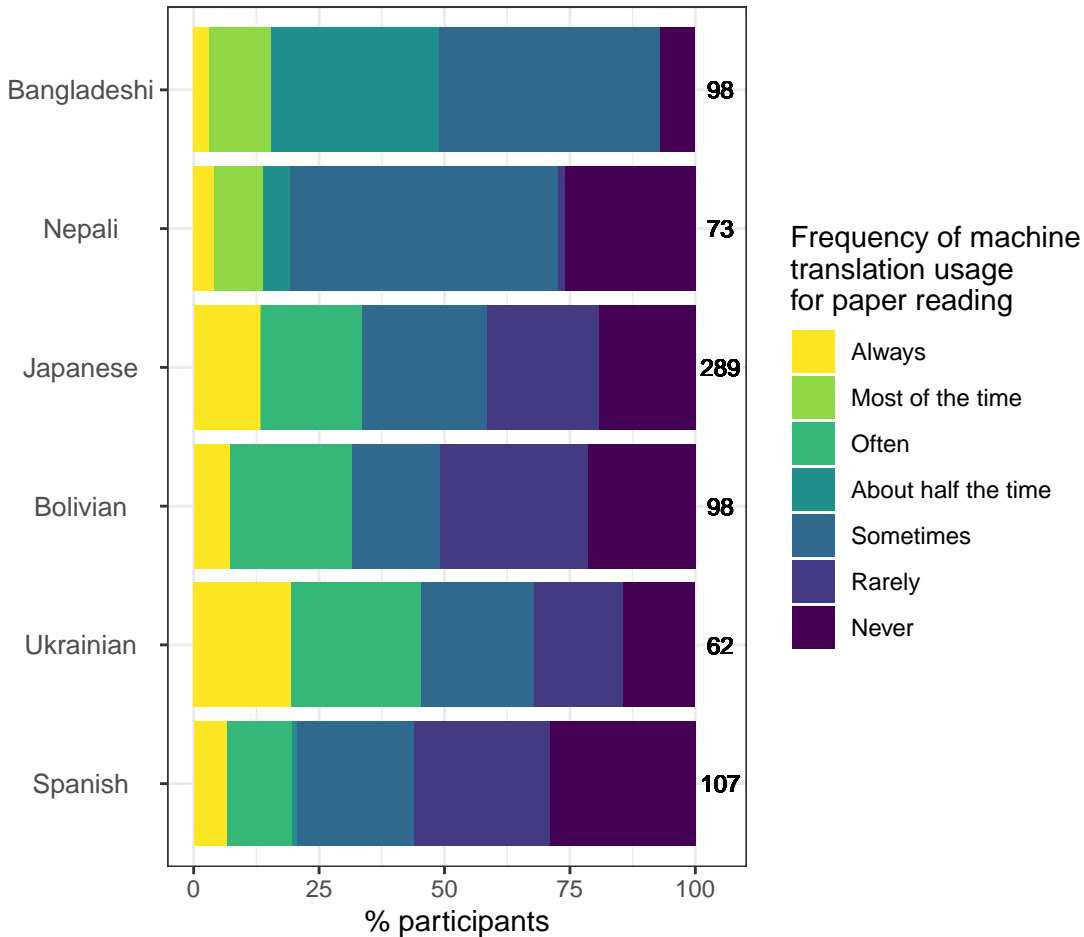

Supplement: S6 Fig — The data underlying this figure can be found in S1 Data. (PDF) [file pbio.3002184.s023.pdf]

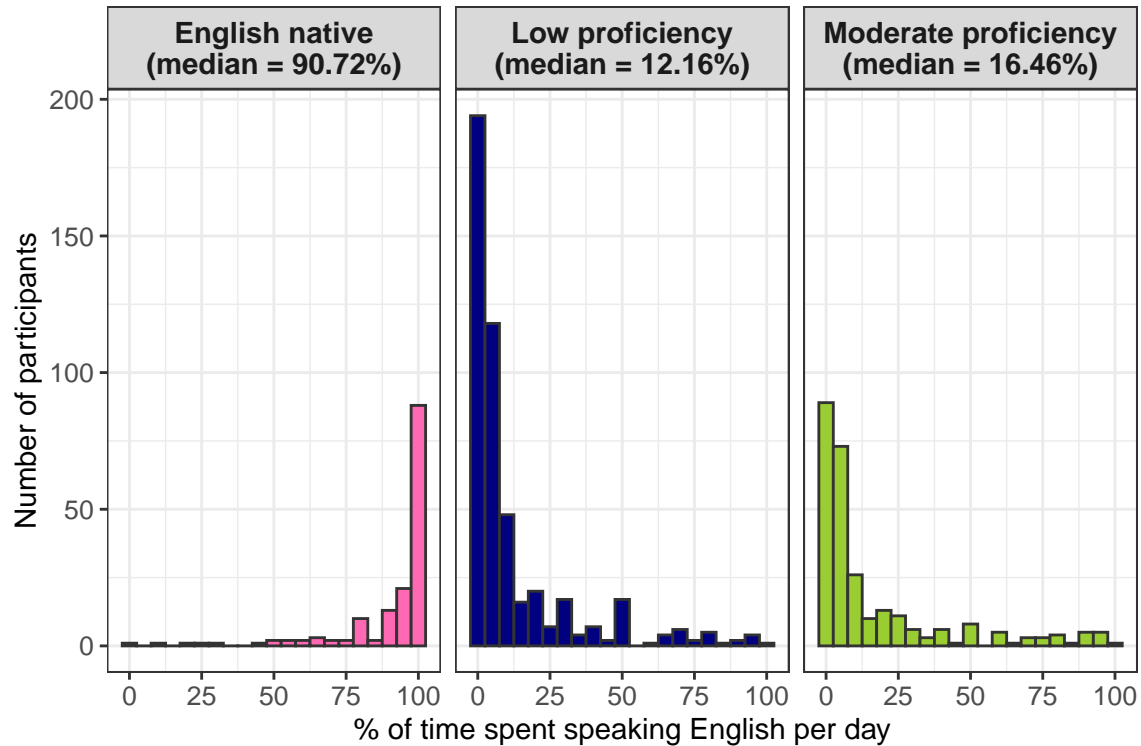

Supplement: S7 Fig — Researchers of moderate English proficiency nationalities speak English in daily life significantly more than those with low English proficiency (generalised linear model with a binomial distribution: Coefficient = 0.35, Standard Error = 0.022, z = 16.40, p < 2.0 × 10^−16^). The data underlying this figure can be found in S1 Data. (PDF) [file pbio.3002184.s024.pdf]

**Low proficiency  
(median = 7.75 years)**

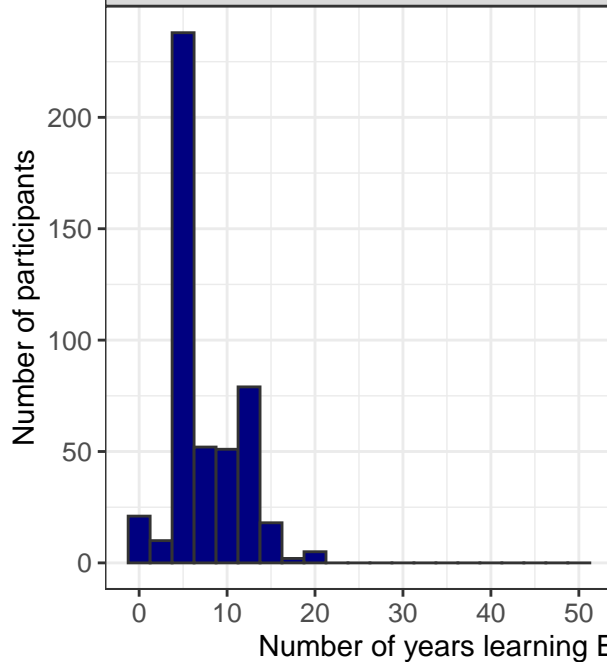

**Moderate proficiency  
(median = 6.24 years)**

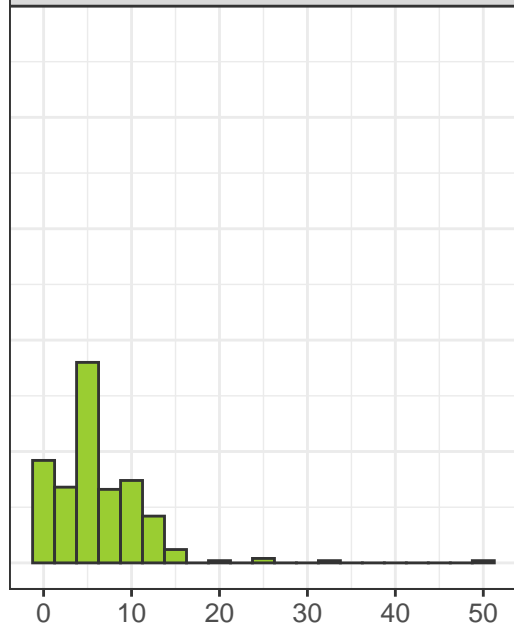

Supplement: S8 Fig — Researchers of moderate English proficiency nationalities have been spending a significantly fewer number of years learning English than those of low English proficiency nationalities (generalised linear model with a negative binomial distribution: Coefficient = −0.22, Standard Error = 0.044, z = −4.96, p = 7.23 × 10^−7^). The data underlying this figure can be found in S1 Data. (PDF) [file pbio.3002184.s025.pdf]

**Low proficiency  
(median = 1.08 years)**

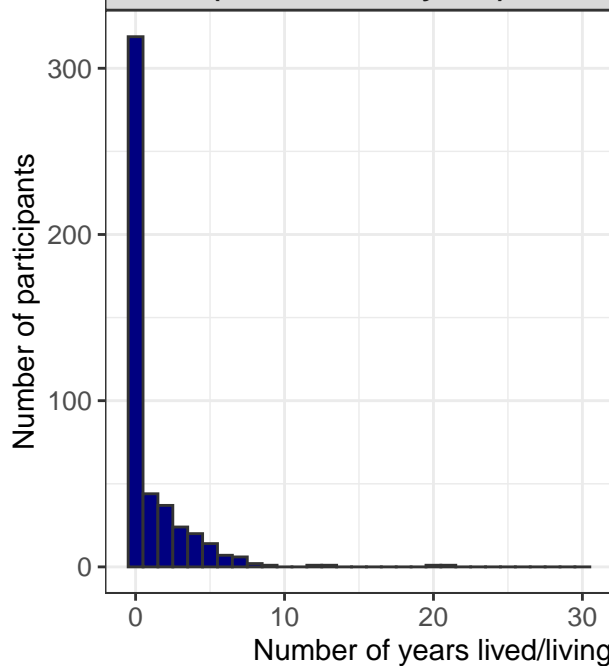

**Moderate proficiency  
(median = 1.72 years)**

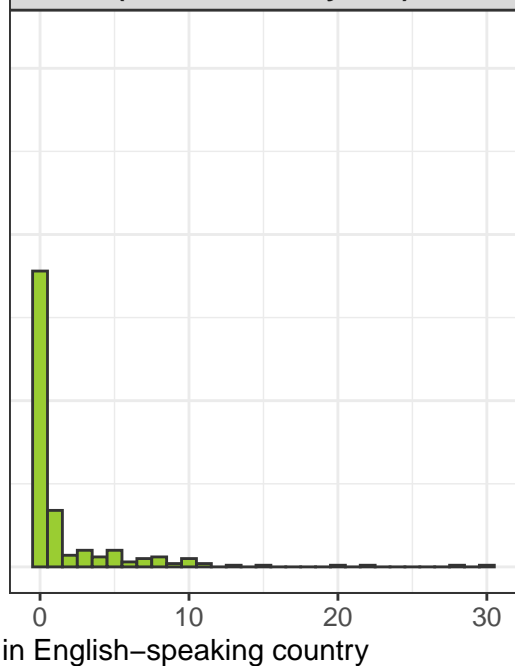

Supplement: S9 Fig — Researchers of moderate English proficiency nationalities have lived in a country where English is the first language significantly longer than those of low English proficiency nationalities (generalised linear model with a negative binomial distribution: Coefficient = 0.47, Standard Error = 0.17, z = 2.69, p = 0.0072). The data underlying this figure can be found in S1 Data. (PDF) [file pbio.3002184.s026.pdf]
