# Supplementary material for: The manifold costs of being a non-native English speaker in science
Source: PLoS Biol. 2023 Jul 18;21(7):e3002184. doi: 10.1371/journal.pbio.3002184 (PMC10353817; doi:10.1371/journal.pbio.3002184)
Supplement: S5 Fig — The estimations are based on the results of the regression shown in S15 Table. The data underlying this figure can be found in S1 Data. (PDF) [file pbio.3002184.s022.pdf]

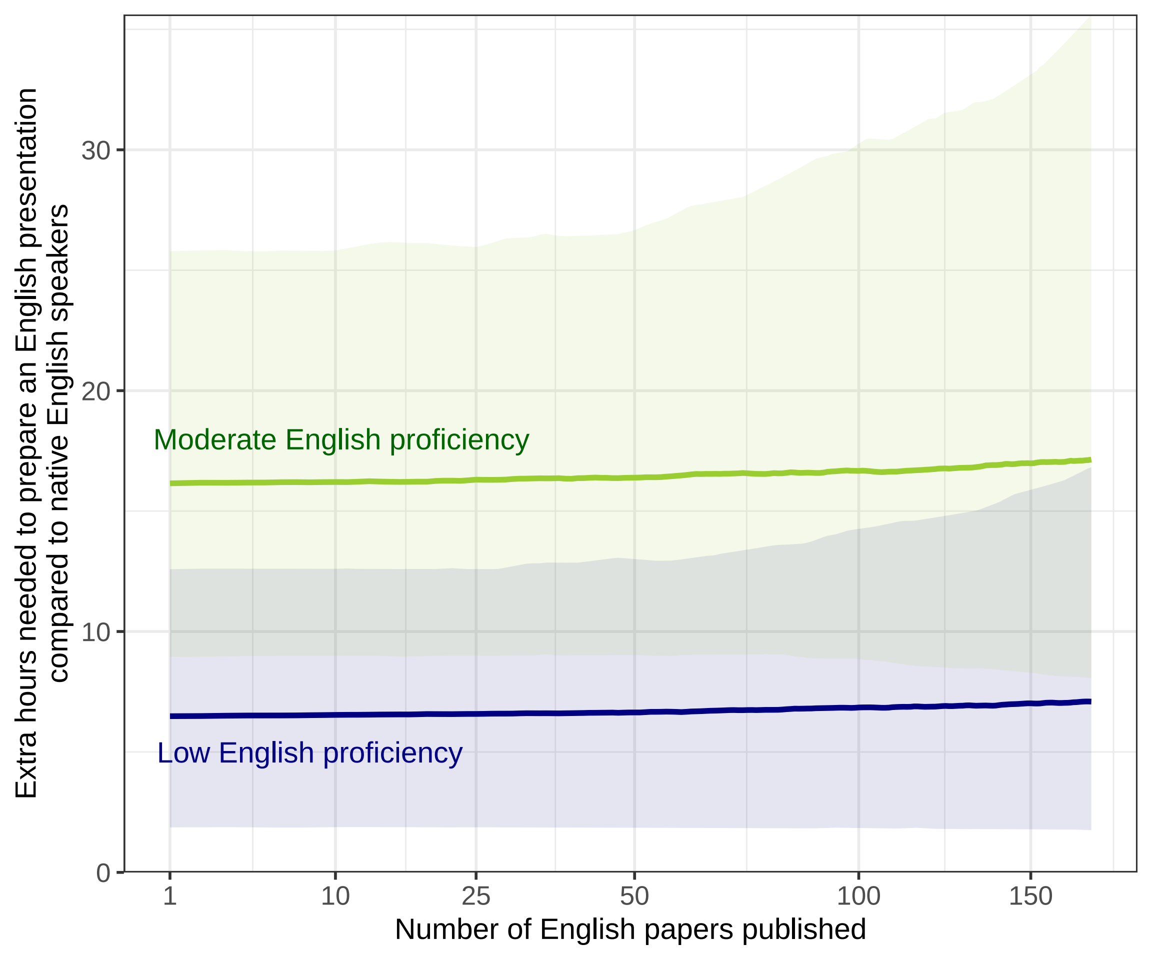

Extra hours needed to prepare an English presentation compared to native English speakers
30
20
10
0
Moderate English proficiency
Low English proficiency
1
10
25
50
100
150
Number of English papers published
