# Supplementary material for: The manifold costs of being a non-native English speaker in science
Source: PLoS Biol. 2023 Jul 18;21(7):e3002184. doi: 10.1371/journal.pbio.3002184 (PMC10353817; doi:10.1371/journal.pbio.3002184)
Supplement: S1 Text — (DOCX) [file pbio.3002184.s028.docx]

**S1 Text.** Questionnaire survey on consequences of language barriers for non-native English speakers for developing career in science.

**Survey on the cost of being a non-native English speaker in science**
Potential participants are kindly asked to read the participant information sheet below before deciding whether or not to participate in this survey.

**Participant information sheet**
**Background**

Being a non-native-English speaker could pose multiple disadvantages when pursuing a career in scientific research, where English is widely recognised as a common language for communication. However, few attempts have been made to quantify the actual cost of being a non-native English speaker in scientific research globally. This has impeded our understanding of language barriers in scientific career developments, potentially leading to the lack of concerted efforts to tackle this issue.

**Aim**

This survey aims to understand the cost of being a non-native-English speaker in sciences and is targeted at anyone with an eligible nationality (i.e., see list of eligible nationalities below) at any career level and of any profession who has published at least one first-authored peer-reviewed English-language paper on ecology, evolutionary biology, conservation biology or related disciplines. The survey will collect information on, for example, the amount of time needed to write a paper or prepare a presentation in English, which will be compared (i) between non-native and native English speakers, and (ii) between countries with different income levels.

Eligible nationalities are Bangla, Bolivian, Japanese, Nepali, Nigerian, Ukrainian, Spanish, United Kingdom (i.e., British, English, Northern Irish, Scottish or Welsh).

**What is involved?**

Participation in this study is entirely online and will take approximately 20 minutes and the survey can be undertaken at a time and place that is convenient to you.

**Participation and withdrawal**

Participation in this study is completely voluntary and you are free to withdraw from this study at any time. If you wish to withdraw, simply stop by closing your internet browser and no data will be saved. Feel free to ask any questions about the research (contact the project or relevant country coordinator).

**Risks**

Participation in this study should involve no physical or mental discomfort and no risks beyond those of everyday living. If, however, you should find any question to be offensive, you are free to omit answering or participating in the specific question.

**Confidentiality and security of data**

Your responses to the survey are anonymous; no identifying information will be collected. All other data will be stored on password-protected computers and only members of the research team will have access to the data. Because all data is non-identifiable, it cannot be linked to individual participants and data will only be presented as summaries of overall responses. The data you provide will only be used for the specific research purposes of this study.

**Benefits of your participation in the study**

The data from the survey will shed light on the consequences of language barriers to scientific career developments of non-native English speakers, which will then be used for raising awareness about the issue among scientific communities and making concerted efforts to reduce language barriers to non-native-English speaking scientists.

**Ethics clearance and contacts**

This study has been cleared in accordance with the ethical review guidelines and processes of the University of Queensland. These guidelines are endorsed by the University’s Human Ethics Committee and registered with the Australian Health Ethics Committee as complying with the National Statement. You are free to discuss your participation in this study with project staff. If you would like to speak to an officer of the University not involved in the study, you may contact the University of Queensland Ethics Officer on +61 3365 3924.

**Study results and further information**

The results will be published online in scientific journals and shared at conferences. You will not be identifiable in any outputs as your data will only be included in an anonymous and aggregated form. If you would like to learn the outcome of the study, please feel free to email the project staff (see below) and we can organise to send you a summary of the study once it is complete. You can also obtain general information on the project at: https://translatesciences.com/.

Thank you for your participation in this study.

Dr Tatsuya Amano, ARC Future Fellow

School of Biological Sciences, The University of Queensland, Brisbane, Qld 4072, Australia. Email: t.amano@uq.edu.au

Violeta Berdejo-Espinola, Senior Research Technician

School of Biological Sciences, The University of Queensland, Brisbane, Qld 4072, Australia. Email: v.berdejoespinola@uq.edu.au

[Country coordinator details]

**Consent form**By checking the two boxes below, you confirm that you have read and understood the above and give your consent for your response to be used in this study:

- I have read the information provided about the research project and understand the nature of my involvement. I understand any information I provide is completely confidential. I agree to take part and understand I can withdraw at any time.
- I am aged 18 or older.

Language and nationality

1.1 What is your first language? For the purpose of this survey first language(s) are defined as “*the language(s) you learnt to speak at home as a child*”.

- Bangla
- English
- Japanese
- Nepali
- Spanish
- Ukrainian
- Other

Please describe. _____________________________________________________________

1.2 Please state your nationality.

- Bangladesh
- Bolivia
- Japan
- Nigeria
- Nepal
- Ukraine
- Spain
- United Kingdom (i.e., British, English, Northern Irish, Scottish or Welsh)
- Other

Basic information

2.1 How old are you?

▼ 18 ... >80

2.2 Please state your gender identity below.

- Male
- Female
- Prefer to self-describe
- Prefer not to say

Please describe. ______________________________________________________________

2.3 Which of the following disciplines best describes your research area (choose only one)?

- Conservation Biology
- Ecology
- Evolutionary Biology
- Other biological sciences

Please describe. ____________________________________________________________

- Sciences other than biological sciences

Please describe. ____________________________________________________________

- Other

Please describe. ____________________________________________________________

2.4 Please state the number of years you have been working in research (including the period of your masters and doctoral degrees, if applicable, but not bachelor’s degree).

▼ 1 ... 80

2.5 What is the percentage of time in a day that you speak in English in your daily life?

▼ 0% ... 100%

2.6 How many years have you learnt English as a foreign language (before starting undergraduate education)?

▼ 0 ... 80

2.7 How many years have you lived in countries or environments where English is the first language?

▼ 0 ... >50

Paper writing in English

3.1 How many peer-reviewed papers (of any categories, such as original research, reviews, perspectives, etc) have you published as the **first author** in **English**?

▼ 0 ... 300

3.2 How many peer-reviewed papers have you published as the first author in any other languages (i.e., non-English languages)?

▼ 0 ... 300

3.3 How many days did it take for you to finish writing the first full draft of your latest first-authored paper in **English** after obtaining the results of your study (assuming you spend seven hours writing the article each day and excluding time spent for non-writing, such as waiting for coauthors' comments, etc)?

▼ <1 ... 120

3.4 How many days would it take for you to finish writing the same article but in **your first language** after obtaining the results of your study (assuming you spend approximately seven hours writing the article each day and excluding time spent for non-writing, such as waiting for coauthors' comments, etc)?

▼ <1 ... 120

3.5 Have you ever asked someone (including your coauthor(s)) to improve the quality of your English writing (e.g., for correcting grammar) as a favour or using a professional service?

- Yes
- No

3.6 What is the percentage of your first-authored papers where you have asked someone (including your coauthor(s)) for a favour to improve the quality of your English writing (e.g., for correcting grammar)?

▼ 1 ... 100%

3.7 What is the percentage of your first-authored papers where you or your coauthor(s) have paid for a professional service to improve the quality of your English writing (e.g., for correcting grammar)?

▼ 1 ... 100%

Publications in English

4.1 Have you ever experienced the rejection of your first-authored paper from any English-language journal where at least one of the reasons for the rejection was your English writing?

- Yes
- No

4.2 How often have you been requested to improve your English writing (e.g., requested to use an English editing service, or ask your colleague to do English editing, etc) in the revision of your first-authored paper in any English-language journal?

▼ Always ... Never

4.3 If you have ever submitted your paper(s) to any journal(s) published in a non-English language(s), what was the reason for you to choose the language(s) for publishing your paper(s)? Please select the reasons below (you can select multiple reasons).

- The topic of the paper is not of international importance (e.g., specific to your country).
- The result was not strong enough to be published in an English-language journal (e.g., the result was not statistically significant).
- It was rejected from English-language journal(s).
- You were not confident enough about your English writing.
- You wanted to publish it as soon as possible.
- You wanted to disseminate the result to speakers of the language(s), such as local researchers, the general public and/or policymakers in your country.
- Your co-author(s) or supervisor(s) advised you to do so.
- Other.

Please describe. ______________________________________________________________

- Not applicable.

4.4 Have you ever provided the non-English-language abstract of your English-language paper(s)?

- Yes
- No

4.5 Have you ever conducted outreach activities (e.g., publishing a press release or writing a blog post) in English AND any other language(s) to disseminate your English-language paper(s)?

- Yes
- No

Paper reading in English

5.1 How many minutes did it take for you to fully read and understand the last **English-language** original article you read in your field (e.g., ecology)?

▼ 1 ... 180

5.2 How many minutes do you think it would take for you to read and fully understand the same English-language paper if you could read it in your first language?

▼ 1 ... 180

5.3 How often do you use machine translation (e.g., Google Translate) when reading English-language papers?

▼ Always ... Never

Conferences in English

6.1 Have you attended a conference where English is the primary language?

- Yes
- No

6.2 How often have you decided not to attend an English-language conference (either for presenting your research or just for participating) because you were not confident enough to communicate in English?

▼ Always ... Never

6.3 If you have ever attended an English-language conference, how often have you decided to present your research as a poster presentation, instead of an oral presentation, at an English-language conference because you were not confident enough to do an oral presentation in English?

▼ Always ... Never

6.4 If you have ever given an oral presentation in English, how many hours did it take for you to prepare and practice the last oral presentation in **English**?

▼ <1 ... 100

6.5 How many hours would it take for you to prepare and practice the same presentation but in **your first language**?

▼ <1 ... 100

6.6 If you have ever presented your research in English, how often have you experienced a situation where you could not explain your research confidently during your presentation (including Q & A sessions) due to language barriers (e.g., because you are not confident about communication in English)?

▼ Always ... Never

Closing section

7.1 Do you have any comments about language barriers for non-native English speakers in academia?

________________________________________________________________

7.2 Please provide any feedback about this survey here.

________________________________________________________________

Thank you. Please submit your responses by clicking on the right arrow below
Please visit our [website](https://translatesciences.com/) to see more of what we do.

Dr Tatsuya Amano, ARC Future Fellow. Email: t.amano@uq.edu.au

Violeta Berdejo-Espinola, Senior Research Technician. Email: v.berdejoespinola@uq.edu.au

[Country coordinator details]
