# Supplementary material for: The manifold costs of being a non-native English speaker in science
Source: PLoS Biol. 2023 Jul 18;21(7):e3002184. doi: 10.1371/journal.pbio.3002184 (PMC10353817; doi:10.1371/journal.pbio.3002184)
Supplement: S2 Text — (DOCX) [file pbio.3002184.s029.docx]

**科学において英語を第一言語としない人が直面する様々なコスト**

**要旨**

科学の共通言語として英語が用いられていることで、英語を第一言語としない研究者の科学への貢献が大きく阻害されている。しかし、英語を第一言語としない研究者のキャリア形成における言葉の壁の影響を定量化した研究はほとんどない。そこで本研究では、環境科学分野の研究者908名を対象とした調査を行い、異なる言語的・経済的背景を持つ研究者間で、英語で科学を行うために必要な努力量を推定・比較した。この調査から、英語を第一言語としない研究者は、特にキャリアの初期において、英語での論文の読解や執筆、研究発表の準備から、多言語での普及活動に至るまで、様々な科学活動を行う上で、英語を第一言語とする研究者よりも多くの労力を費やしていることが明らかになった。また、言葉の壁が原因で、英語で行われる国際会議に出席できなかったり、口頭発表ができなかったりすることも明らかになった。英語を第一言語としない研究者の潜在能力を最大限に引き出すために、学術界はこの問題についての認識を高め、解決に取り組んでいく必要があるだろう。また本稿では、機関や学術誌、研究資金の提供者、学会などがすぐに実施できる解決策も提案している。


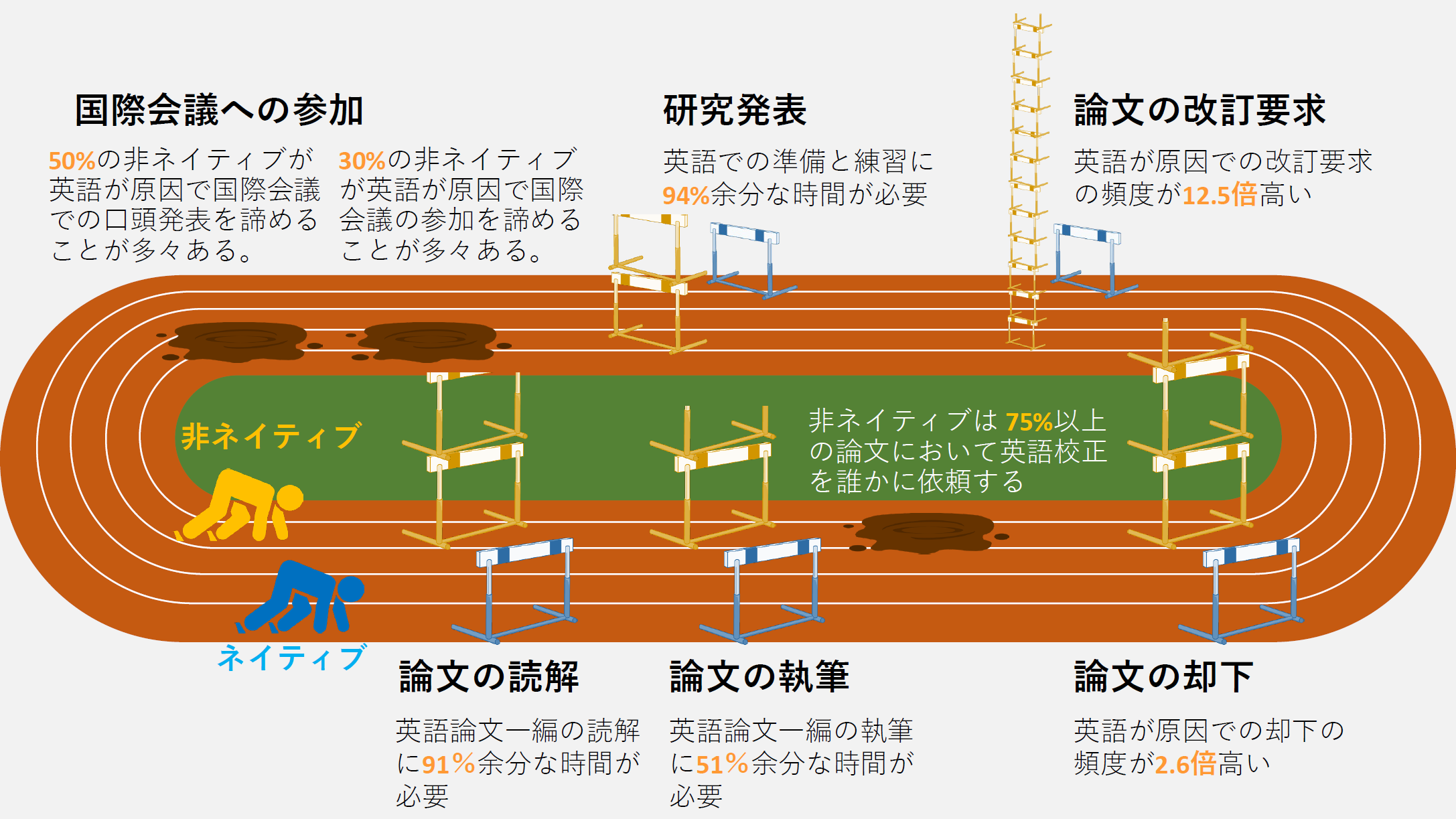


**図５．本研究で推定された科学活動を行う際に英語を第一言語としない人が被る不利益。**ハードルの高さは、英語を第一言語としない人（非ネイティブ）にとって、英語での科学論文の読解、執筆、口頭での研究発表の準備と練習にかかる時間と、執筆した論文の英語が原因で学術誌に却下、改訂要求される頻度を、英語を第一言語とする人（ネイティブ）に対する相対値で示している。値は英語論文を一編出版している人の推定値で、非ネイティブの値は英語レベルが中程度と低い国籍のうち、高い方の値を示している。本図は科学の本質が競争であることを示唆するためのものではない。


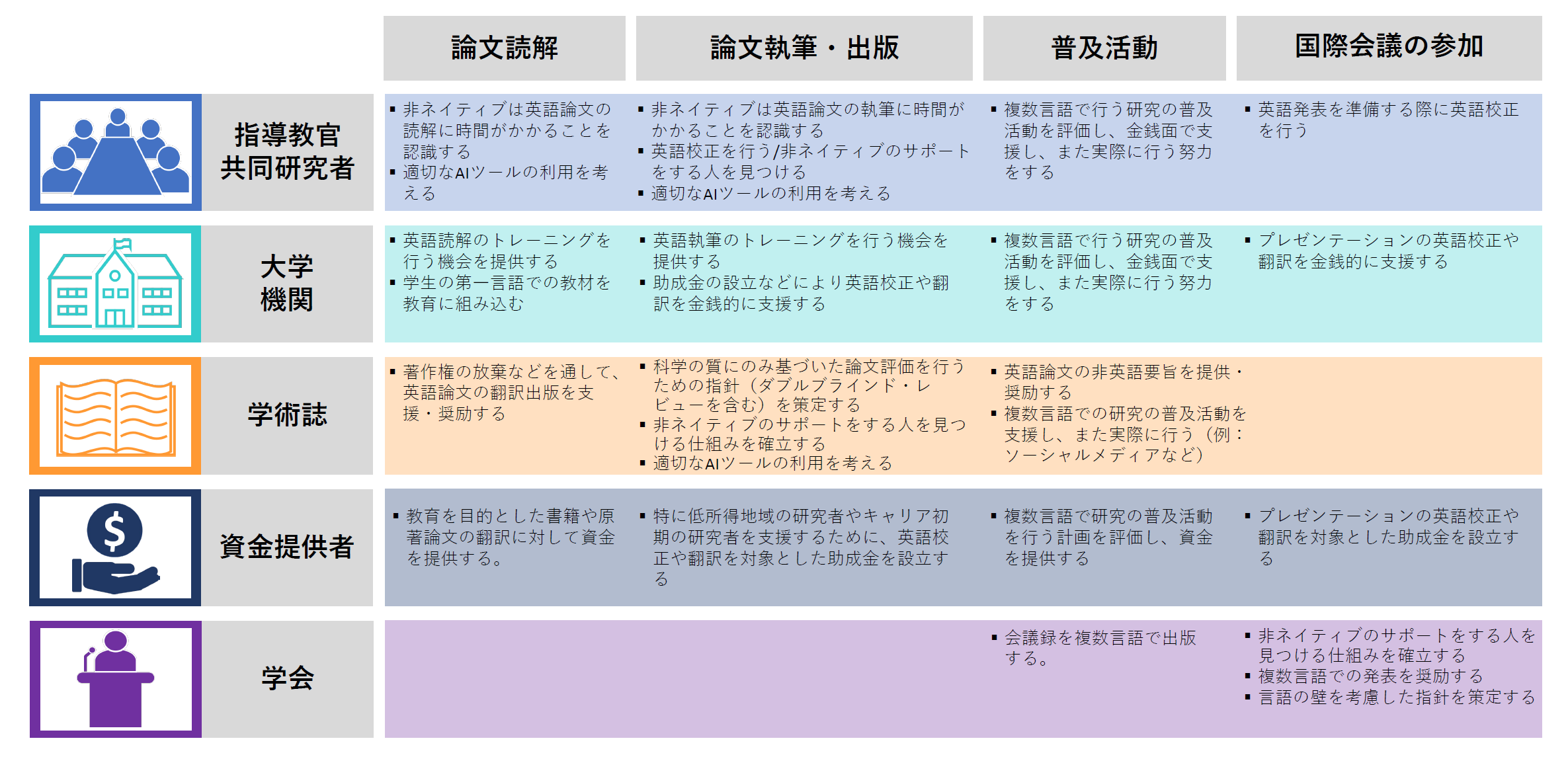


**図６．英語を第一言語としない人が各科学活動において被る不利益を軽減するための解決策の例。**AI：人工知能。また他の解決策については引用文献 [35, 38, 39] も参照。
