# Supplementary material for: The manifold costs of being a non-native English speaker in science
Source: PLoS Biol. 2023 Jul 18;21(7):e3002184. doi: 10.1371/journal.pbio.3002184 (PMC10353817; doi:10.1371/journal.pbio.3002184)
Supplement: S3 Text — (DOCX) [file pbio.3002184.s030.docx]

**गैर-अंग्रेजी भाषीले विज्ञानमा चुकाउनुपरेको अनेकाै‌ मूल्यहरु**

सारा‌ंश

विज्ञानमा साझा भाषाको रूपमा अंग्रेजीको प्रयोग हुनाले गैर-अंग्रेजीभाषीहरुले विज्ञानमा पुर्याउने योगदानलाई बृहत बनाउन ठूलो अवरोध खडा गरेको छ। यद्यपि केही अध्ययनहरूले भाषीक अवरोधको कारण गैर-अंग्रेजीभाषी अनुसन्धानकर्ताहरुको बृतिविकासमा परेकाे प्रभावकाे परिमाणात्मक लेखाजाेखा गरेका छन्। वातावरणीय विज्ञानका ९०८ शोधकर्ताहरूको सर्वेक्षण गरेर, यस अध्ययनले विभिन्न देश र विभिन्न भाषिक तथा आर्थिक पृष्ठभूमि भएका शोधकर्ताहरू बिच अङ्ग्रेजीमा वैज्ञानिक गतिविधिहरू सञ्चालन गर्न आवश्यक पर्ने मेहनतको अनुमान र तुलना गरेकाे छ । अंग्रेजीभाषीहरु भन्दा गैर-अंग्रेजीभाषीहरु विशेष गरी आफ्नो करियरको सुरुवातमा भएकाहरुले वैज्ञानिक गतिविधिहरू सञ्चालन गर्न, अनुसन्धनात्मक लेखहरू पढ्न, लेख्न, अंग्रेजीमा प्रस्तुतीकरणहरू तयार गर्नेदेखि लिएर, विभिन्न भाषाहरूमा सोध प्रकाशित गर्न तुलनात्मक रुपमा बढी मेहनत गर्ने गरेकाे हाम्रो सर्वेक्षणले देखाएकाे छ। भाषीक अवरोधले उनीहरूलाई अङ्ग्रेजीमा आयोजित अन्तर्राष्ट्रिय सम्मेलनहरूमा भाग नलिन वा मौखिक प्रस्तुति नदिन प्रेरित गर्ने गरेकाे पनि हुन सक्छ। वैज्ञानिक समुदायहरूलाई यी बेफाइदाहरू पहिचान गर्न र विज्ञानमा गैर-अङ्ग्रेजीभाषीहरुको अमुल्य सम्भावनालाई अंगाल्नका लागि हामी आग्रह गर्दछौं। यस अध्ययनले सम्भावित समाधानहरू पनि प्रस्ताव गर्दछ जसलाइ व्यक्ति, संस्था, जर्नल, दाता तथा लगानीकर्ता, र सम्मेलनहरूले तत्काल लागू गर्न सक्दछन्।


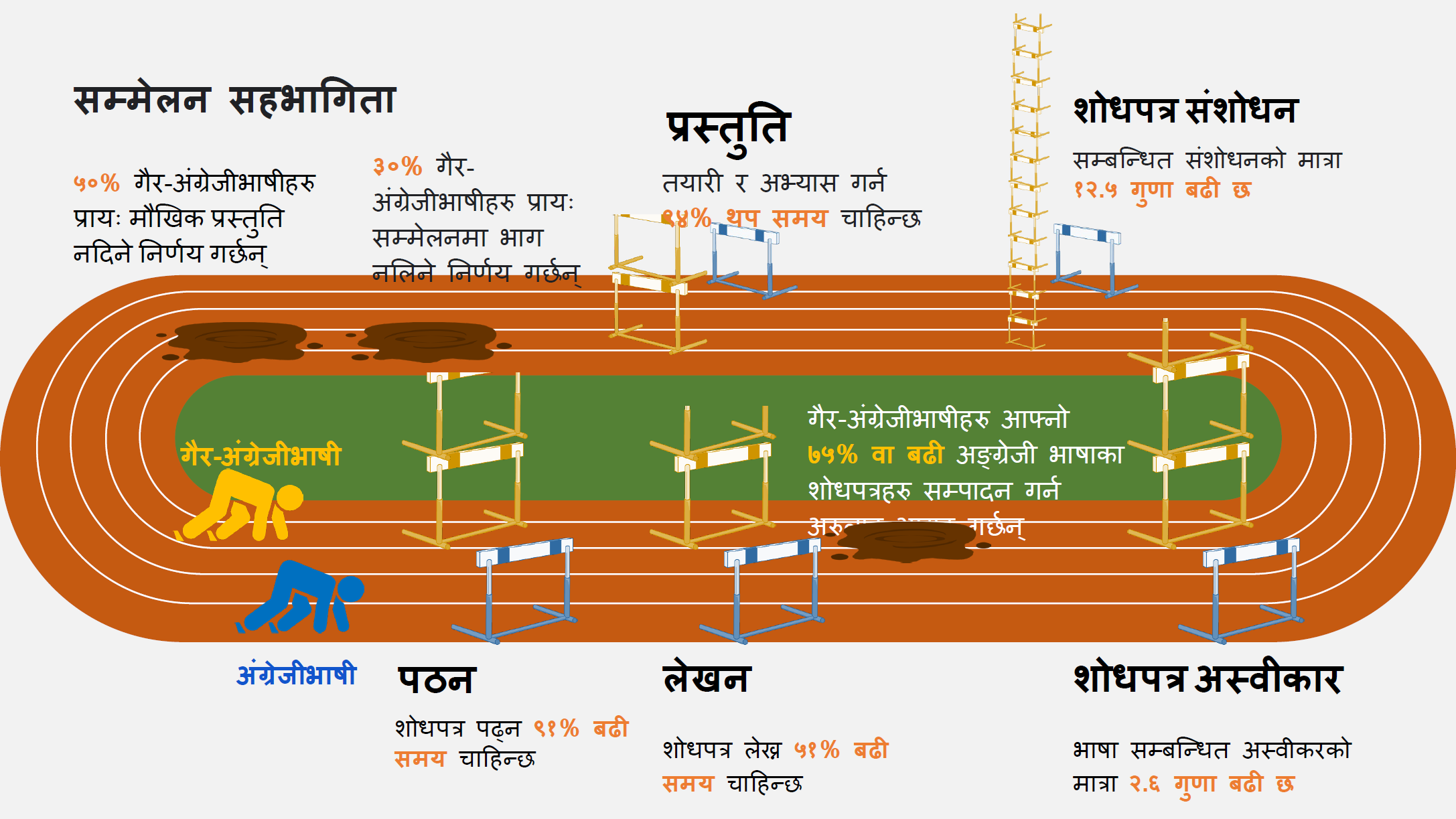


**चित्र ५.** **विभिन्न वैज्ञानिक गतिविधिहरू सञ्चालन गर्दा गैर-अंग्रेजीभाषीहरुका लागि अनुमानित बेफाईदाहरु।** चित्रमा देखाइएका विभिन्न अड्चनहरूका उचाइले अंग्रेजी-भाषाको शोधपत्र पढ्न (पढन), अंग्रेजीमा शोधपत्र लेख्न (लेखन), र अंग्रेजीमा मौखिक प्रस्तुतीकरण (प्रस्तुति) तयार गर्न सापेक्ष रुपमा लाग्ने धेरै समय देखाउँछ र भाषाकै कारण अंग्रेजी भाषाको शोधपत्र अंग्रेजीभाषीहरुको तुलनामा गैर-अंग्रेजीभाषीहरुको अस्वीकृत वा परिमार्जन बढिमात्रमा हुन्छ ।

प्रस्तुत अ‌ंकहरु अंग्रेजीभाषीको अ‌ंकहरुको तुलनामा गैर-अंग्रेजीभाषीहरुका लागि हुन् जसले केवल एउटा मात्र अंग्रेजी-भाषाको शोधपत्र प्रकाशित गरेका छन् (उच्च अंकहरु मध्यम र निम्न अंग्रेजी कुशलता भएका मुलुकहरुबाट हुन्)। यो चित्रले विज्ञान एक दौड हो भनेर सुझाउन खाेजेकाे हाेइन।


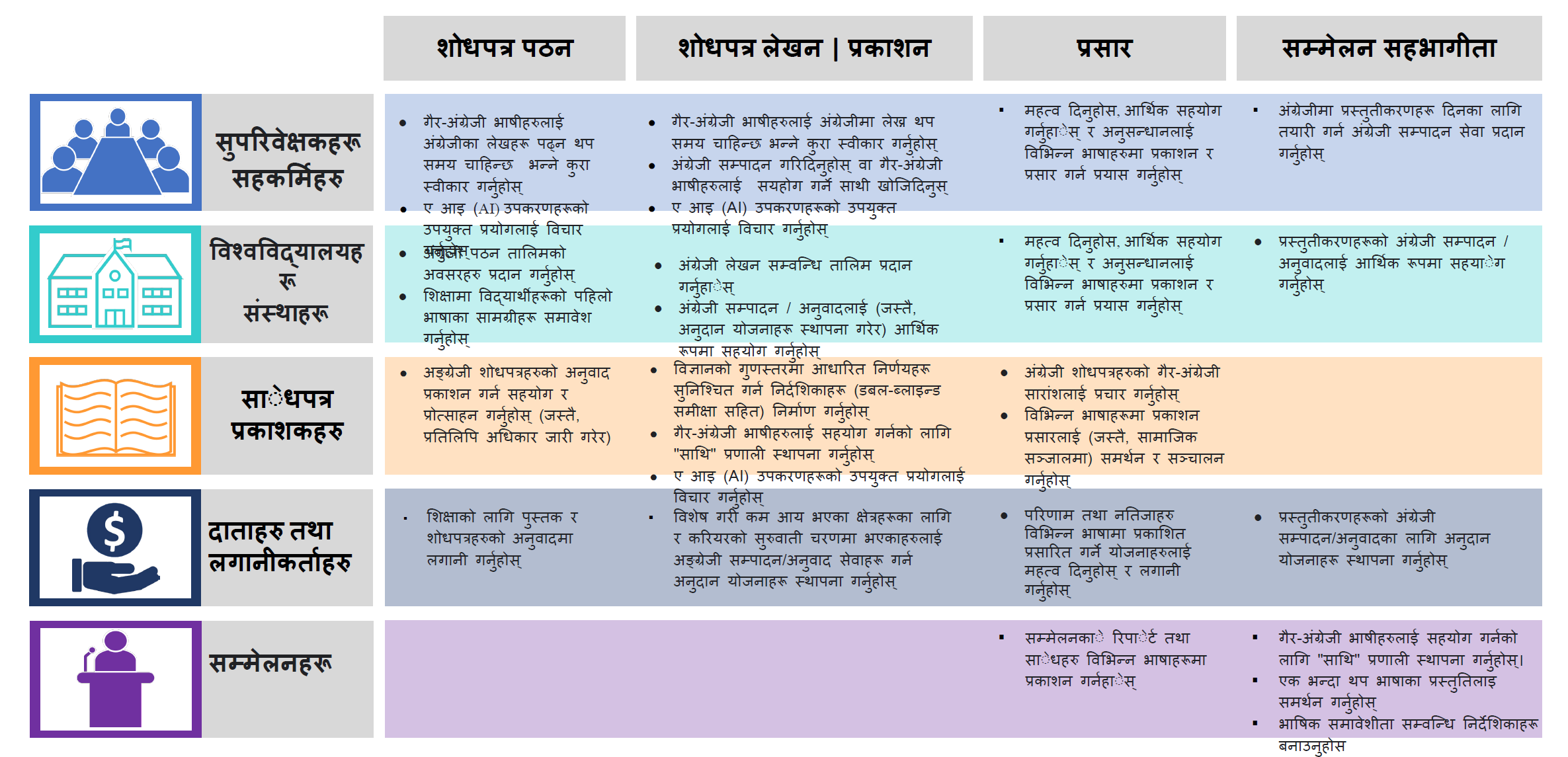


**चित्र 6. हरेक प्रकारको वैज्ञानिक गतिविधिहरूमा गैर-अंग्रेजीभाषीहरुका लागि हुने बेफाइदाहरू कम गर्न सम्भावित समाधानको उदाहरणहरू।** AI: कृत्रिम बुद्धिमत्ता। अन्य सम्भावित समाधानहरूको लागि [३५, ३८, ३९] पनि हेर्नुहोस्।
