# Supplementary material for: The manifold costs of being a non-native English speaker in science
Source: PLoS Biol. 2023 Jul 18;21(7):e3002184. doi: 10.1371/journal.pbio.3002184 (PMC10353817; doi:10.1371/journal.pbio.3002184)
Supplement: S4 Text — (DOCX) [file pbio.3002184.s031.docx]

**Los múltiples costos de ser un angloparlante no nativo en la ciencia**

**Resumen**

El inglés como lengua franca de la ciencia representa un gran obstáculo para la participación de personas que no son hablantes nativos de este idioma. Aun así, no existe en la actualidad una estimación de los costos que imponen las barreras impuestas por el inglés en el desarrollo profesional de investigadores e investigadoras. En este estudio, se estimó, mediante una encuesta a 908 investigadores en ciencias ambientales, la cantidad de esfuerzo requerido para desarrollar actividades científicas en inglés. Luego se compararon los resultados entre investigadores e investigadoras de diferentes países y, por lo tanto, de diferentes antecedentes lingüísticos y económicos, incluyendo participantes angloparlantes nativos. Los resultados de la encuesta revelan que, sin importar el país, los y las angloparlantes no nativos dedican más esfuerzo que los y las angloparlantes nativos en la realización de actividades científicas que incluyen leer y escribir artículos, y preparar presentaciones o pósters para difundir su investigación en congresos. Esta brecha, que es especialmente amplía al comienzo de la carrera científica, puede llevar a que investigadores e investigadoras desistan de participar en conferencias internacionales realizadas en inglés. Debido a que estas brechas pueden impedir la difusión internacional del conocimiento, invitamos a las comunidades científicas a reconocer y abordar estas desventajas para liberar el potencial no aprovechado de los angloparlantes no nativos. Individuos, instituciones, revistas, financiadores y conferencias científicas pueden contribuir a la solución de esta problemática global.

Liberar el potencial de las comunidades marginalizadas es uno de los retos urgentes de la ciencia. La colaboración entre un grupo diverso de personas puede contribuir ideas para la resolución de problemáticas [1] y generar mayores niveles de innovación científica [2] e impacto en la sociedad [3]. Hoy en día se reconoce cada vez más la necesidad de promover la participación de una mayor diversidad de personas que permita ampliar puntos de vista, sistemas de conocimiento y soluciones para abordar con éxito los retos mundiales, como las crisis de la biodiversidad y el clima [4-6], desde una perspectiva multidisciplinar [7-9].

Para aumentar la diversidad dentro de las comunidades científicas es necesario derribar las barreras que impiden el desarrollo profesional de los grupos de investigadores marginalizados, y una de esas barreras tiene su origen en el idioma. Aunque el uso del inglés como lengua común de la ciencia ha contribuido sin duda al avance de la ciencia [10], este beneficio tiene un costo considerable para aquellos cuya lengua materna no es el inglés (en lo sucesivo, angloparlantes no nativos). Las y los angloparlantes no nativos, que constituyen la mayoría de la población mundial, se enfrentan a una serie de retos a la hora de realizar y comunicar trabajos científicos en inglés, que inevitablemente suponen una carga excesiva para el desarrollo de su carrera científica. Esta es una problemática ampliamente reconocida [11, 12], ya que el inglés desempeña actualmente un papel dominante en la ejecución y comunicación de la ciencia, así como en la evaluación de los científicos de casi cualquier disciplina [13]. Aunque organizaciones como las Naciones Unidas para la Educación, la Ciencia y la Cultura (UNESCO) destacan la necesidad de superar las barreras lingüísticas para alcanzar cuatro de los valores fundamentales y principios rectores de la ciencia abierta (Equidad e imparcialidad, Diversidad e inclusión, Igualdad de oportunidades y Colaboración, participación e inclusión) [14], la realidad es que las comunidades científicas siguen careciendo de un esfuerzo concertado necesario para reducir las barreras lingüísticas a las que se enfrentan las y los angloparlantes no nativos y promover la equidad en la ciencia.

En la actualidad, las dificultades a las que se enfrentan las y los angloparlantes no nativos a la hora de hacer ciencia, y los costos que éstas tienen para el desarrollo de su carrera, siguen siendo poco conocidas. Estudios anteriores han informado sobre la experiencia y la percepción de las barreras lingüísticas en hablantes de una única lengua no inglesa [15] o para determinados tipos de actividades científicas, como la redacción de artículos [16], la publicación de artículos [17] y la difusión de la investigación [18]. También han intentado evaluar las desventajas de ser angloparlante no nativos en la ciencia [por ejemplo, 19, 20]. Sin embargo, hasta la fecha, ningún estudio ha cuantificado cómo los múltiples aspectos de las barreras lingüísticas afectan simultáneamente al desarrollo profesional de hablantes de diferentes lenguas no inglesas teniendo como referencia el desempeño de los hablantes nativos de inglés.

Este estudio aborda esta laguna de conocimiento estimando la cantidad de esfuerzo (por ejemplo, tiempo y coste económico) que supone llevar a cabo una serie de actividades científicas en inglés, y comparándolo entre investigadores/as de países con diferentes antecedentes lingüísticos y económicos. Con el objetivo de cuantificar las múltiples desventajas a las que se enfrentan las y los angloparlantes no nativos que se desempeñan en el campo de la ciencia, realizamos una encuesta a un total de 908 investigadores/as de ciencias ambientales (en particular, ecología, biología evolutiva, biología de la conservación y disciplinas afines) que han publicado al menos un artículo revisado por pares en inglés, con una de las ocho nacionalidades siguientes: bangladesí (n = 106), boliviana (100), británica (112), japonesa (294), nepalí (82), nigeriana (40), española (108) y ucraniana (66) (véanse más detalles, incluida su información demográfica, en la Tabla S1). Estas nacionalidades fueron estratificadas según el nivel de dominio del inglés de cada país (basado en el Indice de Proficiencia del Inglés [21]) y la renta (basada en la lista de economías del Banco Mundial [22]): Bangladesh, Nepal (bajo dominio del inglés y renta media-baja), Japón (bajo dominio del inglés y renta alta), Bolivia, Ucrania (dominio moderado del inglés y renta media-baja), España (dominio moderado del inglés y renta alta), Nigeria (inglés como lengua oficial y renta media-baja) e Inglaterra (inglés como lengua oficial y renta alta). Con ello se pretendía distinguir el efecto de las barreras lingüísticas del efecto de otros tipos de barreras en la ciencia que a menudo se confunden con las barreras lingüísticas, en particular las barreras económicas a la participación en conferencias [23, 24]. En la encuesta se preguntó a los participantes sobre la cantidad de esfuerzo necesario para llevar a cabo cinco categorías de actividades científicas: lectura de artículos, redacción, publicación y difusión, y participación en congresos (véanse los Materiales y Métodos para más detalles, y el Texto Suplementario S1 para la encuesta propiamente dicha).

Los resultados revelaron profundas desventajas para las y los angloparlantes no nativos en las cinco categorías de actividades científicas incluidas en la encuesta. En primer lugar, las y los angloparlantes no nativos necesitan más tiempo para leer un artículo en inglés, un requisito para obtener los conocimientos necesarios, especialmente los de vanguardia, en la investigación (Fig. 1A, Tabla S2). En una comparación entre investigadores/as que han publicado sólo un artículo en inglés, angloparlantes no nativos de nacionalidades con un dominio moderado y bajo del inglés emplean respectivamente una mediana del 46,6% (percentiles 2,5 - 97,5: 19,0 - 78,1%) y del 90,8% (60,6 - 125,4%) más de tiempo en leer un artículo en inglés que las y los angloparlantes nativos (Figs. 1A y S1). Esta desventaja se observa incluso en las y los investigadores/as en etapas avanzadas de su carrera científica, especialmente en los de nacionalidades con un bajo nivel de inglés (Figs. 1A y S1). Es importante destacar que, en una comparación del tiempo estimado necesario para leer un artículo escrito en su lengua materna, las y los angloparlantes no nativos necesitaron menos tiempo que los angloparlantes nativos (Fig. 1B, Tabla S3), lo que demuestra que la desventaja mencionada se debe a la necesidad de leer en inglés.

Del mismo modo, las y los angloparlantes no nativos necesitan más tiempo para escribir un artículo en inglés que sus colegas angloparlantes nativos, sobre todo cuando se encuentran en una fase temprana de su carrera (Fig. 1C, Tabla S4). En una comparación de investigadores/as que han publicado sólo un artículo en inglés, angloparlantes no nativos de nacionalidades con un dominio moderado y bajo del inglés emplean respectivamente una mediana del 50,6% (percentiles 2,5 - 97,5: 31,1 - 52,6%) y del 29,8% (6,6 - 59,3%) más de tiempo en escribir un artículo en inglés, que las y los angloparlantes nativos (Figs. 1C y S2). Cabe destacar que esta desventaja no se da en investigadores/as que se encuentran en una etapa posterior de su carrera (Fig. S2). Sin embargo, similar a lo que sucede con la competencia de lectura, las y los angloparlantes no nativos necesitan menos tiempo para escribir un artículo en su lengua materna que las y los angloparlantes nativos (Fig. 1D, Tabla S5). Esto significa que la necesidad de escribir en inglés, y no en sus lenguas maternas, supone una desventaja para las y los angloparlantes no nativos.

Las y los angloparlantes no nativos también requieren un esfuerzo mayor que las y los angloparlantes nativos para la corrección de sus trabajos en inglés. Aparte de investigadores/as senior de nacionalidades con un dominio moderado del inglés, las y los angloparlantes no nativos piden a alguien que corrija su inglés en una media del 75% o más de sus trabajos, mientras que la mayoría de las y los angloparlantes no nativos lo hacen en menos de la mitad de sus trabajos (Fig. S3, Tabla S6). Las y los angloparlantes no nativos de nacionalidades con un dominio moderado del inglés tienden a pedir a alguien que corrija su inglés como un favor (Fig. 1E, Tabla S7), mientras que los de nacionalidad con un dominio bajo del inglés y un nivel socioeconómico alto (es decir, los japoneses en la muestra de nuestro estudio) tienden a utilizar un servicio profesional de corrección de inglés (Fig. 1F, Tabla S8). Las y los angloparlantes no nativos de nacionalidades con bajo dominio del inglés y nivel socioeconómico medio-bajo ni piden a alguien que corrija su inglés como favor ni utilizan un servicio de pago para la mayoría de sus trabajos (Fig. 1E, F).


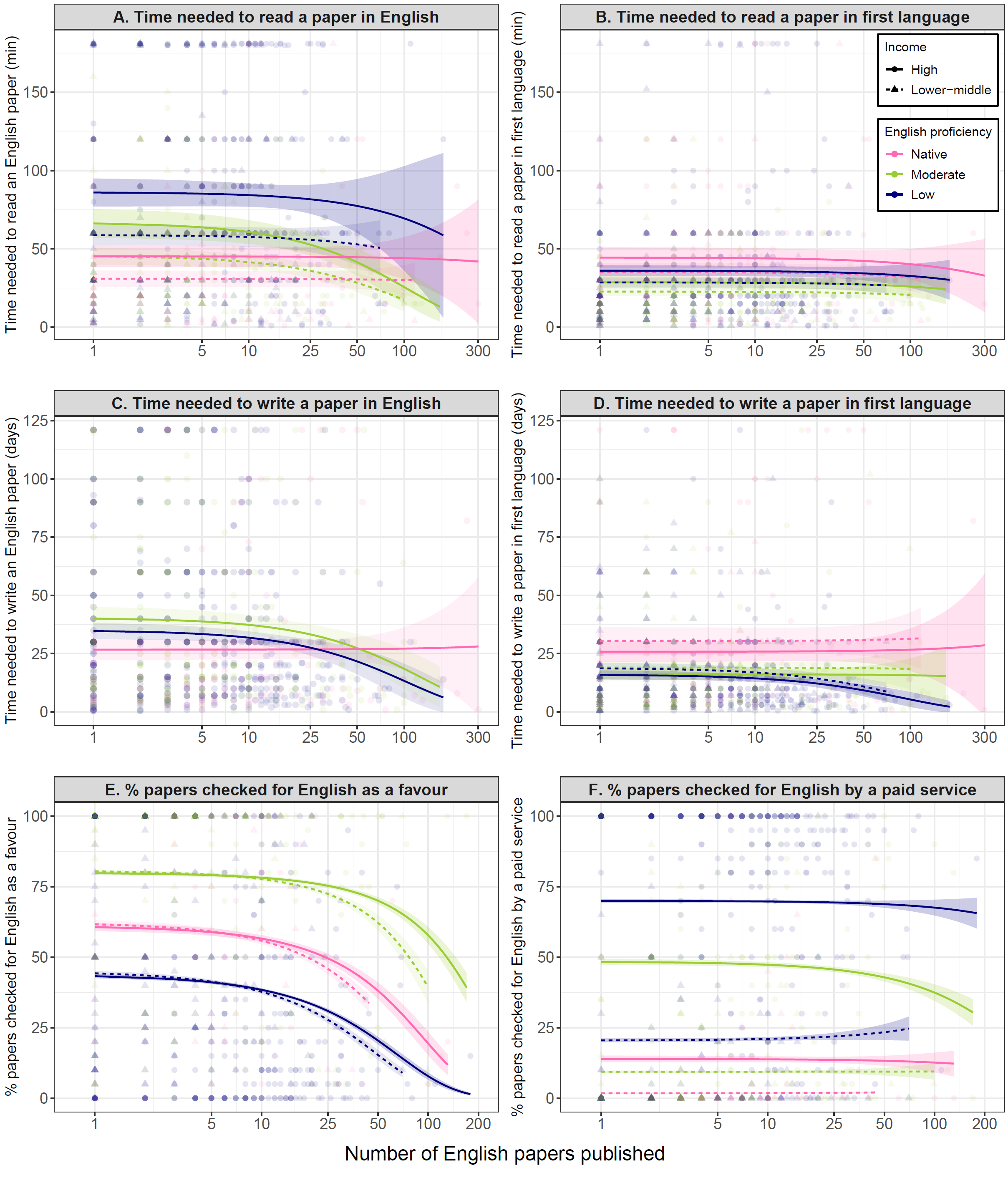


**Fig. 1. Barreras lingüísticas en la lectura y escritura de artículos.** (A) Minutos que se tarda en leer y comprender el contenido del artículo de investigación en inglés más reciente que leyó cada participante en su campo. (B) Minutos que se tardarían en leer y comprender completamente el mismo artículo en la lengua materna de cada uno. (C) Número de días (suponiendo que se dedican siete horas al día) que se tardaría en redactar el primer borrador del último artículo en inglés del que cada participante es autor por primera vez. (D) Número de días que se tardaría en escribir el primer borrador del mismo artículo en la lengua materna. (E) Porcentaje de trabajos cuya redacción en inglés fue revisada por alguien como favor. (F) Porcentaje de trabajos cuya redacción en inglés fue revisada por un servicio profesional. Las líneas de regresión (con intervalos de confianza del 95% como áreas sombreadas) representan la relación estimada con el número de artículos publicados en inglés (mostrados en el eje log10-transformado), basándose en los resultados mostrados en las Tablas S2-5 y S7-8 (el nivel de ingresos no fue significativo y, por tanto, no se muestra en (C)). Los datos subyacentes a esta figura son datos brutos extraídos directamente de las preguntas de la encuesta, que nuestra aprobación ética nos impide compartir para garantizar la confidencialidad de los encuestados.

Las y los angloparlantes no nativos, especialmente de nacionalidades con bajo dominio del inglés, tienen más probabilidades de que sus artículos sean rechazados por las revistas debido a la redacción en inglés, en comparación con angloparlantes nativos (Fig. 2A, Tabla S9). Por ejemplo, en una comparación de los que han publicado un artículo en inglés, el 38,1% (31,6 - 44,5%) y el 35,9% (30,5 - 41,3%) de angloparlantes no nativos de nacionalidades con dominio moderado y bajo del inglés, respectivamente, han experimentado el rechazo de artículos debido a la escritura en inglés, mientras que sólo el 14,4% de angloparlantes nativos lo han hecho, lo que significa que la frecuencia de rechazo de artículos relacionados con el idioma es de 2,5 a 2,6 veces mayor para autores/as no nativos. Este resultado también respalda las conclusiones de trabajos recientes, según los cuales es menos probable que las revistas publiquen trabajos de investigadores/as de países en los que el inglés no es la lengua materna [25-27]. Del mismo modo, es más probable que se pida a angloparlantes no nativos que mejoren su redacción en inglés durante la revisión del trabajo (Fig. 2B, Tabla S10). Por ejemplo, el 42,5% y el 42,6% de angloparlantes no nativos de nacionalidades con un dominio moderado y bajo del inglés, respectivamente, en comparación con sólo el 3,4% de la población de angloparlantes nativos, afirman que a menudo/la mayoría de las veces/siempre se les pide que mejoren su redacción en inglés durante la revisión del trabajo. Esto equivale a una frecuencia 12,5 veces mayor de revisiones relacionadas con el idioma para las y los angloparlantes no nativos.

Las y los angloparlantes no nativos dedican más esfuerzos a difundir sus investigaciones en varios idiomas que angloparlantes nativos, ya sea mediante la publicación de sus trabajos en revistas de lengua no inglesa (Fig. S4), la preparación de resúmenes en lengua no inglesa de artículos en inglés (Fig. 2C, Tabla S11) o actividades de divulgación en dos o más idiomas (Fig. 2D, Tabla S12).


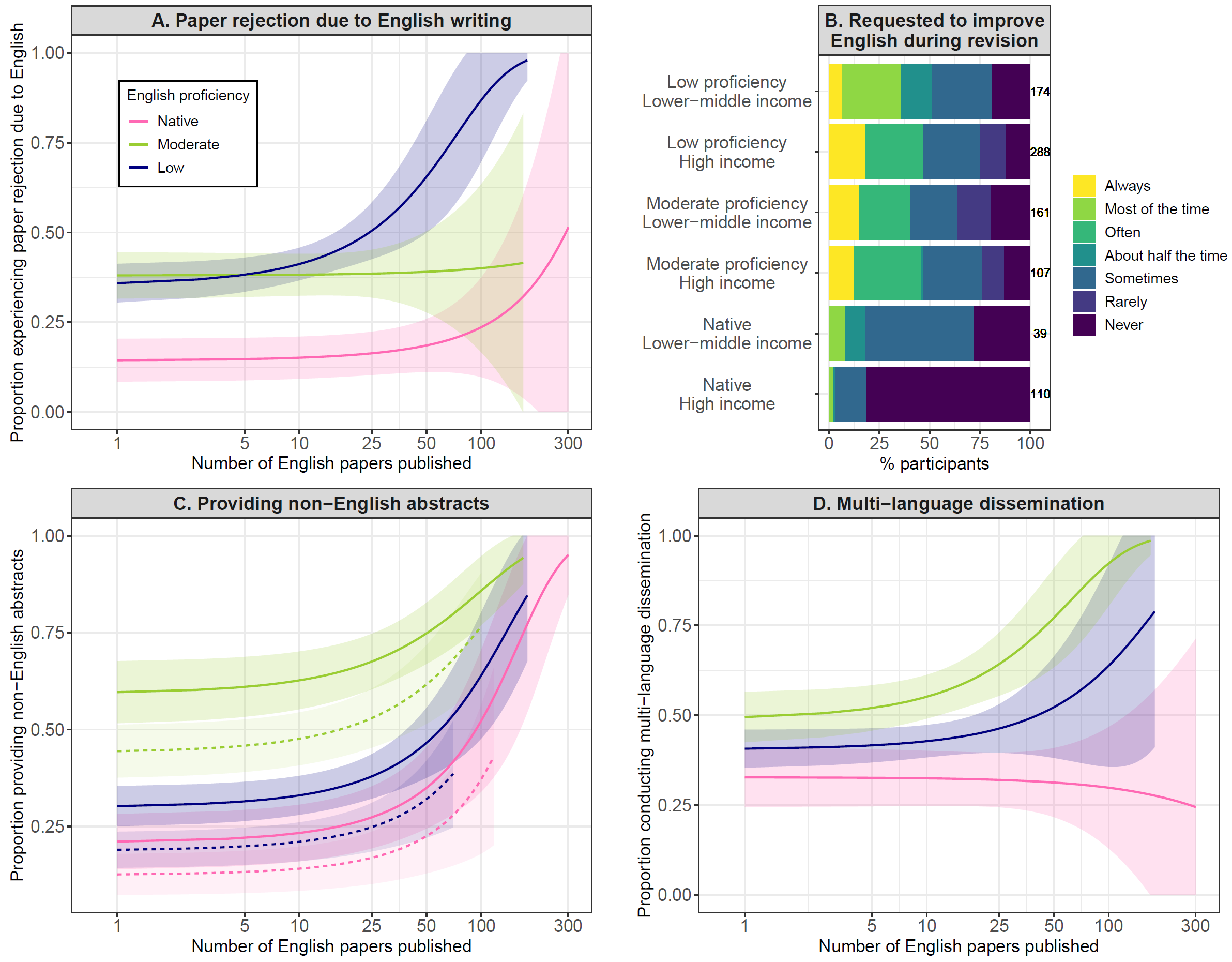


**Fig. 2. Barreras lingüísticas para la publicación y difusión de artículos.** (A) Proporción de investigadores/as a los que se les ha rechazado un primer artículo en inglés debido a la redacción en lengua inglesa. (B) Frecuencia con la que se les ha pedido que mejoren la redacción en inglés durante la revisión de sus primeros artículos en inglés. (C) Proporción de investigadores/as que han proporcionado resúmenes en lengua no inglesa de artículos en lengua inglesa. (D) Proporción de investigadores/as que han difundido artículos en inglés en otros idiomas además del inglés. Las líneas de regresión (con intervalos de confianza del 95% como áreas sombreadas) en (A), (C) y (D) representan la relación estimada con el número de artículos en inglés publicados (mostrados en el eje log10-transformado), basándose en los resultados mostrados en las Tablas S9, 11 y 12. El nivel de ingresos (línea continua: alto, línea discontinua: medio-bajo) sólo fue significativo y, por tanto, se muestra en (C). Los datos subyacentes a (A), (C) y (D) son datos brutos obtenidos directamente de las preguntas de la encuesta, que nuestra aprobación ética nos impide compartir para garantizar la confidencialidad de los encuestados. Los datos subyacentes a (B) se encuentran en Datos Suplementarios 1 (Data S1).

El idioma también puede ser un obstáculo importante para que las y los angloparlantes no nativos asistan a congresos. Aproximadamente el 30% de angloparlantes no nativos de nacionalidades con altos ingresos socioeconómicos (es decir, japoneses y españoles combinados) que inician su carrera (definida como aquellos que han publicado cinco o menos artículos en inglés) afirman que a menudo o siempre deciden no asistir a una conferencia en inglés debido a las barreras lingüísticas (Fig. 3A, Tabla S13). Del mismo modo, aproximadamente la mitad de las y los angloparlantes no nativos de nacionalidades con ingresos elevados (japoneses y españoles combinados) evitan a menudo o siempre las presentaciones orales debido a las barreras lingüísticas (Fig. 3B, Tabla S14).


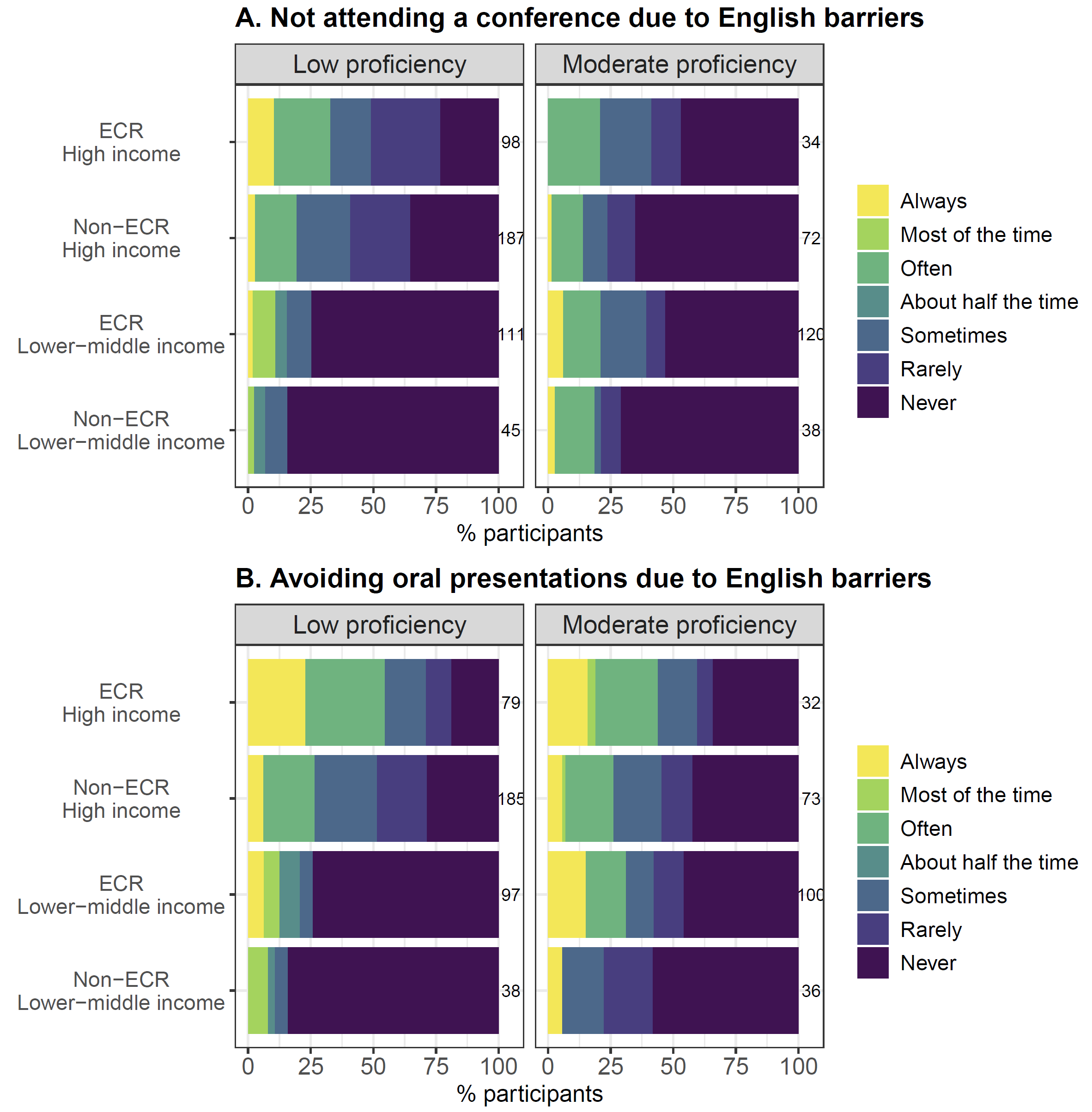


**Fig. 3.** **Barreras lingüísticas a la participación en congresos.** La frecuencia de (A) no asistir a una conferencia en inglés, y (B) evitar las presentaciones orales en una conferencia en inglés debido a la falta de confianza en la comunicación en inglés. Se definió como ECR (early-career researcher) a alguien con cinco o menos artículos en inglés. Los números a la derecha de cada barra representan el tamaño de la muestra. Los datos en los que se basa esta figura pueden consultarse en Datos S1.

Incluso si deciden hacer una presentación oral en inglés, las y los angloparlantes no nativos necesitan mucho más tiempo para preparar la presentación que angloparlantes nativos; los de nacionalidades con un dominio moderado y bajo del inglés dedican respectivamente una mediana del 93,7% (percentiles 2,5 - 97,5: 54,7 - 145,2%) y del 38,0% (10,8 - 69,6%) más de tiempo a preparar una presentación oral en inglés que los hablantes nativos de inglés (Figs. 4A, Tabla S15). Esta desventaja no cambia con el nivel profesional (Fig. S5) y, una vez más, no se aplica cuando se prepara una presentación en la lengua materna. Por ejemplo, angloparlantes no nativos de nacionalidades con bajo dominio del inglés dedican incluso menos tiempo a preparar una presentación en su lengua materna que angloparlantes nativos (Fig. 4B, Tabla S16). En los congresos, las y los angloparlantes no nativos suelen tener dificultades para explicar su trabajo en inglés. Esta tendencia es particularmente notable en las y los angloparlantes no nativos de nacionalidades con bajo nivel de inglés, ya que más del 65% afirma que a menudo o siempre les resulta difícil explicar su trabajo con seguridad en inglés (Fig. 4C, Tabla S17).


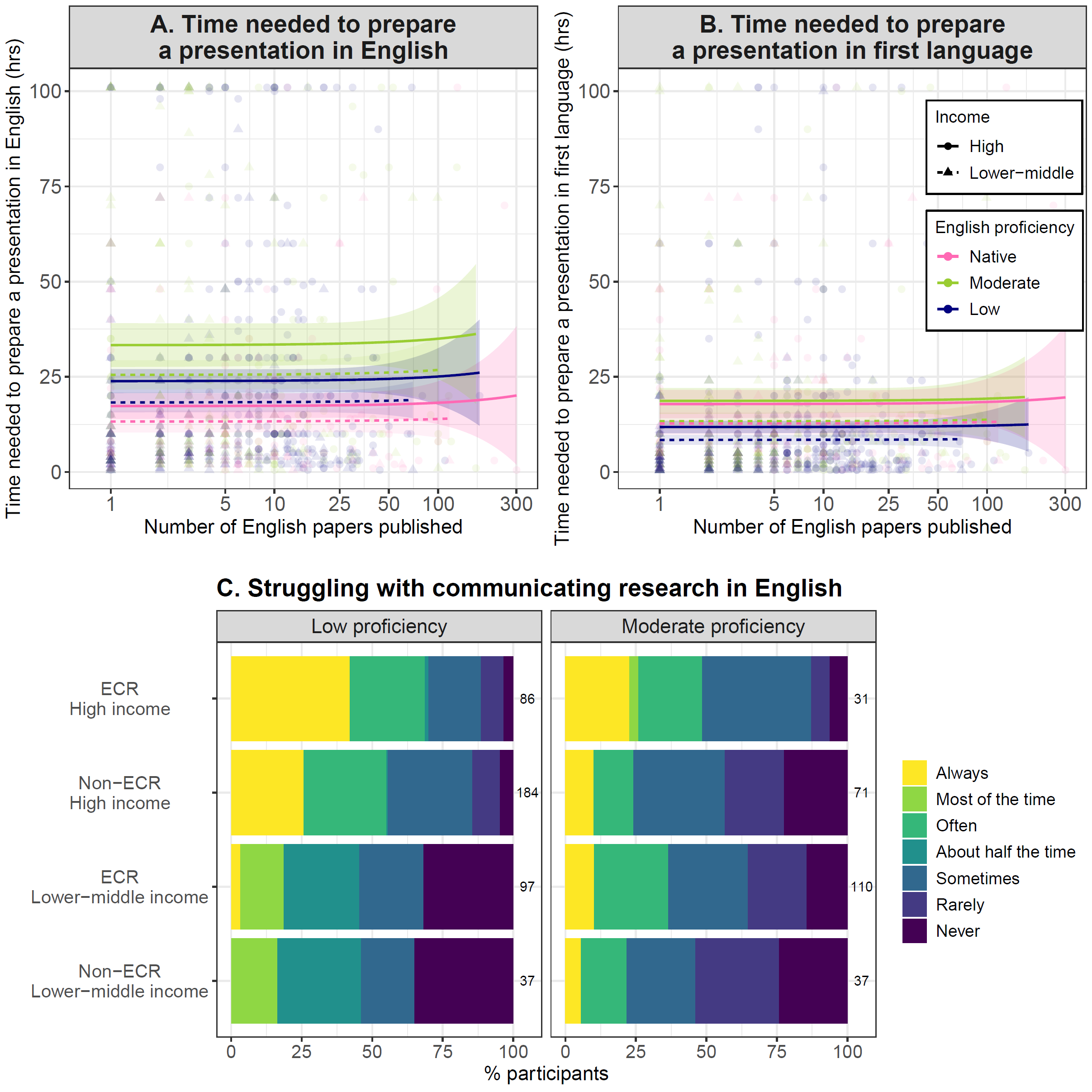


**Fig. 4. Barreras lingüísticas para preparar y realizar presentaciones en inglés.** (A) Número de horas necesarias para preparar y practicar una presentación oral en inglés. (B) Número de horas que se necesitan para preparar y practicar la misma presentación oral en la lengua materna. (C) Frecuencia de no poder explicar la investigación con seguridad durante una presentación debido a barreras lingüísticas en inglés. Las líneas de regresión (con intervalos de confianza del 95% como áreas sombreadas) en (A) y (B) representan la relación estimada con el número de artículos publicados en inglés (mostrados en el eje log10-transformado), basándose en los resultados mostrados en las Tablas S15 y 16. En (C) un ECR (número de artículos publicados en inglés) es el número de artículos publicados en inglés. En (C) se definió a un ECR (investigador novel) como alguien con cinco o menos artículos en inglés publicados hasta la fecha. Los números a la derecha de cada barra representan el tamaño de la muestra. Los datos subyacentes a (A) y (B) son datos brutos obtenidos directamente de las preguntas de la encuesta, que nuestra aprobación ética nos impide compartir para garantizar la confidencialidad de los encuestados. Los datos subyacentes (C) se encuentran en Datos S1.

En conjunto, los resultados de este estudio ilustran las múltiples barreras lingüísticas a las que se enfrentan los y las angloparlantes no nativos en el ejercicio de la ciencia, y los costos que suponen para el desarrollo de sus carreras científicas (Fig. 5). A manera de ejercicio, imaginemos que somos estudiantes de doctorado cuya lengua materna no es el inglés. Así pues, en comparación con un/a colega que sea hablante nativo de inglés, necesitaríamos considerablemente más tiempo, y dinero probablemente, para comprender cada uno de los artículos en inglés que leamos, lo que nos llevaría a dedicar hasta 19.1 días laborables más al año a esta actividad (Véase el cálculo en la Fig. S1), escribir los capítulos de la tesis en inglés y pulir la redacción en inglés antes de enviar nuestros manuscritos a revistas científicas. Una vez enviamos nuestros artículos, también tendríamos dificultades en el proceso de revisión y publicación, ya que podrían ser rechazados con más frecuencia y, seguramente, se nos pediría revisar el inglés del manuscrito. Finalmente, si lográramos publicar el artículo, tendríamos que hacer un esfuerzo adicional para su difusión, ya que necesitaremos divulgarlo en inglés y en nuestro(s) propio(s) idioma(s). También tendríamos dudas a la hora de asistir a una conferencia internacional o realizar una presentación oral de nuestro trabajo, perdiendo oportunidades de desarrollar una red de colaboración internacional, debido a que necesitaríamos un mayor tiempo para prepararla y la sensación de frustración por no poder presentar en inglés con la misma eficacia que en nuestra lengua materna. Todas estas barreras seguirán interponiéndose en tu camino mientras sigas en la carrera investigadora.


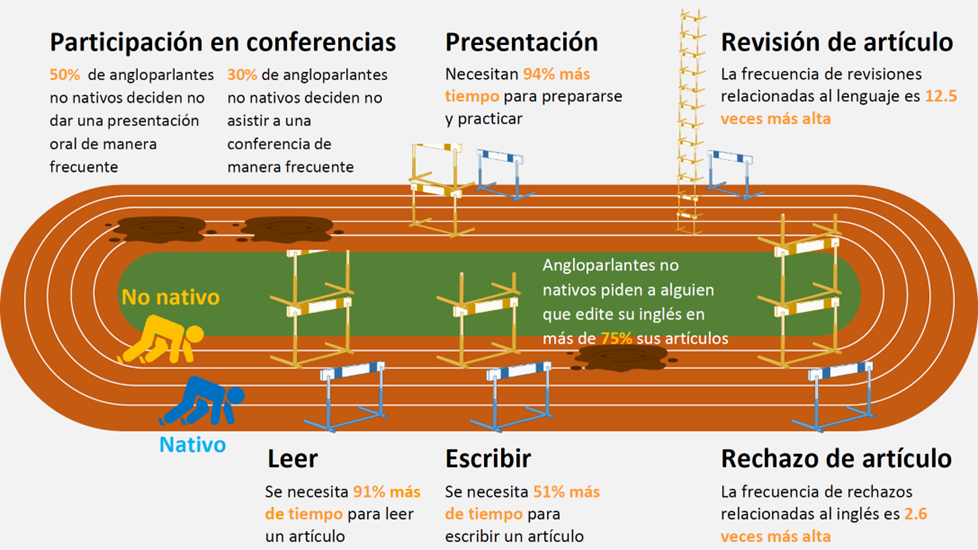


**Fig 5**. **Desventajas estimadas de angloparlantes no nativos en la realización de diferentes actividades científicas.** La altura de los obstáculos permite comparar el esfuerzo realizado por angloparlantes no nativos en términos del tiempo necesario para leer (Lectura), (Escritura) y preparar una presentación oral en inglés (Presentación), y la frecuencia relativa con que un artículo en inglés fue rechazado (Rechazo de artículo) o se ha hecho la solicitud de reevaluar la escritura del inglés (Revisión de artículo). Los valores corresponden a hablantes no nativos de inglés que solo han publicado un artículo en ese idioma (se tomó el valor más alto entre las nacionalidades con competencia en inglés moderada y baja), en comparación con los valores para los hablantes nativos de inglés. La analogía usada en la figura no pretende hacer una apología a la ciencia como una competencia.

Dadas todas estas desventajas, en la igualdad de condiciones, la productividad científica aparente de las y los angloparlantes no nativos sería sin duda muy inferior a la de las y los angloparlantes nativos. Estas desventajas conducen inevitablemente a una tremenda desigualdad en el desarrollo de las carreras científicas entre angloparlantes nativos y los no nativos y a una grave infrarrepresentación de la investigación de los países en los que el inglés no es lengua materna en las publicaciones en lengua inglesa [28]. Además, a mayor escala, una consecuencia clara de esta desigualdad es la pérdida de oportunidades de las comunidades científicas de incorporar a muchos investigadores/as y conocimientos asociados en las primeras fases de sus carreras, en parte porque su primera lengua resulta ser una distinta del inglés. Esto puede reflejarse en nuestra observación de que algunas desventajas parecían desaparecer en los investigadores/as al final de su carrera (Figs. S1 y S2). Sospechamos que esto podría deberse a un sesgo de supervivencia; es posible que sólo aquellos/as angloparlantes no nativos/as que han conseguido hacer ciencia en inglés tan eficientemente como los hablantes nativos de inglés hayan permanecido en una carrera investigadora y, por tanto, hayan sido el grupo dominante entre investigadores/as experimentados que participaron en esta encuesta.

La infrautilización de los servicios profesionales de edición en inglés por parte de las personas de nacionalidades con ingresos socioeconómicos más bajos, debida presumiblemente a la falta de financiación, indica que las desventajas para las y los angloparlantes no nativos podrían verse amplificadas por el bajo nivel de ingresos de un país y del individuo. Las barreras lingüísticas para algunas actividades científicas, como la lectura de artículos (Fig. 1A), la preparación de presentaciones orales (Fig. 4A) y la asistencia y presentación en congresos (Figs. 3A-B, 4C), parecen afectar menos a las personas de nacionalidades con ingresos más bajos. Esto podría explicarse una vez más por el sesgo de supervivencia. Aparte de los idiomas que se hablan en los países de renta alta, como el español y el japonés, pocos idiomas que no sean el inglés tienen un léxico actualizado de términos científicos, lo que crea una necesidad mucho mayor de que sus hablantes reciban formación científica en inglés [29]. En los países de renta baja, es posible que sólo aquellos que pueden permitirse recibir esa formación en inglés hayan podido convertirse en investigadores/as y participar en nuestra encuesta.

Es probable que este estudio haya subestimado la gravedad de las desventajas a las que se enfrentan las y los angloparlantes no nativos. Por ejemplo, no cuantificamos el inmenso estrés mental asociado a todo el tiempo extra, el costo, el esfuerzo y las oportunidades perdidas a causa de las barreras lingüísticas, lo que podría agravar aún más el ya elevado riesgo de problemas de salud mental en estudiantes e investigadores/as jóvenes [30]. Las y los angloparlantes no nativos también podrían enfrentarse al dilema de adaptarse a la realización y comunicación de la ciencia en inglés o mantener sus habilidades en la realización y comunicación de la ciencia en sus lenguas maternas [29]. Lo más probable es que los/as participantes en la encuesta sean personas que actualmente se dedican a la investigación, por lo que es probable que la encuesta haya excluido a quienes la han abandonado debido a las barreras lingüísticas. También pueden existir otros sesgos en los participantes de la encuesta (véase Limitaciones en Materiales y Métodos para su discusión). Aunque la encuesta se diseñó para aislar las desventajas asociadas únicamente a las barreras lingüísticas, no podemos descartar la posibilidad de que el costo que hemos cuantificado pueda incorporar, al menos en parte, el costo asociado a otras barreras en la ciencia, como las económicas, sociales, de identificación y de inmigración, que muchos académicos de países en los que el inglés no está muy extendido suelen experimentar [23, 24, 31]. Aunque esto puede ser una posible limitación de este estudio, lo que implica en la práctica es que las desventajas a las que se enfrentan las y los angloparlantes no nativos podrían ser aún mayores y más polifacéticas. El nivel de desventajas para las y los angloparlantes no nativos podría variar entre disciplinas, presumiblemente dependiendo, por ejemplo, de la historia de la educación basada en el inglés y de la necesidad de colaboración internacional. Por lo tanto, aunque creemos que el problema de las barreras lingüísticas para el desarrollo profesional de las y los angloparlantes no nativos es generalizado, las conclusiones de este estudio pueden no ser aplicables cuantitativamente a todas las disciplinas.

Hasta la fecha, la tarea de superar las barreras lingüísticas se ha dejado en gran medida en manos de angloparlantes no nativos y de su inversión en formas de mejorar sus conocimientos de inglés. Sin embargo, la magnitud de la desventaja, cuantificada en este estudio, parece muy superior al nivel que puede superarse con el esfuerzo de los individuos. Necesitamos urgentemente un esfuerzo concertado, a nivel institucional y social, para minimizar las desventajas de las y los angloparlantes no nativos. Sostenemos que todos los sectores de la ciencia, desde supervisores y colaboradores hasta las universidades, instituciones, revistas, financiadores y congresos, deberían tomar medidas inmediatas para proporcionar apoyo lingüístico a las y los angloparlantes no nativos y tener en cuenta explícitamente esas desventajas a la hora de evaluar sus resultados científicos (véase la Fig. 6 para las soluciones propuestas). Un aspecto clave de estas soluciones es aceptar la diversidad lingüística en la ciencia y fomentar la multilingüización de la ciencia y su comunicación, ya que esto puede ayudar a mejorar la equidad, la diversidad y la inclusión en la ciencia [14] y maximizar la contribución de la ciencia para abordar algunos de los retos globales [32, 33]. Nuestra encuesta mostró el uso relativamente bajo de la traducción automática por parte de los investigadores de todos los países (Fig. S6). Sin embargo, las herramientas emergentes de Inteligencia Artificial (IA), como ChatGPT (https://chat.openai.com/) y DeepL (https://www.deepl.com/), podrían ayudar a los hablantes no nativos de inglés, especialmente a los de países con bajos ingresos, a reducir el esfuerzo y el costo necesarios para realizar algunas de las actividades científicas, proporcionando una corrección/traducción del inglés gratuita o asequible [34, 35]. Aunque todavía se está debatiendo sobre el uso de la IA en la ciencia [36, 37], creemos que las revistas y las universidades deberían considerar y permitir el uso adecuado de herramientas de IA para la corrección del inglés con el fin de reducir las barreras lingüísticas y mejorar la equidad en la ciencia.


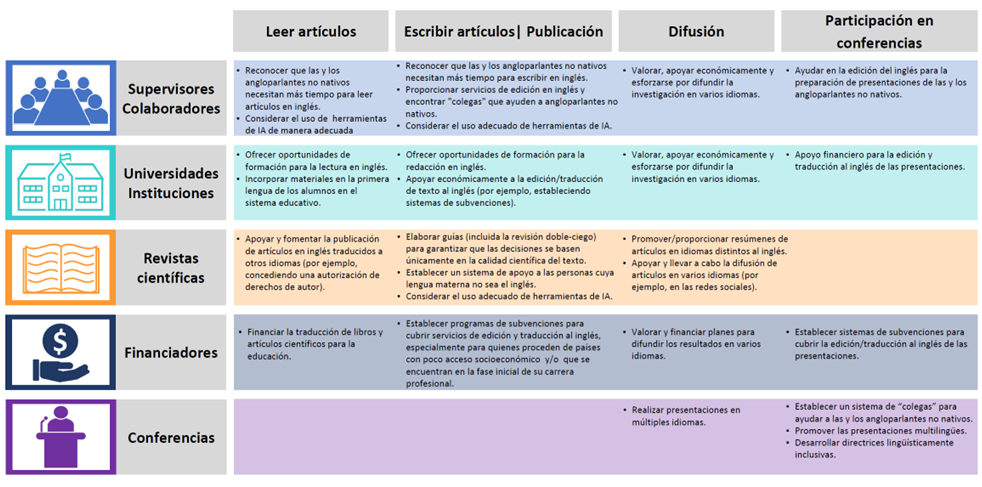


**Fig 6**. **Ejemplos de posibles soluciones para reducir las desventajas de los angloparlantes no nativos en cada tipo de actividad científica.** IA: inteligencia artificial. También consulte [35, 38, 39] para otras posibles soluciones.

La desigualdad a la que se enfrentan las y los angloparlantes no nativos debido a las barreras lingüísticas puede ser una de las principales razones de la actual infrarrepresentación de las y los angloparlantes no nativos en las actividades científicas mundiales [40]. Un comentario de un participante en la encuesta nos llamó la atención: *“Si no fuera por la barrera del idioma, podría haber contribuido mucho más al avance de la ecología y la conservación de la biodiversidad”* (participante japonesa de entre 40 y 50 años).

Las y los angloparlantes no nativos constituyen el 95% de la población mundial [41]. Imagínese cuántos angloparlantes no nativos de todo el mundo y a lo largo del tiempo se han sentido frustrados, al igual que este participante, por no poder contribuir al avance de la ciencia en la medida de sus posibilidades. Piense en cuántos/as colaboradores potenciales no se han incorporado a las comunidades científicas debido a las barreras lingüísticas. Dada la multitud de retos acuciantes a los que se enfrentan la humanidad y este planeta, no podemos permitirnos perder la contribución de una fuente de investigadores/as tan prometedora, tan necesaria y, sin embargo, tan desaprovechada.

**Materiales y métodos**

Declaración ética

La encuesta se realizó entre junio y octubre de 2021 con la Aprobación Ética Institucional de Investigación Humana de la Universidad de Queensland (número de aprobación: 2021/HE000566). Todos los participantes tenían al menos 18 años y dieron su consentimiento por escrito indicando que estaban de acuerdo en participar en la encuesta. En la hoja de información al participante se aclaró que la participación es de carácter, como también los objetivos de la investigación, cómo se utilizarían los datos y que los mismos serían confidenciales.

Los objetivos de la encuesta fueron (i) cuantificar la cantidad de esfuerzo requerido por los investigadores individuales para llevar a cabo cinco tipos de actividades científicas en inglés y en su lengua materna: lectura de artículos, redacción, publicación y difusión, y participación en congresos, y (ii) comparar la cantidad estimada de esfuerzo entre investigadores con diferentes antecedentes lingüísticos y económicos.

Participantes objetivo

Para la comparación entre investigadores de distintos antecedentes lingüísticos y económicos, seleccionamos ocho nacionalidades: bangladesí, boliviana, británica, japonesa, nepalí, nigeriana, española y ucraniana. Estas nacionalidades se estratificaron según los niveles de dominio del inglés de cada país (basados en el Índice de Proficencia de Ingles [21]) y los ingresos (basados en la lista de economías del Banco Mundial [22]): Bangladesh, Nepal (dominio bajo del inglés e ingresos medios-bajos), Japón (dominio bajo del inglés e ingresos altos), Bolivia, Ucrania (dominio moderado del inglés e ingresos medios-bajos), España (dominio moderado del inglés e ingresos altos), Nigeria (inglés como lengua oficial e ingresos medios-bajos) y Reino Unido (inglés como lengua oficial e ingresos altos). Nos centramos en el dominio del inglés y el nivel de ingresos basándonos en nuestra hipótesis de que la cantidad de esfuerzo necesaria para llevar a cabo actividades científicas en inglés sería mayor en los hablantes no nativos de inglés de países con menor dominio del inglés y nivel de ingresos más bajo.

Tenga en cuenta que el nivel de inglés de los países no refleja necesariamente el nivel de inglés de cada participante. Sin embargo, el nivel de dominio del inglés de los países estaba significativamente relacionado con dos de las otras tres medidas de la experiencia de los participantes en la comunicación con el inglés Esto respalda el uso del nivel de inglés de los países como medida aproximada del nivel de inglés de los participantes.

Los niveles de ingreso de los países tampoco reflejan necesariamente el nivel socioeconómico de cada participante. Por lo tanto, este estudio no puede evaluar el efecto de los antecedentes socioeconómicos de los individuos.

La encuesta fue dirigida a cualquier persona de cualquier profesión y de cualquier nivel profesional y que tuviera la nacionalidad de los países seleccionados y hubiera publicado al menos un artículo en inglés, revisado por pares y del que fuera el primer autor, sobre ecología, biología evolutiva, biología de la conservación o disciplinas afines.

Cuestionario

La encuesta (que figura en el texto complementario S1) consta de seis secciones. La primera sección (P1.1-1.2) trata sobre la primera lengua de los participantes (definir la primera lengua de los participantes es difícil en algunos países, como Nigeria, pero utilizamos la siguiente definición: “la(s) lengua(s) que aprendió a hablar en casa de niño”) y la nacionalidad; esta información se utilizó para filtrar a los participantes elegibles. La segunda sección (P2.1-2.7) contiene preguntas sobre información general, incluidas medidas del dominio del inglés; se utilizaron para tener en cuenta factores que pueden afectar a las respuestas a las otras preguntas de la encuesta durante el análisis, y para justificar el uso del dominio del inglés de los países en el análisis. La tercera sección (P3.1-3.7) incluye preguntas sobre la experiencia de los participantes con las barreras lingüísticas a la hora de redactar trabajos en inglés. En la cuarta sección (P4.1-4.5) se pregunta sobre la experiencia de los participantes con las barreras lingüísticas en la publicación y difusión de artículos. La quinta sección (P5.1-5.3) trata sobre las consecuencias de las barreras lingüísticas en la lectura de artículos en inglés, y la sexta sección (P6.1-6.6) se pregunta cómo pueden haber afectado las barreras lingüísticas a las experiencias de los participantes en torno a la asistencia a congresos científicos. La encuesta también permitió a los participantes hacer comentarios sobre la misma, así como comentarios generales sobre el proyecto.

Para que los participantes pudieran estimar el tiempo necesario para realizar cada actividad científica con la mayor precisión posible, les pedimos que facilitaran datos sobre experiencias reales, es decir, cuánto tiempo les llevó escribir el último artículo que redactaron (P3.3), leer el último artículo que leyeron (P5.1) y preparar la última presentación oral que hicieron (P6.4) en inglés. A los hablantes no nativos de inglés, les pedimos que calcularan el tiempo que necesitarían para escribir el mismo artículo (P3.4), leer el mismo artículo (P5.2) y preparar la misma presentación (P6.5) pero en su lengua materna. Véasela en la sección Limitaciones una discusión de las posibles consecuencias de este enfoque a la hora de extraer conclusiones.

Al preguntar por la frecuencia, utilizamos una escala de Likert de cinco puntos: Siempre, A menudo, A veces, Rara vez o Nunca. Nuestras preguntas también se diseñaron para preguntar a los participantes sobre las experiencias que relacionadas únicamente a las barreras lingüísticas, y no a otras barreras, a menudo confusas (por ejemplo, indicando la parte en negrita: “6.2 *¿Con qué frecuencia ha decidido no asistir a una conferencia en inglés (ya sea para presentar su investigación o simplemente para participar)* ***porque no tenía la suficiente confianza para comunicarse en inglés****?”*. Véanse también otras preguntas en el texto complementario S1).

Para maximizar la tasa de respuesta, la encuesta se tradujo a los idiomas pertinentes para cada nacionalidad (bangla para bangladeshí traducido por SC, japonés para japonés por TA, nepalí para nepalí por KP, español para boliviano y español por VB-E, y ucraniano para ucraniano por MG), y se implementó como una encuesta en línea separada para cada nacionalidad en Qualtrics.

Distribución de la encuesta

En primer lugar, identificamos coordinadores (de aquí en más, coordinadores país) para cada uno de los ocho países seleccionados, basado en dos criterios (i) que fueran hablantes nativos de la lengua oficial del país y (ii) que dispusieran de una buena red entre los investigadores de las disciplinas de estudio del país. Todos los coordinadores nacionales participaron en este estudio como coautores (TA por Japón, IB por Nigeria, SC por Bangladesh, MG por Ucrania, JDG-T por España, FM-C por Bolivia, KP por Nepal y RW por el Reino Unido). Los coordinadores país tenían como objetivos obtener al menos 100 respuestas a la encuesta de su respectivo país. Intentamos distribuir la encuesta de la forma más imparcial posible. Para conseguirlo, adoptamos, en principio, uno o todos de los cuatro métodos siguientes de distribución de la encuesta dentro de cada país, basándonos en conversaciones con los coordinadores país sobre qué método o métodos podrían ser los mejores para ese país:

- Distribuir la encuesta a través de las principales listas de correo de investigadores de las disciplinas pertinentes.
- Pedir a las sociedades académicas de las disciplinas pertinentes que distribuyan la encuesta entre sus miembros.
- Identificar hasta diez universidades e instituciones con departamentos, escuelas o divisiones relevantes dentro del país y pedirles que distribuyan la encuesta entre sus investigadores afiliados.
- Identificar a investigadores que hayan publicado un artículo en inglés en una disciplina relevante y estén afiliados a una institución del país en sistemas de búsqueda de bibliografía y enviarles directamente la encuesta por correo electrónico.

Evitamos en la medida posible utilizar nuestras redes personales (incluidas las cuentas personales en las redes sociales) para difundir la encuesta, con el fin de reducir posibles sesgos en el reclutamiento de participantes (véanse las excepciones para Bangladesh más adelante). A continuación, se describe detalladamente el método de distribución de la encuesta en cada país (todas las fechas se refieren a 2021).

*Bangladesh*

En Bangladesh no pudimos encontrar ninguna lista de correo relevante. Existen sociedades académicas, pero los investigadores noveles no pertenecen necesariamente a ellas, por lo que decidimos no distribuir la encuesta tampoco a través de las sociedades académicas. En su lugar, la encuesta se distribuyó contactando directamente a siete universidades y un total de 232 investigadores individuales identificados en Google Scholar y Facebook.

22 y 27 de junio: Difusión de la encuesta en la cuenta personal de Facebook del coordinador nacional.

14-18 de julio: Se contactó con representantes de cuatro grandes universidades (Universidad de Dhaka, Universidad de Jahangirnagar, Universidad de Ciencia y Tecnología de Pabna y Universidad de Ciencia y Tecnología de Noakhali) y se les pidió que difundieran la encuesta en sus departamentos correspondientes.

25 de julio: Se re contacto con representantes de tres universidades (Universidad de Dhaka, Universidad de Jagannath y Universidad de Ciencia y Tecnología de Noakhali) y se les pidió que difundieran la encuesta en sus departamentos correspondientes. También se envió un correo electrónico a un profesor de la Universidad de Dhaka para que compartiera la encuesta con sus colegas, que a su vez la compartieron con muchos otros profesores.

31 de julio: Se vuelve a contactar a un representante de la Universidad de Dhaka y a representantes de otras tres universidades (la Universidad Agrícola de Sher-e-Bangla, la Universidad Agrícola de Bangladesh y la Universidad de Chittagang) y se les pide que difundan la encuesta en sus departamentos correspondientes.

8 de agosto: Se volvió a compartir la encuesta en las cuentas personales de Facebook y Twitter del coordinador nacional.

12 de septiembre: Envío directo por correo electrónico de la encuesta a los 100 principales investigadores de Bangladesh identificados en Google Scholar (búsqueda con (conservación O ecología O evolución) Y Bangladesh).

22 de septiembre - 15 de octubre: Contacto con 120 investigadores de disciplinas relevantes identificados en Facebook.

28 de octubre: Se compartió la encuesta en las cuentas personales de Facebook y LinkedIn del coordinador país, y también se contactó con 12 investigadores al mismo tiempo que se envió un recordatorio a los que ya habían sido contactados.

*Bolivia*

En Bolivia la encuesta se distribuyó a través de una importante lista de correos electrónicos y contactando con cuatro sociedades, cinco universidades, cuatro museos/herbarios y un total de 72 investigadores individuales identificados en la Web of Science.

29 de junio: Difusión de la encuesta en una importante lista de correo de biólogos y ecólogos de Bolivia. Se enviaron recordatorios una vez en junio y otra en julio. La encuesta también se envió a la Organización de Mujeres en la Ciencia de Bolivia, la Asociación Boliviana de Ornitólogos, la Asociación Boliviana de Mastozoólogos y la Sociedad Boliviana de Entomólogos para que la compartieran en sus listas de correo.

1 de julio: Se contactó a los Directores de los Departamentos de Biología, Zoología, Botánica y Ecología de las cinco universidades que tienen departamento de ciencias en Bolivia (Universidad Mayor de San Andrés, Universidad Amazónica de Pando, Universidad Mayor Gabriel Rene Moreno, Universidad Mayor de San Simón, y Universidad San Francisco Xavier de Chuquisaca) y a los cuatro principales museos/herbarios de Bolivia (Colección Boliviana de Fauna, Herbario Nacional de Bolivia, Museo de Historia Natural Noel Kempff Mercado y Museo Nacional Martín Cárdenas) y se les pidió que difundieran la encuesta en sus departamentos. Se les envió un recordatorio el 26 de julio.

16 de septiembre: Se realizaron búsquedas en Web of Science (utilizando todas las bases de datos) con: ALL=((conservation OR ecolog* OR evolution*) AND (Bolivia)). La búsqueda arrojó 3715 estudios, de los cuales se identificaron 72 primeros autores que parecían ser bolivianos. La encuesta se compartió directamente con los 72 autores por correo electrónico. En el caso de los autores que no estaban accesibles a través de las direcciones de correo electrónico de los artículos, el coordinador país buscó sus nuevas direcciones de contacto (en ORCID y otras plataformas) y, si las encontró, utilizó las nuevas direcciones para ponerse en contacto con ellos.

*Japón*

En Japón, la encuesta se difundió a través de dos importantes listas de correo.

9 de junio: Se compartió la encuesta en las dos principales listas de correo para ecólogos (Jeconet, con 3500 usuarios a partir de 2014) y biólogos evolutivos (evolve, con 2500 usuarios a partir de 2016) en Japón.

23 de junio: Envío de un correo electrónico de seguimiento a las dos mismas listas de correo.

*Nepal*

En Nepal se compartió la encuesta con cinco sociedades y cinco universidades.

2 de julio: Se pidió a la Nepal Environment Society, la Environmental Graduates in Himalaya, la Society for Conservation Biology Nepal Chapter, la Botanical Society of Nepal y la Zoological Society of Nepal (en total, estas sociedades tienen más de 600 miembros) que compartieran la encuesta en sus listas de correo.

27 de julio: Envío de recordatorios a las personas contactadas anteriormente.

5 de septiembre: Contacto telefónico con los jefes de departamento de cinco universidades con programas de conservación de la biodiversidad y ciencias naturales (Universidad de Katmandú, Universidad de Tribhuvan, Universidad de Pokhara, Universidad del Medio Oeste y Universidad de Agricultura y Silvicultura) para pedirles que difundan la encuesta en sus departamentos.

20 de septiembre: Envío de recordatorios a las mismas universidades.

*Nigeria*

En Nigeria, la encuesta se distribuyó poniéndose en contacto con tres sociedades relevantes, tres institutos con departamentos relevantes, cinco universidades (de cinco de las seis zonas geopolíticas de Nigeria) y un total de 54 investigadores individuales identificados en Google Scholar.

21 de junio: Se compartió la encuesta con el grupo de antiguos alumnos de la Asociación Nigeriana de Biología Tropical, científicos del Centro Nacional de Recursos Genéticos y Biotecnología e investigadores del Departamento de Zoología de la Universidad de Lagos.

22 y 23 de junio: Se compartió la encuesta con científicos del Complejo Científico y Tecnológico de Sheda.

6 de julio: Contacto con el secretario adjunto de la Sociedad Zoológica de Nigeria, que compartió la encuesta con todos los miembros de la sociedad (unas 400 personas).

8 de julio: Difusión de la encuesta entre 36 facultades de los departamentos de Botánica, Gestión de Recursos Forestales, Vida Silvestre y Ecoturismo, Química, Geografía y Geología de la Universidad de Ibadán.

10 de julio: Compartimos la encuesta en Whatsapp con todos los científicos del Instituto de Investigación del Cacao de Nigeria, una institución del gobierno federal con más de 200 investigadores.

14 de julio: Envío de recordatorios al grupo de antiguos alumnos de la Asociación Nigeriana de Biología Tropical, a los científicos del Centro Nacional de Recursos Genéticos y Biotecnología y a los investigadores del Departamento de Zoología de la Universidad de Lagos.

12 de septiembre: Se compartió la encuesta con 60 profesores de la Universidad Adekule Ajasin y uno de la Universidad Abubakar Tafawa Balewa.

14 de octubre: Se compartió la encuesta con 63 profesores de la Universidad Ahmadu Bello.

18 de octubre: Se compartió la encuesta con 173 miembros de la Society for Conservation Biology Nigerian Chapter, y 54 autores identificados a través de búsquedas en Google Scholar utilizando: "(conservación OR ecología OR evolución) AND Nigeria".

*España*

En España, la encuesta se compartió con cinco sociedades, 19 universidades y un museo. Elegimos de una a cuatro universidades con un departamento de biología fuerte de cada una de las nueve, de las 17, comunidades autónomas de España, de modo que las universidades seleccionadas están geográficamente dispersas.

21 de junio: Pedimos a la Sociedad Limnológica, la Sociedad de Ecólogos Terrestres, la Sociedad de Biología Evolutiva, la Sociedad de Bioquímica y Biología Molecular y la Sociedad de Biología Celular que compartieran la encuesta con sus miembros a través de sus canales.

5 de julio: Envío del primer recordatorio a las cinco sociedades mencionadas.

30 de agosto: Envío de un segundo recordatorio a las cinco sociedades. Pedimos a los departamentos de biología/ciencias de nueve universidades de todo el país que compartieran la encuesta en sus departamentos: Universidad de Barcelona, Universidad Autónoma de Barcelona, Universidad de Girona, Universidad Complutense de Madrid, Universidad de Sevilla, Universidad de Valencia, Universidad de Cádiz, Universidad de Murcia y Universidad del País Vasco.

13 de septiembre: Envío de un tercer recordatorio a las cinco primeras sociedades, y del primer recordatorio a las nueve universidades adicionales.

4 de octubre: Cuarto recordatorio a las cinco sociedades y segundo recordatorio a las nueve universidades. Se pidió a otras diez universidades y a un museo que compartieran la encuesta en sus redes: Universidad del Rey Juan Carlos, Universidad Autónoma de Madrid, Universidad de Salamanca, Universidad de Huelva, Universidad de Málaga, Universidad de Burgos, Universidad de León, Universidad de Castilla y La Mancha, Universidad de Alicante, Universidad de Zaragoza y Museo de Ciencias Naturales de Madrid.

18 de octubre: Envío de recordatorios a las cinco sociedades, 19 universidades y el museo.

25 de octubre: Envío de recordatorios a las cinco sociedades, 19 universidades y el museo.

*Ucrania*

En Ucrania, la encuesta se difundió a través de diez universidades, tres institutos, tres grupos de Facebook y un total de 139 investigadores individuales identificados en la Web of Science, resúmenes de congresos y revistas ucranianas.

29 de junio: La encuesta se distribuyó entre los empleados del Museo Estatal de Historia Natural (Lviv); el administrador del museo también la publicó en el grupo de Facebook Flora de Ucrania. Se pide al Instituto de Ecología de los Cárpatos, NASU (Lviv), que comparta la encuesta en su red.

22 de julio: Petición al Instituto de Zoología I.I. Schmalhausen de la Academia Nacional de Ciencias de Ucrania (NASU) (Kiev) para que comparta la encuesta en su red.

13 de septiembre: Compartimos la encuesta con todos los investigadores del Instituto de Biología Marina de la NASU (Odesa) y con 139 investigadores identificados en la Web of Science (utilizando las palabras clave: All=((conservation OR ecolog* OR evolution*) AND (Ukraine))) y mediante la búsqueda de resúmenes de congresos en Google (con las palabras clave: "еволюційна біологія конференція", "охорона природи конференція", o "екологія конференція").

14 de septiembre: Preguntamos a los departamentos de biología/ecología de diez universidades (Khmelnytsk

14 de septiembre: Se pide a los departamentos de biología/ecología de diez universidades (Universidad Nacional de Khmelnytsky, Universidad Nacional del Mar Negro Petro Mohyla, Universidad Estatal de Sumy, Universidad Nacional de Ingeniería del Agua y Medio Ambiente, Universidad Nacional de Ciencias de la Vida y Medio Ambiente de Ucrania, Universidad Nacional de Agricultura de Poltava, Universidad Nacional de Silvicultura de Ucrania, Universidad Técnica Nacional de Petróleo y Gas de Ivano-Frankivsk, Universidad Nacional de Chernivtsi y Museo Nacional de la Universidad Nacional de Chernivtsi) que compartan la encuesta en su red.

27 de septiembre: Envío de recordatorios a todos los investigadores con los que se contactó el 13 de septiembre.

11 de octubre: Envío de recordatorios a todos los investigadores con los que se contactó previamente.

11 de octubre: Compartida la encuesta en el grupo de Facebook Ukrainian Botanical Group.

13 de octubre: Difusión de la encuesta en el grupo de Facebook Ukrainian Scientists Worldwide.

*Reino Unido*

En el Reino Unido, la encuesta se difundió a través de tres sociedades/organismos profesionales, un instituto de investigación y 20 universidades.

- Sociedad Ecológica Británica (BES)

10 de junio: Se le pidió que difundiera la encuesta a través de sus canales.

25 de agosto: Envío de un recordatorio.

Las cuentas de Twitter de las revistas de la BES tuitearon sobre la encuesta:

7 de julio y 7 de septiembre @MethodsEcolEvol (26,3k seguidores)

13 de julio y 13 de septiembre @FunEcology (21,6k seguidores)

14 de julio y 7 de septiembre @jecology (30,7k seguidores)

9 de julio y 7 de septiembre @JAppliedEcology (31,4k seguidores)

7 de julio y 7 de septiembre @AER_ESE_BES (2,1k seguidores)

7 de julio y 7 de septiembre @AnimalEcology (22,7k seguidores)

7 de julio y 15 de septiembre @PaN_BES (4,6k seguidores)

- Real Sociedad de Biología (RSB)

10 de junio: Solicitud de difusión de la encuesta a través de sus canales.

25 de junio: La encuesta se compartió en su Science Policy Newsletter, que se envía a unas 26.000 personas, la mayoría en el Reino Unido.

25 de agosto: Envío de un recordatorio.

10 de septiembre: La encuesta se publica de nuevo en el boletín Science Policy Newsletter.

- Instituto Colegiado de Ecología y Gestión Medioambiental (CIEEM)

10 de junio: Solicitud de difusión de la encuesta a través de sus canales.

25 de agosto: Envío de un recordatorio.

- Centro de Ecología e Hidrología (CEH)

10 de junio: Difusión de la encuesta a través de sus canales.

1 de septiembre: CEH tuiteó sobre la encuesta @UK_CEH (39,6k seguidores)

13 de septiembre: CEH tuiteó sobre la encuesta @UK_CEH

- Universidades

1 de septiembre: Se seleccionaron y enviaron correos electrónicos a 10 universidades para pedirles que difundieran la encuesta internamente. Utilizando los rankings de 2022 de 'The Complete University Guide' para Ciencias Biológicas (que incluye, pero no se limita a: Ciencias Biológicas, Biología, Ecología, Biología Marina, Biología Celular, Microbiología, Ciencias Vegetales, Zoología, Genética, Bioquímica, Biología Aplicada, Evolución), se seleccionó una de cada 10 instituciones entre las 100 mejores universidades:

#1 Universidad de Cambridge, Facultad de Ciencias Biológicas

#10 Universidad de Glasgow, Facultad de Ciencias de la Vida

#20 Universidad de Leed

#20 Universidad de Leeds, Facultad de Ciencias Biológicas

#29 University of Nottingham, School of Life Sciences

#39 University of Kent, Durrell Institute of Conservation and Ecology

#49 University of Plymouth, School of Biological and Marine Sciences

#60 Universidad de Lincoln, Escuela de Ciencias de la Vida

#70 Universidad de Northampton

#80 Universidad John Moores de Liverpool, Facultad de Ciencias Biológicas y Medioambientales

#90 Universidad de Derby, Escuela de Medio Ambiente Construido y Natural

13 de septiembre: Envío de un recordatorio a todos los departamentos universitarios.

5 de octubre: Envío de un recordatorio a todos los departamentos universitarios.

5 de octubre: Contacto con otras 10 universidades:

#2 = Universidad de Oxford

#11 = Universidad de Bristol

#21 = Universidad de Bath

#31 = Universidad de Swansea

#41 = Universidad Napier de Edimburgo

#51 = Universidad de Essex

#61 = Universidad de Aberystwyth

#72 = Bangor University (#71 University of Westminster no fue seleccionada por no ser apropiada)

#81 = Universidad de Brighton

#91 = Universidad de Suffolk

Limitaciones

Las limitaciones de nuestra encuesta incluyen: (i) el tamaño relativamente pequeño de la muestra, (ii) el posible sesgo en el reclutamiento de participantes, y (iii) las dificultades para estimar la duración de las actividades científicas en distintos idiomas.

A pesar del considerable esfuerzo que realizamos para distribuir la encuesta en 71 universidades, 12 institutos y 23 sociedades, en tres listas de correo y entre 497 investigadores individuales de ocho países, el tamaño de la muestra de este estudio (908, con un rango de 67 a 292 por idioma) no es necesariamente grande. Esto puede haber causado la falta de potencia en nuestros análisis, lo que podría explicar el efecto no significativo del nivel de ingresos en algunos análisis.

A pesar de que intentamos reclutar a los participantes en la encuesta de la forma más imparcial posible (véase Distribución de la encuesta), reconocemos que es probable que los participantes reclutados representen muestras no aleatorias de toda la población elegible. Por ejemplo, lo más probable es que los participantes en la encuesta sean investigadores activos, por lo que es probable que la encuesta excluya a aquellos que ya han abandonado su carrera investigadora debido a las barreras lingüísticas. Nuestra encuesta también excluye a quienes nunca han publicado un primer artículo en inglés. Esto podría llevar a una subestimación de la gravedad real de las barreras lingüísticas experimentadas por toda la población de hablantes no nativos de inglés. También registramos cinco posible covariables que pueden afectar a la cantidad de esfuerzo necesario para llevar a cabo actividades científicas en inglés: la edad, el sexo, la disciplina, el número de años dedicados a la investigación y el número de publicaciones en inglés. La edad, el sexo, la disciplina y el número de años de investigación estaban correlacionados con el número de publicaciones en inglés (para más detalles, véase Análisis). Por lo tanto, utilizamos el número de publicaciones en inglés como covariable en todos los análisis, para tener en cuenta el efecto de estas covariables.

Hay que reconocer que a los participantes les ha resultado difícil calcular el tiempo exacto que tardan o tardarían en escribir un artículo, leer un artículo o preparar una presentación oral en inglés y en sus lenguas maternas. Para que los participantes pudieran hacer una estimación lo más exacta posible, les preguntamos el tiempo que habrían tardado, por ejemplo, en escribir el último trabajo que hayan escrito en inglés, en lugar del tiempo que pensaban que se necesitaría para escribir un trabajo imaginario, ya que normalmente es más fácil y más exacto contar la experiencia más reciente (sesgo de recuerdo, véase, por ejemplo, [42]). No hay motivos para creer que los hablantes no nativos de inglés sobrestimen sistemáticamente el tiempo real que han dedicado a actividades científicas. Más bien, esperamos que la dificultad para estimar la duración del tiempo empleado en realizar actividades científicas pueda afectar a la precisión, como se refleja en la gran variación dentro de cada grupo de las combinaciones de dominio del inglés-nivel económico. Dado que pedimos a los participantes que respondieran basándose en sus experiencias reales, el tiempo empleado para, por ejemplo escribir un artículo también habría dependido de la longitud del artículo. No obstante, tampoco en este caso hay motivos para creer que los trabajos escritos por hablantes de inglés no nativos sean sistemáticamente más largos que los escritos por hablantes de inglés nativos. Por tanto, no creemos que estas cuestiones afecten a la conclusión principal de este estudio. Dicho esto, la duración de las actividades científicas en la lengua materna no se basa en la experiencia real de los participantes, por lo que debe interpretarse con cautela.

Análisis

En los análisis, sólo utilizamos datos de participantes cuya nacionalidad era una de las ocho nacionalidades objetivo y cuya primera lengua era una de las seis lenguas objetivo. En todos los análisis nos propusimos comprobar si la cantidad de esfuerzo requerido para las actividades científicas, o la frecuencia con la que los participantes se enfrentan a barreras lingüísticas en la ciencia, difiere en función del nivel de dominio del inglés y de la economía de su país de origen, teniendo en cuenta al mismo tiempo el efecto de las covariables.

Como covariables, consideramos las cinco variables siguientes: edad, sexo, disciplina, número de años de investigación y número de publicaciones en inglés. En primer lugar, se comprobaron las correlaciones entre las cinco covariables. La edad y el número de años de investigación estaban muy correlacionados con el número de publicaciones en inglés (coeficiente de correlación de Spearman = 0,58 para la edad y 0,64 para el número de años de investigación). También hubo una relación muy significativa entre el género y el número de publicaciones en inglés (Kruskal-Wallis chi-cuadrado = 68,37, p < 1,42 × 10-15) y entre las disciplinas y el número de publicaciones en inglés (Kruskal-Wallis chi-cuadrado = 29,45, p < 6,35 × 10-6). Así pues, decidimos utilizar únicamente el número de publicaciones en inglés como covariable en los siguientes análisis.

Utilizamos tres tipos de modelos en función del tipo de las variables de respuesta.

Modelos lineales generalizados con una distribución binomial negativa para:

- El número de minutos que se tardó en leer y comprender el último artículo original en inglés que leyó cada participante en su campo.
- El número de minutos que tardarían en leer y comprender el mismo artículo pero en su lengua materna.
- El número de días que se tarda en escribir el primer borrador del último artículo original en inglés de cada participante.
- Número de días que cada participante habría tardado en redactar el primer borrador de su último trabajo en su lengua materna.
- Número de horas necesarias para preparar y practicar una presentación oral en inglés.
- Número de horas necesarias para preparar y practicar la misma presentación oral en su lengua materna.

Modelos lineales generalizados con una distribución binomial para:

- El porcentaje de trabajos en los que la redacción en inglés fue revisada por alguien como favor o por un servicio de pago.
- Porcentaje de trabajos en los que la redacción en inglés fue revisada por alguien a modo de favor.
- Porcentaje de trabajos cuya redacción en inglés fue revisada por un servicio de pago.
- La experiencia de haber tenido un manuscrito escrito en inglés rechazado debido a la redacción en inglés.
- La experiencia de proporcionar un resumen en lengua no inglesa de trabajos en lengua inglesa.
- La experiencia de llevar a cabo la difusión de artículos en inglés en otro(s) idioma(s) además del inglés.

Modelos de enlaces acumulativos para:

- La frecuencia con la que se solicita mejorar la redacción en inglés en la revisión de los primeros trabajos en inglés.
- Frecuencia con la que no se puede explicar con seguridad la propia investigación durante una presentación debido a barreras lingüísticas en inglés.

En todos los modelos se utilizaron tres variables explicativas: el nivel de inglés del país (nativo de inglés como categoría de referencia, moderado (categoría de referencia en los análisis que no incluyen a los nativos de inglés) y bajo), el nivel de ingresos del país (alto como categoría de referencia y medio-bajo) y el número de publicaciones en inglés, así como dos interacciones: dominio de la lengua inglesa y número de publicaciones en inglés, y nivel de renta y número de publicaciones en inglés. En primer lugar, se comprobó si las dos interacciones eran significativas mediante la prueba de la razón de verosimilitud y se excluyeron las interacciones no significativas. Si se excluía alguna interacción, volvíamos a comprobar si las variables explicativas que intervenían en la(s) interacción(es) eran significativas mediante el test likelihood-ratio, y excluimos las variables no significativas para determinar el modelo final. Interpretamos los resultados derivados de los modelos finales. Sin embargo, en algunos análisis (mostrados en las Tablas S3, S15 y S16) incluso las variables no significativas se mantuvieron en los modelos finales para permitir comparaciones con los resultados de otros análisis asociados.

Todos los análisis se realizaron en R versión 4.1.2 [43]. También utilizamos los siguientes paquetes de R: *tidyverse* [44], *MASS* [45], *lmtest* [46], *janitor* [47], *corrplot* [48], *ordinal* [49], y *gridExtra* [50].

**Agradecimientos**

Damos las gracias a todos los participantes en nuestra encuesta y a M. Amano por la corrección del inglés.

**Referencias**

1. Hong L, Page SE. Groups of diverse problem solvers can outperform groups of high-ability problem solvers. Proceedings of the National Academy of Sciences. 2004;101(46):16385-9. doi: 10.1073/pnas.0403723101.

2. Hofstra B, Kulkarni VV, Galvez SM-N, He B, Jurafsky D, McFarland DA. The diversity-innovation paradox in science. Proceedings of the National Academy of Sciences. 2020;117(17):9284-91. doi: 10.1073/pnas.1915378117.

3. Alshebli BK, Rahwan T, Woon WL. The preeminence of ethnic diversity in scientific collaboration. Nature Communications. 2018;9(1):5163. doi: 10.1038/s41467-018-07634-8.

4. Amano T, Berdejo-Espinola V, Christie AP, Willott K, Akasaka M, Báldi A, et al. Tapping into non-English-language science for the conservation of global biodiversity. PLOS Biology. 2021;19(10):e3001296. doi: 10.1371/journal.pbio.3001296.

5. Tengö M, Hill R, Malmer P, Raymond CM, Spierenburg M, Danielsen F, et al. Weaving knowledge systems in IPBES, CBD and beyond—lessons learned for sustainability. Current Opinion in Environmental Sustainability. 2017;26-27:17-25. doi: 10.1016/j.cosust.2016.12.005.

6. Pearson AR, Schuldt JP. Facing the diversity crisis in climate science. Nature Climate Change. 2014;4(12):1039-42. doi: 10.1038/nclimate2415.

7. Trisos CH, Auerbach J, Katti M. Decoloniality and anti-oppressive practices for a more ethical ecology. Nature Ecology & Evolution. 2021;5(9):1205-12. doi: 10.1038/s41559-021-01460-w.

8. Raja NB, Dunne EM, Matiwane A, Khan TM, Nätscher PS, Ghilardi AM, et al. Colonial history and global economics distort our understanding of deep-time biodiversity. Nature Ecology & Evolution. 2022;6(2):145-54. doi: 10.1038/s41559-021-01608-8.

9. Bernard RE, Cooperdock EHG. No progress on diversity in 40 years. Nature Geoscience. 2018;11(5):292-5. doi: 10.1038/s41561-018-0116-6.

10. Montgomery S. Of towers, walls, and fields: Perspectives on language in science. Science. 2004;303(5662):1333-5. doi: 10.1126/science.1095204.

11. Woolston C, Osório J. When English is not your mother tongue. Nature. 2019;570(7760):265-7. doi: 10.1038/d41586-019-01797-0.

12. Pérez Ortega R. Science's English dominance hinders diversity—but the community can work toward change. Science. 2020. doi: 10.1126/science.caredit.abf4697.

13. Montgomery SL. Does science need a global language? Chicago: The University of Chicago Press; 2013.

14. UNESCO. UNESCO recommendation on open science. Paris, France: 2021.

15. Ferguson G, Pérez-Llantada C, Plo R. English as an international language of scientific publication: a study of attitudes. World Englishes. 2011;30(1):41-59. doi: 10.1111/j.1467-971x.2010.01656.x.

16. Hanauer DI, Sheridan CL, Englander K. Linguistic injustice in the writing of research articles in English as a second language: data from Taiwanese and Mexican researchers. Written Communication. 2019;36(1):136-54. doi: 10.1177/0741088318804821.

17. Clavero M. "Awkward wording. Rephrase": linguistic injustice in ecological journals. Trends in Ecology and Evolution. 2010;25(10):552-3. doi: 10.1016/j.tree.2010.07.001.

18. Huttner-Koros A, Perera S. Communicating science in English: a preliminary exploration into the professional self-perceptions of Australian scientists from language backgrounds other than English. Journal of Science Communication. 2016;15(06). doi: 10.22323/2.15060203.

19. Ramírez-Castañeda V. Disadvantages in preparing and publishing scientific papers caused by the dominance of the English language in science: The case of Colombian researchers in biological sciences. PLOS ONE. 2020;15(9):e0238372. doi: 10.1371/journal.pone.0238372.

20. Politzer-Ahles S, Girolamo T, Ghali S. Preliminary evidence of linguistic bias in academic reviewing. Journal of English for Academic Purposes. 2020;47:100895. doi: 10.1016/j.jeap.2020.100895.

21. EF Education First. EF English Proficiency Index. 2020.

22. The World Bank. World Bank list of economies (June 2020). 2020.

23. Joo R, Sánchez-Tapia A, Mortara S, Bellini Saibene Y, Turner H, Hug Peter D, et al. Ten simple rules to host an inclusive conference. PLOS Computational Biology. 2022;18(7):e1010164. doi: 10.1371/journal.pcbi.1010164.

24. Levitis E, Cassandra DG, Gau R, Heunis S, Dupre E, Kiar G, et al. Centering inclusivity in the design of online conferences—An OHBM–Open Science perspective. GigaScience. 2021;10(8). doi: 10.1093/gigascience/giab051.

25. Primack RB, Marrs R. Bias in the review process. Biological Conservation. 2008;141(12):2919-20. doi: 10.1016/j.biocon.2008.09.016.

26. Primack RB, Zipf L. Acceptance rates and number of papers in Biological Conservation from 2005 to 2014 for Australia, Brazil, China, India, Spain, and the United States: Trends or noise? Biological Conservation. 2016;196:50-2. doi: 10.1016/j.biocon.2016.01.016.

27. Smith OM, Davis KL, Pizza RB, Waterman R, Dobson KC, Foster B, et al. Peer review perpetuates barriers for historically excluded groups. Nature Ecology & Evolution. 2023;7:512-23. doi: 10.1038/s41559-023-01999-w.

28. Maas B, Pakeman RJ, Godet L, Smith L, Devictor V, Primack R. Women and Global South strikingly underrepresented among top-publishing ecologists. Conservation Letters. 2021;14(4):e12797. doi: 10.1111/conl.12797.

29. Barath H. Indian initiatives aim to break science's language barrier. Nature. 2019;571:289-90. doi: 10.1038/d41586-019-01815-1.

30. Evans TM, Bira L, Gastelum JB, Weiss LT, Vanderford NL. Evidence for a mental health crisis in graduate education. Nature Biotechnology. 2018;36(3):282-4. doi: 10.1038/nbt.4089.

31. Waruru M. African and Asian researchers are hampered by visa problems. Nature. 2018;10:d4158. doi: 10.1038/d41586-018-06750-1.

32. Convention on Biological Diversity. Kunming-Montreal Global biodiversity framework, Draft decision submitted by the President. 2022.

33. Amano T, Berdejo-Espinola V, Akasaka M, de Andrade Junior MAU, Blaise N, Checco J, et al. The role of non-English-language science in informing national biodiversity assessments. Nature Sustainability. 2023. doi: 10.1038/s41893-023-01087-8.

34. Berdejo-Espinola V, Amano T. AI tools can improve equity in science. Science. 2023;379(6636):991. doi:10.1126/science.adg9714.

35. Steigerwald E, Ramírez-Castañeda V, Brandt DYC, Báldi A, Shapiro JT, Bowker L, et al. Overcoming Language Barriers in Academia: Machine Translation Tools and a Vision for a Multilingual Future. BioScience. 2022;72:988-98. doi: 10.1093/biosci/biac062.

36. Thorp HH. ChatGPT is fun, but not an author. Science. 2023;379(6630):313. doi:10.1126/science.adg7879.

37. van Dis EAM, Bollen J, Zuidema W, van Rooij R, Bockting CL. ChatGPT: five priorities for research. Nature. 2023;614:224-6. doi: 10.1038/d41586-023-00288-7.

38. Ammon U. Linguistic inequality and its effects on participation in scientific discourse and on global knowledge accumulation – With a closer look at the problems of the second-rank language communities. Applied Linguistics Review. 2012;3(2):333-55. doi:10.1515/applirev-2012-0016.

39. Amano T, Rios Rojas C, Boum II Y, Calvo M, Misra BB. Ten tips for overcoming language barriers in science. Nature Human Behaviour. 2021;5:1119–22. doi: 10.1038/s41562-021-01137-1.

40. Lynch AJ, Fernández-Llamazares Á, Palomo I, Jaureguiberry P, Amano T, Basher Z, et al. Culturally diverse expert teams have yet to bring comprehensive linguistic diversity to intergovernmental ecosystem assessments. One Earth. 2021;4(2):269-78. doi: 10.1016/j.oneear.2021.01.002.

41. Eberhard DM, Simons GF, Fennig CD, editors. Ethnologue: Languages of the World. Twenty-fifth edition. Dallas, Texas: SIL International. Online version: http://www.ethnologue.com. 2022.

42. Khare SR, Vedel I. Recall bias and reduction measures: an example in primary health care service utilization. Family Practice. 2019;36(5):672-6. doi: 10.1093/fampra/cmz042.

43. R Core Team. R: A language and environment for statistical computing. Vienna, Austria. Available at: https://www.R-project.org/: R Foundation for Statistical Computing; 2021.

44. Wickham H, Averick M, Bryan J, Chang W, McGowan LDA, François R, et al. Welcome to the tidyverse. Journal of Open Source Software. 2019;4(43):1686. doi: 10.21105/joss.01686.

45. Ripley B, Venables B, Hornik K, Gebhardt A, Firth D. MASS: support functions and datasets for Venables and Ripley's MASS: Available at: http://cran.r-project.org/web/packages/MASS/index.html; 2013.

46. Hothorn T, Zeileis A, Farebrother RW, Cummins C, Millo G, Mitchell D. lmtest: Testing linear regression models. R package version 0.9-40.: https://cran.r-project.org/web/packages/lmtest/index.html; 2022.

47. Firke S, Denney B, Haid C, Knight R, Grosser M, Zadra J. janitor: Simple tools for examining and cleaning dirty data. R package version 2.1.0.: https://cran.r-project.org/web/packages/janitor/index.html; 2021.

48. Wei T, Simko V, Levy M, Xie Y, Jin Y, Zemla J, et al. corrplot: Visualization of a correlation matrix. R package version 0.92.: https://cran.r-project.org/web/packages/corrplot/index.html; 2021.

49. Christensen RHB. Package 'ordinal': Regression Models for Ordinal Data: Available at: https://cran.r-project.org/web/packages/ordinal/; 2015.

50. Auguie B. gridExtra: miscellaneous functions for "grid" graphics. R package version 2.3. https://CRAN.R-project.org/package=gridExtra2017.

51. Van Noorden R. Scientists may be reaching a peak in reading habits. Nature. 2014. doi: 10.1038/nature.2014.14658.

**Leyendas de los archivos de información complementaria**

Información complementaria: incluye las Tablas complementarias S1-S17, las Figuras complementarias. S1-S9 y el texto suplementario S1.

Datos S1: incluye los datos subyacentes a las Figs. 2B, 3A, 3B, 4C, S1, S2, S4, S5, S6, S7, S8 y S9.

**Financiación**

Beca Future del Consejo Australiano de Investigación FT180100354 (TA)

Financiación estratégica de la Universidad de Queensland (TA)

Fundación Alemana de Investigación (DFG-FZT 118, 202548816) (SC)

**Contribución de los autores**

Conceptualización: TA, VR-C, DV

Análisis formal: TA

Obtención de fondos: TA, SC

Investigación: TA, VB-E, IB, SC, MG, JDG-T, FM-C, KP, RW

Metodología: TA, VR-C, VB-E, DV

Administración del proyecto: TA, VB-E

Validación: TA, VB-E

Visualización: TA

Redacción - borrador original: TA

Redacción - revisión y edición: TA, VR-C, VB-E, IB, SC, MG, JDG-T, FM-C, KP, RW, DV

**Conflicto de intereses**

Los autores declaran no tener intereses contrapuestos.

**Disponibilidad de datos y materiales**

Los datos subyacentes a las Figs. 2B, 3A, 3B, 4C, S1, S2, S4, S5, S6, S7, S8 y S9 pueden encontrarse en Datos S1. Los datos subyacentes a las Figs. 1A, 1B, 1C, 1D, 1E, 1F, 2A, 2C, 2D, 4A, 4B y S3 son datos brutos obtenidos directamente de las preguntas de la encuesta, que nuestra aprobación ética nos impide compartir para garantizar la confidencialidad de los encuestados. El acceso a estos datos brutos debe solicitarse a la oficina de Ética de la Universidad de Queensland, con la que se puede contactar en humanethics@research.uq.edu.au. Todos los códigos utilizados en el análisis están disponibles en: http://doi.org/10.17605/OSF.IO/Y94ZT.
