# Supplementary material for: The manifold costs of being a non-native English speaker in science
Source: PLoS Biol. 2023 Jul 18;21(7):e3002184. doi: 10.1371/journal.pbio.3002184 (PMC10353817; doi:10.1371/journal.pbio.3002184)
Supplement: S5 Text — (DOCX) [file pbio.3002184.s032.docx]

**Los múltiples costos de ser un angloparlante no nativo en la ciencia**

Resumen

El inglés como lengua franca de la ciencia representa un gran obstáculo para la participación de personas que no son hablantes nativas de este idioma. Aun así, no existe en la actualidad una estimación de los costos que imponen las barreras impuestas por el inglés en el desarrollo profesional de investigadores e investigadoras. En este estudio, se estimó, mediante una encuesta a 908 investigadores en ciencias ambientales, la cantidad de esfuerzo requerido para desarrollar actividades científicas en inglés. Luego se compararon los resultados entre investigadores e investigadoras de diferentes países y, por lo tanto, de diferentes antecedentes lingüísticos y económicos, incluyendo participantes angloparlantes nativos. Los resultados de la encuesta revelan que, sin importar el país, los y las angloparlantes no nativos dedican más esfuerzo que los y las angloparlantes nativos en la realización de actividades científicas que incluyen leer y escribir artículos, y preparar presentaciones o pósters para difundir su investigación en congresos. Esta brecha, que es especialmente amplía al comienzo de la carrera científica, puede llevar a que investigadores e investigadoras desistan de participar en conferencias internacionales realizadas en inglés. Debido a que estas brechas pueden impedir la difusión internacional del conocimiento, invitamos a las comunidades científicas a reconocer y abordar estas desventajas para liberar el potencial no aprovechado de los angloparlantes no nativos. Individuos, instituciones, revistas, financiadores y conferencias científicas pueden contribuir a la solución de esta problemática global.


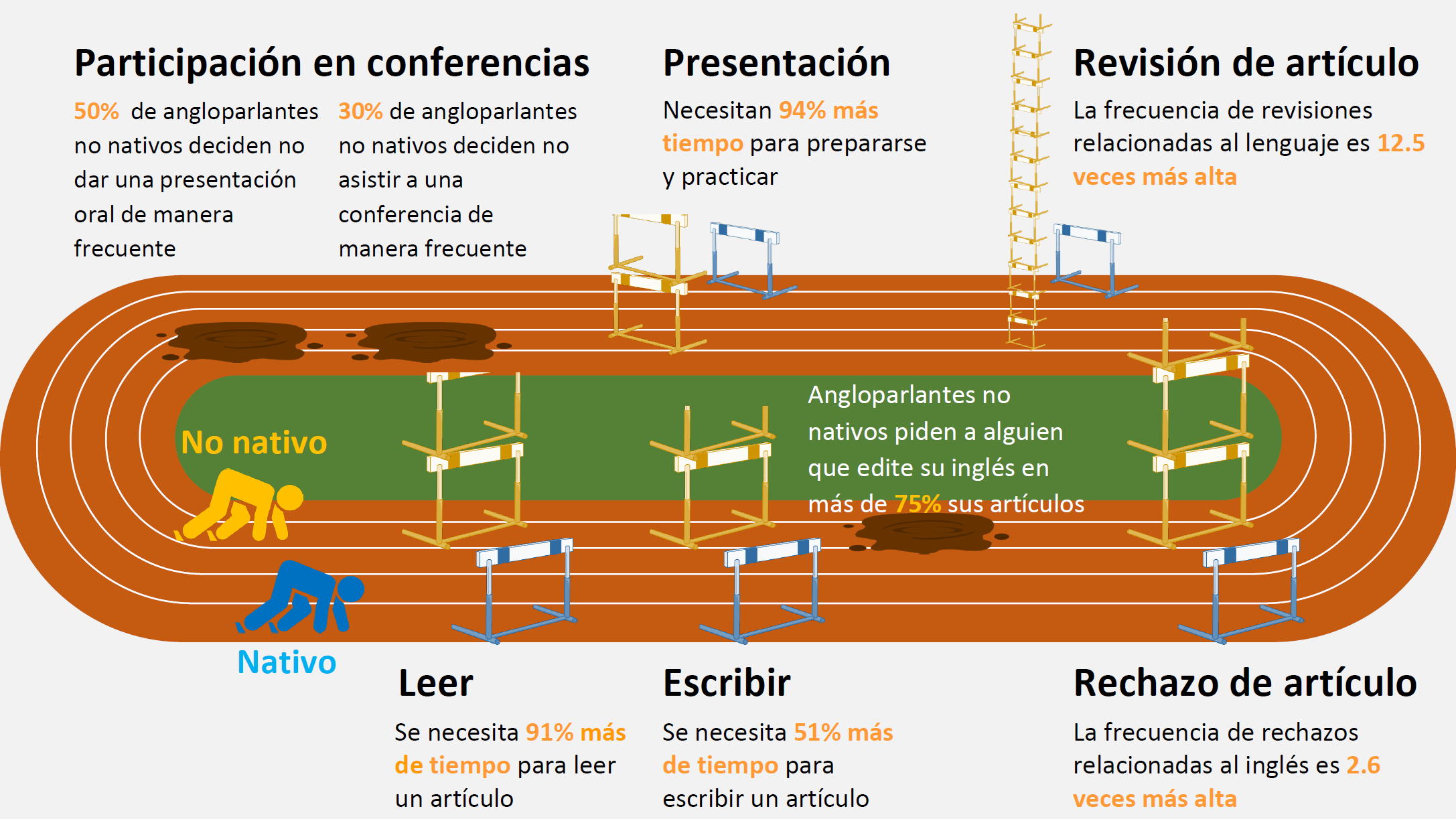


**Fig 5**. Desventajas estimadas de angloparlantes no nativos en la realización de diferentes actividades científicas. La altura de los obstáculos permite comparar el esfuerzo realizado por angloparlantes no nativos en términos del tiempo necesario para leer (Lectura), (Escritura) y preparar una presentación oral en inglés (Presentación), y la frecuencia relativa con que un artículo en inglés fue rechazado (Rechazo de artículo) o se ha hecho la solicitud de reevaluar la escritura del inglés (Revisión de artículo). Los valores corresponden a hablantes no nativos de inglés que solo han publicado un artículo en ese idioma (se tomó el valor más alto entre las nacionalidades con competencia en inglés moderada y baja), en comparación con los valores para los hablantes nativos de inglés. La analogía usada en la figura no pretende hacer una apología a la ciencia como una competencia.


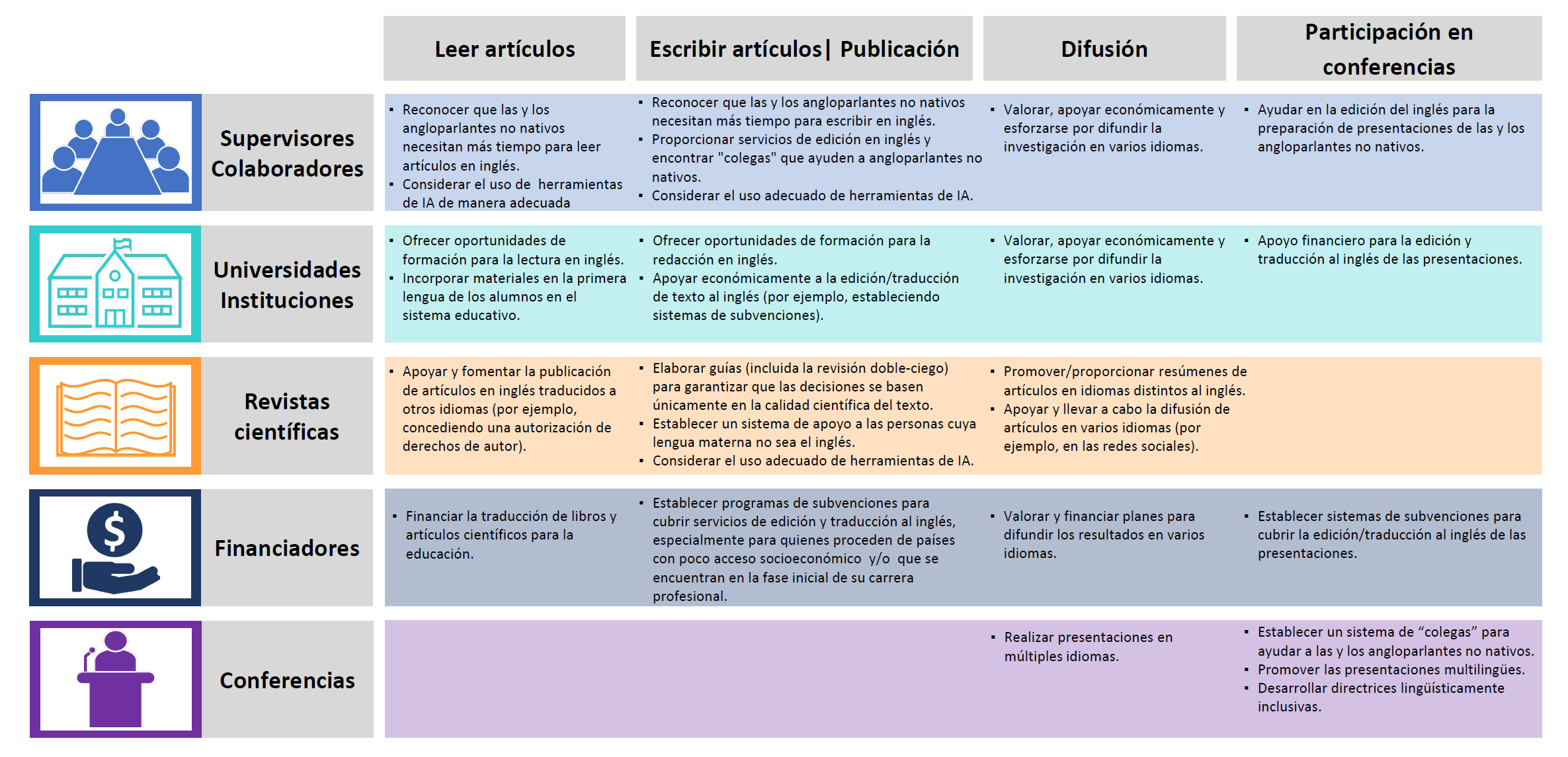


**Fig 6**. Ejemplos de posibles soluciones para reducir las desventajas de los angloparlantes no nativos en cada tipo de actividad científica. IA: inteligencia artificial. También consulte [35, 38, 39] para otras posibles soluciones.
