# Supplementary material for: The manifold costs of being a non-native English speaker in science
Source: PLoS Biol. 2023 Jul 18;21(7):e3002184. doi: 10.1371/journal.pbio.3002184 (PMC10353817; doi:10.1371/journal.pbio.3002184)
Supplement: S6 Text — (DOCX) [file pbio.3002184.s033.docx]

**Багатоманіття зусиль для неносіїв англійської мови в науці**

**Анотація**

Використання англійської мови як основної мови науки є головною перешкодою для максимізації внеску в науку неносіїв англійської мови. Проте лише кілька досліджень кількісно оцінили наслідки мовних бар'єрів для кар'єрного розвитку тих, для кого англійська мова не є рідною. На основі опитування 908 дослідників у галузі наук про навколишнє середовище, це дослідження оцінює та порівнює кількість зусиль, необхідних для ведення наукової діяльності англійською мовою, між дослідниками з різних країн, а отже, з різним мовним та економічним походженням. Наше дослідження показує, що дослідники, для яких англійська мова не є рідною, особливо на початку кар'єри, витрачають більше зусиль, ніж носії англійської мови, на ведення наукової діяльності – від читання і написання статей та підготовки презентацій англійською мовою до поширення результатів досліджень кількома мовами. Мовні бар'єри також можуть стати причиною того, що дослідники не відвідують англомовні міжнародні конференції або не виступають на них з усними доповідями. Ми закликаємо наукові спільноти визнати і подолати ці перешкоди, щоб вивільнити невикористаний потенціал науковців, для яких англійська мова не є рідною. Ми також пропонуємо потенційні рішення, які можуть бути реалізовані вже сьогодні окремими особами, установами, журналами, спонсорами та конференціями.


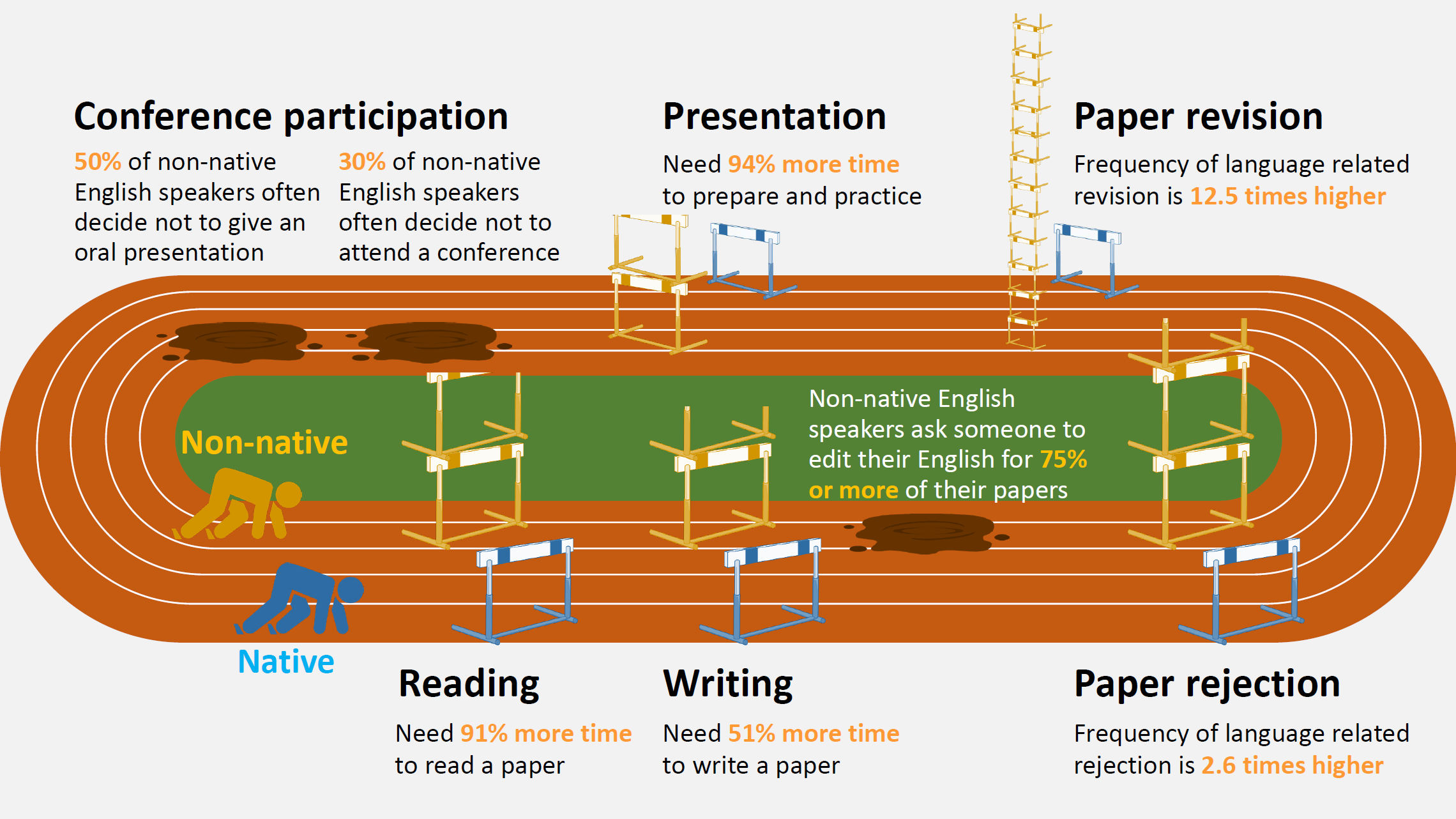


**Рис. 5. Перешкоди для неносіїв англійської мови при здійсненні різних видів наукової діяльності.** Висота бар'єрів вказує на відносну тривалість часу, необхідного для читання англомовної статті (Reading), написання статті англійською мовою (Writing) та підготовки усної презентації англійською мовою (Presentation), а також на відносну частоту відхилення англомовної статті (Paper rejection) або повернення її на доопрацювання (Paper revision) через те, що вона написана англійською мовою, для осіб, які не є носіями англійської мови (Non-native), порівняно з особами, які є носіями англійської мови (Native). Значення наведені для неносіїв англійської мови, які опублікували лише одну англомовну статтю (вищі значення для національностей з середнім та низьким рівнем володіння англійською мовою), порівняно зі значеннями для носіїв англійської мови. Цей рисунок не стверджує, що наука – це змагання.


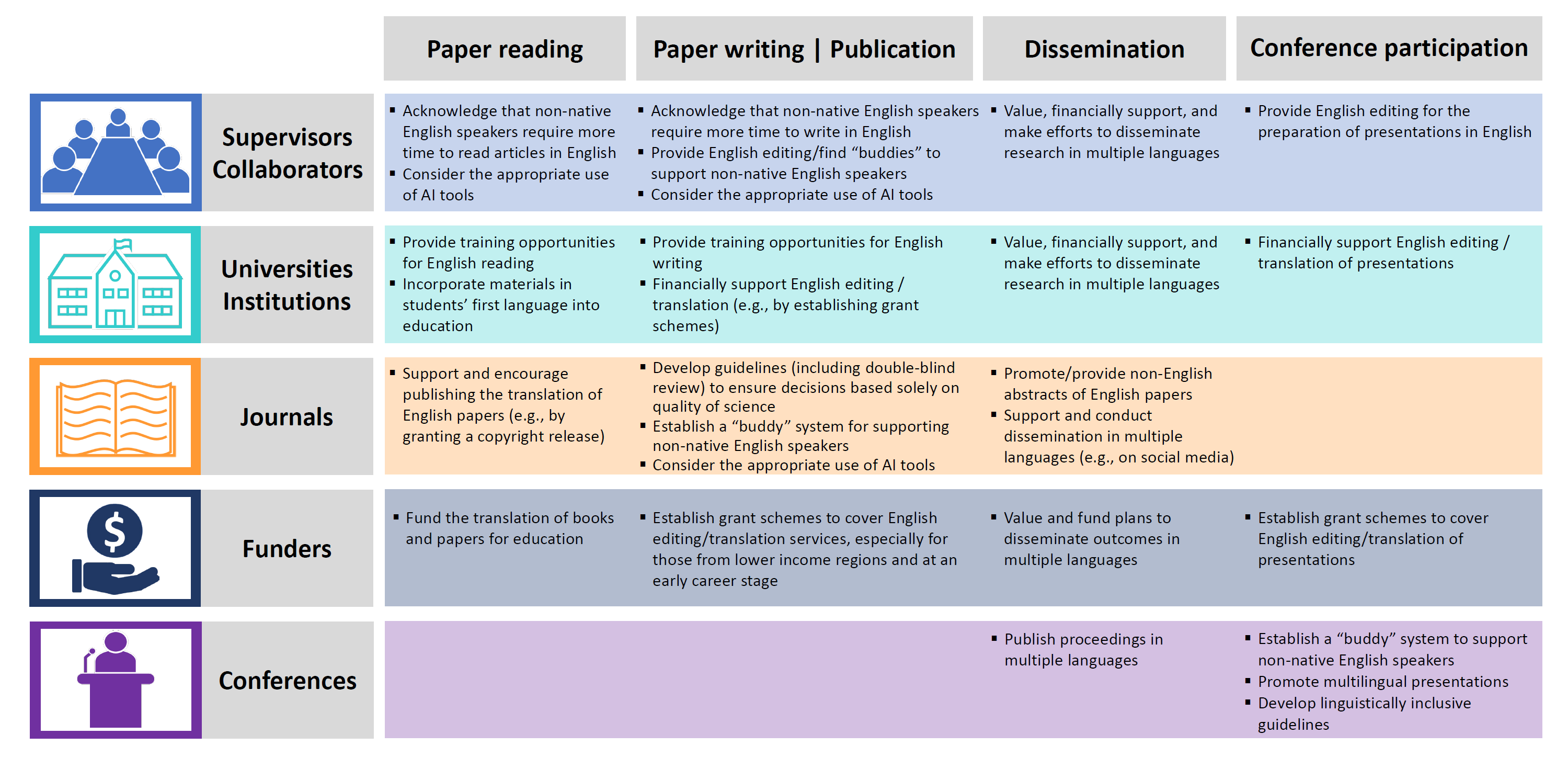


**Рис. 6. Приклади потенційних рішень для зменшення перешкод для неносіїв англійської мови в різних видах наукової діяльності.** ШІ: штучний інтелект. Також див. [35, 38, 39] щодо інших потенційних рішень.
