# Supplementary material for: The manifold costs of being a non-native English speaker in science
Source: PLoS Biol. 2023 Jul 18;21(7):e3002184. doi: 10.1371/journal.pbio.3002184 (PMC10353817; doi:10.1371/journal.pbio.3002184)
Supplement: Attachment — Submitted filename: Responses.docx [file pbio.3002184.s034.docx]

**Response to the reviewers for "** **The cost of being a non-native English speaker in science" (PBIOLOGY-D-22-02711R1)**

---------------------------------------------------------------------------------------------------------------------------

**In response to Editor’s comments**

Comment #1

Thank you for your patience while your manuscript "The cost of being a non-native English speaker in science" went through peer-review at PLOS Biology. Your manuscript has now been evaluated by the PLOS Biology editors, an Academic Editor with relevant expertise, and by three independent reviewers. Please accept my apologies for the delay incurred over the holiday period during the early stages of the editorial process.

You’ll see that all three reviewers are positive about your study, but each raises a number of issues that should be addressed before further consideration. For example, reviewer #1 wants you to spell out the limitation that it focuses on fields that may be atypical in terms of collaboration patterns, reviewer #2 raises a number of interesting issues that can probably be addressed textually, and reviewer #3 suggests that you make better use of your Figs by binning all the “>50 papers” data points together, thereby presenting the bulk of the data at higher resolution. In addition, two reviewers request that you incorporate more extensive discussion of potential solutions to the problems identified; both suggest moving Table S18 into the main paper.

Thank you very much for your positive evaluation. We have accepted or addressed the suggestions provided by the three reviewers, and made corrections accordingly. In particular, we have expanded the section on limitations in the Discussion following the suggestion by Reviewer #1, converted what was originally Table S18 into a new infographic Fig. 6 to extensively discuss potential solutions in the main text, as suggested by Reviewers #1 and 3, and improved the clarity of Fig. 1, as suggested by Reviewer #3. We have also replaced the original Fig. S6 with a new infographic Fig. 5 to make it much easier to visually understand the multiple disadvantages of non-native English speakers.

There are nevertheless a few comments where, after careful thought, we have decided to maintain the original version. Each of these points is listed in our responses to the reviewers with a full explanation of our reasoning. We hope that the Editor will find our responses satisfactory, but should any concerns remain, we will be more than happy to discuss further.

Comment #2

In light of the reviews, which you will find at the end of this email, we are pleased to offer you the opportunity to address the comments from the reviewers in a revision that we anticipate should not take you very long. We will then assess your revised manuscript and your response to the reviewers' comments with our Academic Editor aiming to avoid further rounds of peer-review, although might need to consult with the reviewers, depending on the nature of the revisions.

Thank you for this too. We appreciate that the Editor has granted an extension to the resubmission deadline.

**In response to Reviewer #1’s comments**

Comment #1

This is the review of "The cost of being a non-native English speaker in science" by Tatsuya Amano and colleagues. This tackles a very important issue that is a major impediment to the effectiveness and productivity of scientific systems, holding back global participation in the research process.

I am reviewing this as someone working in the publishing industry for 15 years, and an Open Science advocate with experience and a perspective living in non-English speaking countries (E and SE Asia) for most of my career. I do not have the statistical expertise to assess those parts, and have also not carried out survey-based research, so hope that other referees are able to assess these areas sufficiently.

Thank you so much for this extremely helpful review and positive evaluation. We have carefully considered all the comments by Reviewer #1 and revised the manuscript accordingly. We hope that Reviewer #1 will find our responses satisfactory, but should any concerns remain, we will be more than happy to discuss further.

Comment #2

A strength of the study is that it is trying to look purely at the cost of the language barriers, ignoring many of the other geographic biases in science that may exist. Although these are hard confounders to remove. For example as conferences and conference papers are covered here, many of the nationalities covered will have financial and immigration issues attending conferences in North America and Europe that are arguably much more challenging and stressful than just the language issues. And even online conferences may not be completely immune to some of these inclusivity issues (see and overview here <https://doi.org/10.1093/gigascience/giab051)>. So one potential challenge for this study is whether all of these bigger picture societal issues have been properly taken into account in the study design and discussion.

This is a very good point and we are fully aware of the potential confounding between language barriers and other types of barriers, especially when quantifying the effort needed to do some of the scientific activities we investigated (e.g., barriers to participation in conferences).

In terms of the study design adopted, we tried our best to isolate the effect of language barriers from that of other barries in two ways. First, we stratified the nationalities of focus by the level of English proficiency and income; this way we were able to attribute any difference in results between nationalities with the same level of English proficiency but different income levels to the effect of income level. For increased clarity, we have added this explanation in L89-91. Second, our survey is designed to explicitly ask participants about their experiences in struggling to conduct each scientific activity due to language barriers, e.g., see the part highlighted in bold: “*6.2 How often have you decided not to attend an English-language conference (either for presenting your research or just for participating)* ***because you were not confident enough to communicate in English?***” (also see all other questions in Supplementary Text S1). We have also emphasised this in L373-378.

Having said that, we agree with Reviewer #1 that we still cannot dismiss the possibility that the cost for non-native English speakers in science quantified in this study *partly* (also note that these other barriers are not perfectly correlated with language barriers) incorporates the cost associated with other types of barriers. Therefore, we also included this caveat in the Discussion (L268-274). We have also cited three additional references (Refs #23, 24, and 31) including the one suggested by Reviewer #1 here to support the argument.

Comment #3

The topic in question - quantifying costs and drawbacks of English being the common language of science is extremely timely and topical with nearly 200 member states ratifying in late-2021 the UNESCO Recommendation of Open Science. Making a commitment to apply the provisions of these by taking whatever legislative or other measures may be required to give effect within their jurisdictions. UNESCO spend a lot of time in the Recommendations outlining the core values and guiding principles of Open Science, and among these "Equity and fairness", "Diversity and inclusiveness", "Equality of opportunities", and "Collaboration, participation and inclusion" all highlight language as a key issue.

The UNESCO report ends with areas of action and 8 specific recommendations, one of these being "Encouraging multilingualism in the practice of science, in scientific publications and in academic communications." To address such important changes data is required, and member states are also asked to monitor uptake and the benefits of open science as a result of the implementation of these recommendations. Providing quantitative data with regards to one of the key areas highlighted is therefore a useful outcome of this work, and it would be useful to highlight that topicality in the introduction and discussion.

See: UNESCO Recommendation on Open Science, November 2021. <https://unesdoc.unesco.org/ark:/48223/pf0000379949.locale=en>

This is an extremely helpful suggestion, thank you very much. It is great to see that the UNESCO explicitly recognises the importance of solving this issue. As suggested, we have referred to the report in the Introduction (L58-62) and Discussion (L287-290) to highlight the topicality of the issue.

Comment #4

A major limitation not highlighted sufficiently is the fact that this is being sold as a study quantifying these issues in all of science, and when looking at the methodology and sample collected this data is very specific to a few particular areas of science. Namely conservation, ecology and evolutionary biology. While this fits with the "Biology" scope of PLOS Biology, this major limitation definitely needs to be made much clearer at the start of the introduction, probably in the abstract, and maybe even the title. The language issue is likely to be relatively generalisable, but without more data it is impossible to say if these figures are the same across all fields of science. Where some fields may be more theoretical, mathematical or computational. With more lab-based (financial) resource heavy fields skewed to researchers with bigger international collaboration networks providing them better access to expensive equipment and reagent, and English speaking co-authors and
collaborators to ask favours from. Or if there are fields with a longer history of undergraduate teaching using English language textbooks, or that are so fast moving only English language teaching resources exist.

We agree with Reviewer #1 that the level of language barriers could vary among disciplines and we may not be able to generalise our findings across disciplines. Following the suggestion, we have now clearly stated that our focus is on environmental sciences in the Abstract (L31-33) and also discussed the possibility that our findings may not be quantitatively applicable to all disciplines (L274-278).

Please note that the Introduction already mentions that we surveyed researchers in environmental sciences (L78). We have now added an explanation that we particularly focused on ecology, evolutionary biology, conservation biology, and related disciplines (L78-79).

Comment #5

Where these types of meta-research studies can be very successful is where they don't just quantify a problem, but also propose some potential solutions too. See the Ioannidis PLOS Medicine paper "How to Make More Published Research True" as an example of this (<https://doi.org/10.1371/journal.pmed.1001747>). In the case of this work, there is little to no discussion on this in the main paper, and the main mention of this is hidden in the supplementary files table S18. I would strongly make the case to make this more prominent as a table or infographic in the discussion section of the main paper. Especially as S18 isn't supplementary data, it is an important part of the narrative.

Thanks very much for this helpful comment too. We totally agree and have now re-written the whole paragraph about potential solutions (L279-298). We have also converted Table S18 into infographic and now present it as Fig. 6 in the main text. We hope this has successfully highlighted the solution part, as Reviewer #1 suggested here.

Please also note that we have replaced what was previously Fig. S6 with a new Fig. 5 – another infographic that visualises the estimated multiple disadvantages for non-native English speakers.

Comment #6

Some other minor points that could be useful to clarify and think about.

Looking at figure 1 charts on Language barriers in paper reading and writing, it looks like the total number of English-language papers published is less for the non-native English speaking authors and that career limitation is sad in itself. In Fig 1A it looks like for readers with moderate English-proficiency, once they get beyond a certain level of seniority they are slightly faster at reading English papers than native English readers. Is that just an artifact or can it have another explanation?

It certainly appears that survey participants with low and moderate English proficiency nationalities published less than native-English-speaking participants (mean number of English-language publications: 18.4 for native English speakers; 12.0 for those with moderate English proficiency nationalities; 9.8 for those with low English proficiency nationalities). However, the number of publications can be affected by many factors that were not surveyed in this study (note that our survey was unfortunately not designed to compare the number of publications), so we have decided not to compare and discuss the number of publications between nationalities. But we have briefly mentioned that the disadvantages could lead to the severe under-representation of research by non-native English speakers in the Discussion (L238-239).

In terms of the result that non-native English speakers with a certain level of seniority seem to spend less time than native English speakers for reading/writing English papers, we suspect this could be due to survivorship bias, i.e., only those non-native English speakers who have managed to conduct science in English as efficiently as native English speakers may have remained in a research career and thus been the dominant group among the experienced researchers who participated in this survey. We had already explained this interpretation in L242-247.

Comment #7

While researchers in low and lower middle income countries will find it hard to afford expensive editing services, I'm curious if you have an idea why this group of researchers also were less frequent in asking someone to help as a favour? Do you feel this is likely because of a lack of access to contacts that can help or another reason?

It is hard to conclude whether those in lower-middle income countries had a significantly smaller proportion of papers checked for English as a favour, compared to those in high income countries. As shown in Fig. 1E and Table S7, the proportion is higher in those in lower-middle income countries when the number of English publications is small, but lower when the number of English publications is large (because the interaction was significant). Therefore we have decided not to discuss this, while we agree that it is generally true for most non-native English speakers not to have access to contacts who can provide English support as a favour.

Comment #8

Machine translation is dismissed as not being relevant due to low usage rate in the countries surveyed. The usefulness of this is likely to vary based on languages that have better trained and working translation tools such as Chinese (and may explain the slightly higher rate in Japanese). With the recent release of ChatGPT AI/NLP guided or enhanced writing is an extremely fast moving and topical area, and while writing created by AI is being prohibited by universities and journals, there has been less discussion on AI-utilising tools that more subtly improve and polish writing such as Grammarly. This was missed off the survey but it would have been interesting to know the uptake of tools such as these in these different research communities. And could potentially be a topic for follow-up.

We agree that emerging AI tools such as ChatGPT have a great potential to reduce language barriers. As we also argued in a recent letter to the journal *Science* (now cited as Ref 34), we believe journals should allow the use of AI tools for English proofreading and have now added this discussion in the paragraph on solutions (L290-298). It would be certainly interesting to survey the update of such tools in different communities in our future work – many thanks for the suggestion!

**In response to Reviewer #2’s comments**

Comment #1

First, thank you for investigating this urgent topic by quantifying the actual cost to career opportunities of translation in research. It is shocking, even for people like me who are already sensitized to the issue in certain disciplines and editorial decision makings - to see numbers to the effect that language proficiency has on career-defining activities in academia. You make a compelling case highlighting the cost of translation to non-native English-speaking researchers from various national backgrounds with varying English proficiency and national income levels as based on international standards.

You contextualize the issue with the urgency for political action re biodiversity and climate change, where we have a dire need for a research-informed global society by tapping into various knowledge systems a.o.

Thanks very much for the positive evaluation. We agree that this is a serious and urgent issue. To further emphasise its urgency and call for immediate actions, and also following some helpful suggestions from other reviewers, we have now included two infographics: Fig. 5 visualises the estimated multiple disadvantages for non-native English speakers (we have replaced what was previously Fig. S6 with this new Fig. 5), and Fig. 6 summarises examples of potential solutions that can be adopted by different sectors. We hope this has further improved our manuscript.

Comment #2

Methodology

The number of individuals successfully recruited for the survey with 40 to 294 per linguistic/economic groups appears adequate to allow for the conclusions drawn, even though you argue for a relatively low sample size yourselves. The translation efforts are well-categorized reflecting the real-world scenarios in engaging in the foreign (secondary, tertiary, …) language for scientific purposes.

With the design of the survey, allowing for adequate self-assessment in responding to the quantifying questions, as well as survey distribution in each of the target countries' official language, you made appropriate efforts to invite for informed participation of a large enough group in each country.

Data anonymization was well taken care of as well as informing participants of the voluntary nature of the survey.

Thanks very much for acknowledging that we had paid extra attention to the design and distribution of the survey.

Comment #3

Discussion

The style chosen in the first paragraph of the discussion is compelling, where the reader is invited to put him/her/them-self/selves into the shoes of a non-native English speaking ECR, which makes the challenge even more immediate and approachable.

You present sound examples and conclusions about biases in the career development of non-native English speakers being selected against based on their English-language proficiency (line 217-220).

You further argue for economic disadvantages being the reason for hindrances in utilizing English language editing services, and what effect the presence or absence of scientific terms and glossaries in certain language group may have on tertiary educational choices amongst the population of a country (line 221-231).

The limitations of your study are coherently addressed with underestimated disadvantages during the planning phase that you became aware of during the survey analysis.

Following suggestions from other reviewers, in the revised version we have further discussed two more limitations of our study: the possibility that (i) the disadvantages we quantified may incorporate those associated with other types of barriers, such as economic and immigration barriers, and (ii) the level of disadvantages could vary among disciplines (L268-278).

Comment #4

Recommendations

Not to ignore research communities that are communicating in their local and other large regional languages - such as Arabic, French, Spanish, Chinese, and Russian, you could perhaps set a scope for your appeal for those disciplines and research communities or venues where English is the preferred or only accepted language for journal article publishing.

We are certainly aware that science is being conducted and communicated in many languages other than English – for example please see our recent paper showing the importance of such non-English-language science in biodiversity conservation (Amano et al 2021 PLOS Biol: <https://doi.org/10.1371/journal.pbio.3001296>). Nevertheless, it is also true that English now plays a dominant role in conducting, communicating and evaluating science in most disciplines and therefore we believe that our argument applies to almost any disciplines in science. For increased clarity, we have added an explanation on this point in L56-58.

Comment #5

In line 87 you mention reading as "a requisite for obtaining necessary knowledge in research". Please keep in mind scientific literature that is published in languages other than English in local and regional journals and repositories.

As described above, we recognise the importance of non-English-language literature and have, in fact, advocated its use in science, especially considering the current underuse of non-English-language literature at the international level (see our recent paper too: Amano et al 2023 Nat Sustain: https://doi.org/10.1038/s41893-023-01087-8). However, that does not necessarily mean that we can obtain all necessary knowledge in research only from literature written in a non-English language. Unfortunately, the reality at present is that much important knowledge, especially on cutting-edge research, is available only in English and we therefore believe that reading English-language literature is indeed a requisite for obtaining necessary knowledge in research. For increased clarity, we have modified the phrase as “*a requisite for obtaining necessary, especially cutting-edge, knowledge in research*” (L97).

Comment #6

Terms such as "Global North" and "Global South" carry substantial weight in assumptions for economic versus geographical attributes and might be misleading or inappropriate especially in the context of this article. I would suggest to instead be naming countries directly or referring to specific geographical regions and continents and/or populations of particular language groups.

This is a very good point – thanks very much for raising this. Following the suggestion, we have removed the phrase including the word “Global North” (L52).

Comment #7

In line 40 and 47 you mention "under-represented communities/groups". Could you please specify what you mean by that and in reference to whom? The argument seems to go beyond the distinction between native and non-native English speakers.

This is another good point. We believe that non-native English speakers are currently under-represented in terms of their participation in and contribution to science (we have now briefly mentioned this in L238-239). But we have avoided explaining this in the Introduction, as under-representation itself is not the direct focus of this study (it is rather a consequence of the disadvantages we quantified in this study).

This part of the text explains why it is important to remove barriers for disadvantaged, and thus under-represented, groups of researchers (i.e., not only non-native English speakers). We thus decided to keep the expression broad and general, and have replaced “under-represented” in L43 and 50 with “disadvantaged”, as we think it would specify the issue of focus in this study more appropriately. We have removed “under-represented” from L39, as we feel it’s unnecessary.

Comment #8

While it is important to highlight disadvantages, the manuscript could benefit from mentioning more explicitly the advantages of multilingualism in a globalized world and advocating for multilingual research practices to be adopted by all researchers, non-native English speakers and native English speakers alike.

We totally agree with Reviewer #2 here and have explained the advantages of multilingualism in science in Discussion as “*A key aspect of those solutions is to embrace linguistic diversity in science and encourage the multilingualization of science and its communication, as this can help to improve equity, diversity, and inclusiveness in science [14] and maximise the contribution of science to addressing some of the global challenges [32, 33]*” (L287-290).

Comment #9

What is striking to me is that non-native English speakers spend less time in preparing presentations or manuscripts in their first language as compared to native English speakers, in each of their native language (line 176ff). Were you able to identify explanations for this?

We have also found it interesting but to be honest have no convincing explanation for this. The pattern was not necessarily consistent across nationalities (those with low English proficiency nationalities indeed spent significantly less time than native English speakers, but those with moderate English proficiency nationalities did not; see Table S16). Therefore we have decided not to add any discussion on this.

Comment #10

Additional comments

Thank you for also specifying author contribution according to the CRediT taxonomy.

From my personal experience and that of many of my colleagues, it could also be inquired and highlighted how non-native English speakers lose the skill to explain their work in their native language, once they fully emerged in the English language to conduct and communicate about their research in and what effect that might have on science literacy in any part of the world irrespective of economic capacity; perhaps to consider in follow-up investigations and discussions.

This is another great suggestion, thank you. We definitely agree that non-native English speakers face this dilemma, and have now explained it as an additional consequence of language barriers for non-native English speakers (L263-265).

**In response to Reviewer #3’s comments**

Comment #1

This article describes the various disadvantages in the current international scientific system faced by scientists who are not native English speakers. The results are not surprising: it takes non-native English speakers longer to read articles and to write articles, and their articles are more likely to be rejected. And non-native English speakers tend to avoid English-language conferences and take more time to prepare talks. But while the results are not surprising, the paper provides quantitative information on a problem that is pervasive and generally known in the field.

Thank you very much for your time to review our manuscript and the positive evaluation. We agree – the results may not be surprising, but we believe providing quantitative evidence on disadvantages for non-native English speakers is important to stimulate action to address this overlooked problem in scientific communities.

Comment #2

The abstract should mention that the people surveyed are in the field of environmental science.

We have now described the detail of the survey including our focus on environmental sciences (L31-33).

Comment #3

The sample sizes are not large, but they are reasonable and adequate.

Thank you.

Comment #4

The results are presented in a clear way that makes the problem easy to understand. The quantitative results are easy to see, though the authors may want to reduce the number of decimal places being reported, 46.64 is probably one too many decimal places.

We agree and have now reduced the number of decimal places throughout the main text.

Comment #5

Figure 1 is not very effective. For example, let's consider the first item of the time needed to read a paper. Most of the right side of the figure is empty as the number of people writing more than 50 papers is very small. Each of these figures need to be redone, perhaps by lumping together all of the people with more than 50 papers into one group.

We appreciate this helpful suggestion. In the previous version we had square-transformed the x-axis, but considering the suggestion by Reviewer #3 here, we have now log_10_-transformed the x-axis in Figs. 1, 2 and 4, which we believe has made it much easier for readers to inspect the part of the figures with the number of English papers below 50.

Comment #6

In the Discussion, the point about survivor bias is very good. The people who have remained in the field and continued to publish are likely the people who have had better English language skills from the start, who have learned English over time, or have figured out how to get help, like language services or collaborators. The authors also make the point that even within a country, there is a variability in language skills and opportunities; even in a poor country where will be some well-off families with excellent education opportunities for their children.

Thanks very much for the positive evaluation here.

Comment #7

An important part of improving the current system would be to suggest solutions. Some of these are provided in the Discussion, but some of the more specific suggestions are only in Table S18 where most readers will not see them. The authors should try to bring this table into the main paper and mention some of the points in the Discussion. In particular, the authors might consider mentioning the idea of "buddies".

Thank you very much for this suggestion. We totally agree and have now re-written the whole paragraph about potential solutions (L279-298). We have also converted Table S18 into infographic and now present it as Fig. 6 in the main text. We hope this has successfully highlighted the solution part, as Reviewer #3 suggested here. In Fig. 6 we have also mentioned the idea of a “buddy” system as a potential solution that can be adopted by supervisors/collaborators, journals, and conferences.

Please also note that we have replaced previous Fig. S6 with new Fig. 5 – another infographic that visualises the estimated multiple disadvantages for non-native English speakers.

Comment #8

The journal Biological Conservation has had various article in recent years that consider this same topic, but from a different perspective and a different methodology. The authors should consult these papers, and if appropriate, incorporate them into their paper.

Maas B, RJ Pakeman, L Godet, L Smith, V Devictor, RB Primack. 2021. Women and Global South strikingly underrepresented among top-publishing ecologists. Conservation Letters 14 (4), e12797

This study found a striking under-representation of top-publishing authors from non-English speaking countries

Primack, R.B. and L. Zipf. 2016. Editorial: Acceptance rates and number of papers in Biological Conservation from 2005 to 2014 for Australia, Brazil, China, India, Spain, and the United States: Trends or noise? Biological Conservation 196:50-52.

There is a low acceptance rate of papers from China and other countries, but the acceptance rate for China is increasing over time.

Primack, R.B., Marrs, R., 2008. Bias in the review process. Biological Conservation
141, 2919-2920

There is a strong disadvantage in getting papers accepted for non-English speakers, including even for high-income countries like France and Germany.

Thank you very much for directing us to these useful references. We agree that some of our results support the findings of those papers and thus have cited those papers (as Refs 25, 26, and 28, together with another relevant papers (Ref 27) we found after the initial submission of this manuscript) in L146-148 and L238-239.
