# Supplementary material for: The manifold costs of being a non-native English speaker in science
Source: PLoS Biol. 2023 Jul 18;21(7):e3002184. doi: 10.1371/journal.pbio.3002184 (PMC10353817; doi:10.1371/journal.pbio.3002184)
Supplement: Attachment — Submitted filename: Responses020623.docx [file pbio.3002184.s035.docx]

**Response to the editor for "** **The cost of being a non-native English speaker in science" (PBIOLOGY-D-22-02711R2)**

----------------------------------------------------------------------------------------------------------------

**In response to Editor’s comments**

**Comment #1**

a) While the nature of the costs of being a non-native English speaker are nuanced, it's clear that these are multiple and diverse, so we suggest that you make the title more emphatic, as follows: "The manifold costs of being a non-native English speaker in science"

We have changed the title into the suggested one (L1).

**Comment #2**

c) Please address my Data Policy requests below; specifically, we need you to supply the numerical values directly underlying Figs 1ABCDEF, 2ABCD, 3AB, 4ABC, S1, S2, S3, S4, S5, S6, S7, S8, S9, either as a supplementary data file or as a permanent DOI’d deposition. We note that you have already deposited code and some data in OSF; please clarify the relationship between this and the Figures in this paper (I do understand that the raw survey data are protected by the agreement with the ethics office, and are therefore exempt from PLOS’ data policy).

As discussed separately, we have now provided numerical values underlying those figures that only plot summarised data (Figs. 2B, 3A, 3B, 4C, S1, S2, S4, S5, S6, S7, S8, and S9) as S1 Data, while for the other figures that directly plot survey answers (Figs. 1A, 1B, 1C, 1D, 1E, 1F, 2A, 2C, 2D, 4A, 4B, and S3), we have explicitly explained that our ethics approval prevents us from sharing those data to secure confidentiality of the respondents (see **Data and materials availability**).

**Comment #3**

d) Please cite the location of the data clearly in all relevant main and supplementary Figure legends, e.g. “The data underlying this Figure can be found in S1 Data” or “The data underlying this Figure can be found in https://doi.org/10.5281/zenodo.XXXXX” https://osf.io/XXXXX

We have now cited the location of the data, or explained that our ethics approval prevents us from sharing the data, in all relevant main and supplementary Figure legends.

**Comment #4**

As you address these items, please take this last chance to review your reference list to ensure that it is complete and correct. If you have cited papers that have been retracted, please include the rationale for doing so in the manuscript text, or remove these references and replace them with relevant current references. Any changes to the reference list should be mentioned in the cover letter that accompanies your revised manuscript.

We have now reviewed the reference list and removed some unnecessary information while updating Refs 27 and 28 with volume and page numbers.

**Other changes that we have made:**

- As the calculation of the data plotted in Figs. S1, S2, and S5 involves random processes (i.e., deriving numerous random numbers based on the estimated regression lines and associated errors, and calculating differences between participants with different linguistic backgrounds), the data prepared for publication (now in S1 Data) are only slightly different from those used to plot the previous version of Figs. S1, S2, and S5. Therefore, we have replaced the old version of Figs. S1, S2, and S5 with the new version based on the data provided in S1 Data. This does not affect the overall conclusion, as you can see that the old and new versions are almost identical. However, it (only slightly) affected the calculation of the exact estimates of the time required to read an English-language article (shown in the legend of Fig. S1) and read 200 articles per year (shown in the legend of Fig. S1 and L220). Therefore we have modified these estimates.
- We felt that it is important to emphasise the difficulty in defining one’s first language, and thus modified the sentence in L361-362

from “*defined as “the language(s) you learnt to speak at home as a child”*”

to “*defining participants’ first language is admittedly difficult in some countries, such as Nigeria, but we used the following definition: “the language(s) you learnt to speak at home as a child”*”.
